# A chromosome-level genome assembly of *Agave hybrid* NO.11648 provides insights into the CAM photosynthesis

**DOI:** 10.1093/hr/uhad269

**Published:** 2023-12-19

**Authors:** Ziping Yang, Qian Yang, Qi Liu, Xiaolong Li, Luli Wang, Yanmei Zhang, Zhi Ke, Zhiwei Lu, Huibang Shen, Junfeng Li, Wenzhao Zhou

**Affiliations:** Zhanjiang Key Laboratory of Tropical Crop Genetic Improvement, South Subtropical Crops Research Institute, Chinese Academy of Tropical Agricultural Sciences, 524091 Zhanjiang, Guangdong, China; Zhanjiang Key Laboratory of Tropical Crop Genetic Improvement, South Subtropical Crops Research Institute, Chinese Academy of Tropical Agricultural Sciences, 524091 Zhanjiang, Guangdong, China; Wuhan Onemore-tech Co., Ltd, 430076 Wuhan, Hubei, China; Biomarker Technologies Corporation, 101300 Beijing, China; Zhanjiang Key Laboratory of Tropical Crop Genetic Improvement, South Subtropical Crops Research Institute, Chinese Academy of Tropical Agricultural Sciences, 524091 Zhanjiang, Guangdong, China; Zhanjiang Key Laboratory of Tropical Crop Genetic Improvement, South Subtropical Crops Research Institute, Chinese Academy of Tropical Agricultural Sciences, 524091 Zhanjiang, Guangdong, China; Zhanjiang Key Laboratory of Tropical Crop Genetic Improvement, South Subtropical Crops Research Institute, Chinese Academy of Tropical Agricultural Sciences, 524091 Zhanjiang, Guangdong, China; Zhanjiang Key Laboratory of Tropical Crop Genetic Improvement, South Subtropical Crops Research Institute, Chinese Academy of Tropical Agricultural Sciences, 524091 Zhanjiang, Guangdong, China; Zhanjiang Key Laboratory of Tropical Crop Genetic Improvement, South Subtropical Crops Research Institute, Chinese Academy of Tropical Agricultural Sciences, 524091 Zhanjiang, Guangdong, China; Zhanjiang Key Laboratory of Tropical Crop Genetic Improvement, South Subtropical Crops Research Institute, Chinese Academy of Tropical Agricultural Sciences, 524091 Zhanjiang, Guangdong, China; Zhanjiang Key Laboratory of Tropical Crop Genetic Improvement, South Subtropical Crops Research Institute, Chinese Academy of Tropical Agricultural Sciences, 524091 Zhanjiang, Guangdong, China

## Abstract

The subfamily Agavoideae comprises crassulacean acid metabolism (CAM), C3, and C4 plants with a young age of speciation and slower mutation accumulation, making it a model crop for studying CAM evolution. However, the genetic mechanism underlying CAM evolution remains unclear because of lacking genomic information. This study assembled the genome of *Agave hybrid* NO.11648, a constitutive CAM plant belonging to subfamily Agavoideae, at the chromosome level using data generated from high-throughput chromosome conformation capture, Nanopore, and Illumina techniques, resulting in 30 pseudo-chromosomes with a size of 4.87 Gb and scaffold N_**50**_ of 186.42 Mb. The genome annotation revealed 58 841 protein-coding genes and 76.91% repetitive sequences, with the dominant repetitive sequences being the I-type repeats (Copia and Gypsy accounting for 18.34% and 13.5% of the genome, respectively). Our findings also provide support for a whole genome duplication event in the lineage leading to *A. hybrid*, which occurred after its divergence from subfamily Asparagoideae. Moreover, we identified a gene duplication event in the phosphoenolpyruvate carboxylase kinase (*PEPCK*) gene family and revealed that three *PEPCK* genes (*PEPCK3, PEPCK5,* and *PEPCK12*) were involved in the CAM pathway. More importantly, we identified transcription factors enriched in the circadian rhythm, MAPK signaling, and plant hormone signal pathway that regulate the *PEPCK3* expression by analysing the transcriptome and using yeast one-hybrid assays. Our results shed light on CAM evolution and offer an essential resource for the molecular breeding program of *Agave* spp.

## Introduction

The *Agave* genus, which comprises approximately 166 species, is known for its long-lived, monocarpic, and xerophytic (or succulent) nature. As a model crassulacean acid metabolism (CAM) crop, it thrives in extremely hot and drought environments. *Agave* genus is the largest genus in the subfamily Agavoideae (Asparagaceae), which consists of nine genera and about 300 species [1, 2]. Most commercially exploited species used for obtaining beverages, fibers, foods, and medicines came from the *Agave* genus [3, 4]. *Agave* spp. originated in the Americas and has served human communities for over 10 000 years [5]. It is now globally dispersed in subtropical and tropical areas, with a total harvested area of approximately 236 481 hectares and an annual production of approximately 220 372 tons (FAO, 2021).

CAM is an essential metabolic pathway that enables many plants to adapt to extreme environments, such as drought, low CO_2_, and/or high temperature [6]. Approximately 6% of flowering plant species have been found to exhibit CAM [7], which is frequently related to arid habitats. However, CAM also presents in epiphytes and a few aquatic species [8, 9]. By using an inverse (compared to C4 and C3) night/day model of stomatal opening/closure, CAM plants can nocturnally assimilate CO_2_ through open stomata and store it as malic acid in the vacuole. They keep their stomata closed and release the stored malic acid during the day and fix it by ribulose-1,5-bisphosphate carboxylase/oxygenase via the Calvin-Benson-Bassham cycle [10]. The operational degree of CAM may vary greatly depending on the evolutionary history of a given species and their environmental contexts. Facultative CAM plants have the ability to revert to CAM under abiotic stress, while constitutive CAM plants rely on the CAM pathway through the entire life cycle [5,11]. CAM plants maximize water use efficiency by taking up most CO_2_ at night, compared with the C3/C4 plants. All genes required for the CAM pathway exist in C3/C4 species, with similar core biochemical characteristics to those for C3/C4 plants [12]. Previous genomic and/or transcriptomic studies have analysed the evolutionary plasticity and diel re-programming of CAM-related gene expression in a few species [8, 12–13]. *Agave* spp*.* constitutively express CAM-related genes, and transcriptomic studies have also been reported among some *Agave* spp*.* [9, 14]. However, absence of genome information has rendered a valuable comparison of *Agave* spp. with other CAM species impossible.

Genome sequencing is currently considered an essential and crucial step for clarifying the molecular mechanisms underlying trait formation and optimizing breeding strategies. However, whole genome sequencing of *Agave* spp. has been hampered by the largeness and/or complexity of *Agave* genomes, which are estimated to be between 3.8 and 11.3 Gbp, with a high level of duplicity due to polyploidy levels (2–8×) and a high number of repetitive elements [15, 16]. Additionally, pure line breeding of *Agave* spp. is challenging due to its long maturation cycle (6–30 years) and its perennial, monocarpic growth mode [17]. In recent years, newly developed sequencing approaches, such as Oxford Nanopore Technology (ONT), have been employed to generate >10 kb reads with notably reduced sequencing costs. Chromosome conformation capture (Hi-C) technology has also been applied to unravel some large and complex plant genomes and assemble chromosome-level genomes [18, 19].

This study utilized Hi-C and ONT technologies to assemble a genome of *Agave hybrid* NO.11648 [(*Agave amaniensis × Agave angustifolia*) *× A. amanuensis*], the only commercial high-yielding hybrid cultivated in China [3] at chromosome-level. Comparative genomic and phylogenetic analyses were used to detect the genomic evolution of *A. hybrid* across many lineages. We also discussed the evolution of the CAM pathway of *A. hybrid*. Our results provided the first genome resources for research in the field of biology and genetic improvement of *Agave* spp.

## Results

### Karyotype of *A. hybrid*

We performed karyotype analyses of at least 20 cells to examine the mitotic metaphases of root tip. We observed 60 chromosomes, with lengths ranging from 0.5 to 12 micrometers, among which 10 are ultra-long chromosomes and the others are short chromosomes with centromeres located near the middle or the end. (Fig. S1, see online supplementary material). Consistent with previous reports on karyotype determination in *Agave* spp. [15, 20], *A. hybrid* chromosomes showed typical characteristics with 25 small and five large pairs of homologous chromosomes. We also observed two 5S rDNA hybridization signals in *A. hybrid* chromosomes (Fig. S1D, see online supplementary material)*.* Therefore, *A. hybrid* was diploid with 2n = 2x = 60.

### Estimation of genome size, repeat content, and heterozygosity

The flow cytometry analysis shows that the genome size of *A. hybrid* is 4.14 Gb (Table S1, see online supplementary material). To estimate the genome size, repeat content, and heterozygosity repeat content, we performed whole-genome sequencing, which generated 114.87-fold (488.22 Gb) Illumina clean data (Table S2, see online supplementary material). Using the total of 377 139 903 527 of 21-k-mers, we identified the dominant peak at a depth of 86 (Table S3; Fig. S2, see online supplementary material). The *A. hybrid* genome size was estimated to be 4.25 Gb with 80.29% repeats, and a heterozygosity rate of 0.42% (Table S3, see online supplementary material).

### Genome sequencing and assembling

The workflow for genome sequencing and assembling is illustrated in Fig. S3 (see online supplementary material). To generate a preliminary genome assembly of *A. hybrid* at the chromosome-level, the DNA of *A. hybrid* was sequenced using both PromethION and Illumina platforms to obtain single-molecule long reads and Hi-C data, respectively. We obtained 96.68-fold (410.91 Gb) ONT clean data with an N50 of 34.58 kb, maximum read length of 964.67 kb, and a read length of 28.12 kb in average (Tables S2 andS4, see online supplementary material). Following the methods described by Ou [21] and Wu [22], we generated a preliminary genome of 4.87 Gb, comprising 7279 contigs with an N50 of 1.06 Mb and a GC content of 38.36% (Table S5-Table S9, see online supplementary material). This assembly was longer than the estimated 4.25 Gb based on the k-mer analysis.

**Figure 1:**
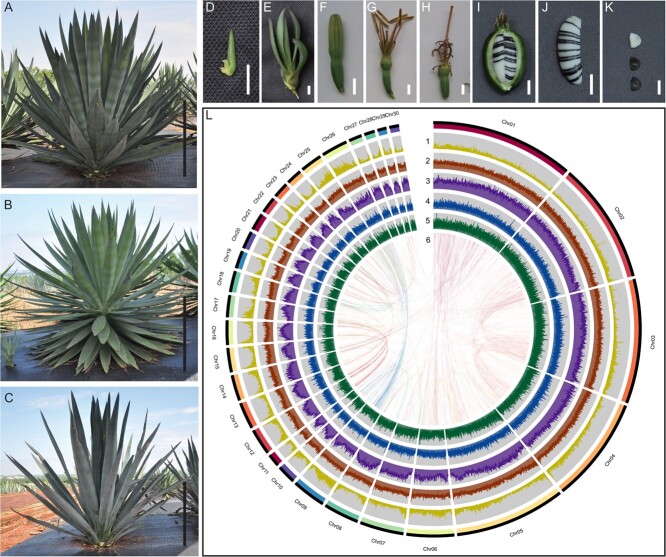
Plant morphology, genome features, and synteny information. **A***Agave hybrid* used in this study. The (**B***Agave angustifolia*) male parent and (**C***Agave amaniensis*) female parent of *A. hybrid*. The (**D**–**E**) bulbils, (**F**–**H**) flower, (**I**) capsules and (**J**–**K**) seeds of *A. hybrid.* White seeds were sterile seeds and black seeds were normal seeds. (**L)** Characterization of the elements in the super-scaffold of the *A. hybrid* genome. The figure illustrates (1) gene density, (2) GC content, (3) density of LARD, (4) density of Copia, (5) density of Gypsy, and (6) the relationship between syntenic blocks using sliding windows of 200 kb in 1 Mb intervals. Black bar (in **A**–**B**) = 50 cm, white bar (in **D**–**K**) = 1 cm.

We also generated 1 598 641 533 clean reads from the Hi-C libraries (Table S10, see online supplementary material) and used 108 951 411 valid interaction pairs to correct and improve the preliminary genome. This enabled 98.91% (4.82 Gb) of the preliminary genome to be successfully anchored into 30 pseudo-chromosomes (2n = 60, Fig. 1L, Fig. S4, Table S11-Table S13, see online supplementary material). The final genome assembly of *A. hybrid* at the chromosome-level was 4.82 Gb (94.55% of the preliminary genome) in length, carrying 1965 scaffolds with a N50 of 186.42 Mb (Tables S13 andS14, see online supplementary material). The 30 pseudo-chromosomes ranged from 36.41 to 584.22 Mb in size (Fig. 1L; Table S13, see online supplementary material).

The quality of the genome assembly was examined from four aspects. First, BUSCO analysis showed that 90.69% (1306) BUSCOs, including 39 fragmented BUSCOs, 1012 single-copy BUSCOs, and 294 duplicated BUSCOs, were identified in the assembly (Table S15, see online supplementary material). Second, up to 99.11% of the Illumina short sequences were aligned back to the assembly (Table S16, see online supplementary material). Third, the assembly covers approximately 99.33% of the expressed sequences in the *A. hybrid* transcriptome with >50% sequences identity (Table S17, see online supplementary material). Finally, the long-term repeat (LTR) assembly index (LAI) of *A. hybrid* was estimated to be 10.95, meeting the reference quality (Table S18, see online supplementary material).

### Genome annotation

The workflow for genome annotating is illustrated in Fig. S5 (see online supplementary material). We identified 3.75 Gb of repeat sequences making up 76.91% of the *A. hybrid* genome size (Table S19, see online supplementary material), lower than the value of 80.29% detected from the k-mer analysis. The major repeats were Copia (18.34%) and Gypsy (13.5%). Additionally, 34.26% ClassI/LTR could not be classified. We also identified a total of 9279 SSRs from the assembled genome (Table S19, see online supplementary material).

We identified protein-coding genes using AB initio, RNA-seq, and homology-based methods, which yielded 58 841 genes with transcript length of 9272.62 bp in average and CDS length of 1087.05 bp (Table 1; Fig. S6, Tables S20 andS21, see online supplementary material). Each gene had 4.59 introns and 3.59 exons with an intron length of 7978.87 bp and an exon length of 1293.75 bp in average (Table 1; Table S21, see online supplementary material). Among these protein-coding genes, 91.52% (53849) were functionally annotated in at least one public database (Table S22, see online supplementary material). To assess annotation accuracy, we aligned cDNA sequences from several tissues (root, stem, leaf, flower, fruit capsule, bulbil, and rhizome) to the predicted *A. hybrid* transcripts using TopHat [23]. The results showed that 77.18% and 10.00% of the cDNA sequences were mapped in the coding and intron regions, respectively (Table S23, see online supplementary material).

**Table 1:** Assembly and annotation statistics of the *A. hybrid* genome.

Category	Number/Length (bp)/Percent (%)
Genome assembly	
Assembly size (bp)	4 875 934 298
Total number of contigs	7499
Longest contig (bp)	7 500 000
Contig N50 (bp)	1 007 035
Total number of scaffolds	1965
Longest scaffold (bp)	567 149 452
Scaffold N50 (bp)	186 424 255
Gap length (bp)	548 400
GC content (%)	38.36
Genome features	
Number of protein-coding genes	58 841
Average gene/CDS length (bp)	9272.62 / 1087.05
Average exon/intron length (bp)	1293.75 / 7978.87
Total size of repeat	3 923 083 896
Repeat content (%)	80.46

CDS,codingsequence.

Furthermore, we identified and annotated three types of noncoding RNAs, including 291 miRNAs, 1718 rRNAs, and 1263 tRNAs (Table S24, see online supplementary material). Using GeneWise software, we predicted a total of 5264 pseudogenes by identifying immature stop codons and frameshift mutations (Table S25, see online supplementary material).

### Comparative genomic analysis

**Figure 2:**
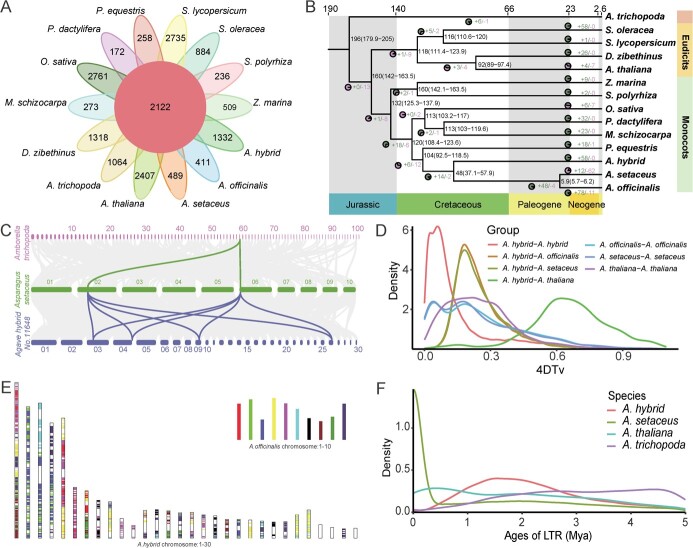
Phylogenetic and evolutionary analysis of the *Agave hybrid* genome, along with a comparative genomic analysis. **A** Venn diagram representing the distribution of shared gene families among *A. hybrid* and other 13 species*.***B** Phylogenetic tree of 14 species and gene family contraction and expansion compared with the related species (*Asparagus setaceus* and *Asparagus officinalis).* Gene family contractions and expansions are indicated in different colors, respectively. Inferred divergence times (million years ago, Mya) are denoted at each node in black. **C** Syntenic pattern among *Amborella trichopoda, A. setaceus*, and *A. hybrid* genomes. Each *A. trichopoda region* aligns with up to two regions in *A. setaceus*. Two homologous *A. setaceus regions* align with four regions in *A. hybrid.* Examples are highlighted in color. **D** 4DTv distribution in *A. hybrid* and other representative plant species. **E** Comparison of *A. hybrid* genome with *A. officinalis* genome. **F** The insertion dates of repeats in *A. hybrid* genome*.*

We aligned the protein-coding genes of 14 species to identify orthologous genes and assign gene families. Considering the evolutionary position of species, the quality of genome data, and the methods of obtaining genome data, we selected basal angiosperms (*Amborella trichopoda*), dicots (*Arabidopsis thaliana*, *Durio zibethinus*, *Spinacia oleracea*, and *Solanum lycopersicum*), and monocots (*Zostera marina*, *Asparagus officinalis*, *Asparagus setaceus*, *Phalaenopsis equestris*, *Phoenix dactylifera*, *Oryza sativa*, and *Musa schizocarpa*) for comparative genome analysis. Among these species, *A. officinalis*, *A. setaceus*, *P. equestris* (belong to Orchidaceae), and *A. hybrid* belong to the Asparagales, with *A. officinalis*, *A. setaceus*, and *A. hybrid* belonging to the family Asparagaceae, having the closest phylogenetic relationship. Our analysis revealed that out of the predicted 58 841 genes of *A. hybrid*, 48 315 genes were classified into 18 445 gene families, of which 2122 were found in 14 analysed species and 1332 were unique to *A. hybrid* (Fig. 2A; Figs S7 andS8, see online supplementary material). KEGG enrichment analysis implied that the unique genes were dominantly related to pentose and glucuronate interconversions, carbon fixation in photosynthetic organisms, and sesquiterpenoid and triterpenoid biosynthesis (Fig. S9, see online supplementary material).

We built a phylogenetic tree using the concatenated sequence alignment of 212 single-copy genes shared by *A. hybrid* and the other 13 species. Our results confirmed that *A. hybrid* clustered with *A. setaceus* and *A. officinalis* in Asparagaceae*,* and the divergence time between the ancestor of *A. hybrid* and the ancestors of *A. setaceus* and *A. officinalis* was approximately 48 million years ago (Mya) (Fig. 2B; Figs S10 andS11, see online supplementary material).

We also analysed the contraction and expansion of gene families and revealed that 58 clusters (692 genes) were expanded in *A. hybrid*. KEGG enrichment analysis revealed that most of the expanded genes were enriched in fructose and mannose metabolism, plant-pathogen interaction, and pentose and glucuronate interconversion pathways (Fig. 2B; Fig. S12, see online supplementary material). GO analysis found that the expanded genes were clustered together in response to biotic stimulus, leaf senescence, and carbohydrate transport.

Based on the estimates of *K*a/*K*s ratio and positively selected genes, we identified 137 genes that probably experienced positive selection. The majority of these genes were classified in KEGG pathways related to purine metabolism, mismatch repair, DNA replication, homologous recombination, and non-homologous end-joining (Fig. S13, see online supplementary material). GO analysis found that majority of these genes were enriched in meiosis I, telomere maintenance, and mitotic recombination.

We observed two peaks of 4DTv in *A. hybrid* at the value of ~0.026 and ~0.059 (Fig. 2D). We also observed the divergence peaks (4DTv ~0.181 and ~0.182) for *A. hybrid vs. A. officinalis* and *A. hybrid vs.A. setaceus*. Comparing the peak positions suggested that *A. hybrid* had experienced two round of whole genome duplication (WGD) events after the divergence of *A. hybrid* and *A. officinalis* (or *A. setaceus*) (Fig. 2C). The result was verified by assessing the number of synonymous substitutions per synonymous site (Ks) and synteny analysis (Fig. S14, see online supplementary material). Two WGD events occurred at Ks of ~0.073 and ~0.189 in *A. hybrid*, and the divergence between *A. hybrid* and *A. officinalis* and between *A. hybrid* and *A. setaceus* occurred at Ks of ~0.548 and ~0.522, respectively. The occurrence of one-versus-three syntenic blocks suggested that two round WGD events have occurred in the genome of *A. hybrid* (Figs S15, S16 and S18)*.* Additionally, we observed chromosomal rearrangements occurred in the *A. hybrid* genome after the divergence of *A. hybrid* and *A. officinalis* (or *A. setaceus*) (Fig. 2E; Fig. S17, see online supplementary material). Furthermore, we found that *A. hybrid* had a comparatively lower proportion but a longer time of older LTR insertions (Fig. 2F).

### Evolutionary analysis of genes involved in the CAM pathway

To investigate the evolution of photosynthesis from C3/C4 to CAM plants, we attempted to identify13 gene families related to carbon fixation in CAM, C3, and C4 plants, but the number of phosphoenolpyruvate carboxylase-related kinase (*PEPC-R*) genes was 0 in *A. hybrid*, so we actually identified 12 gene families, including 12 alpha carbonic anhydrases (*αCA*s), seven beta carbonic anhydrases (*βCA*s), eight gamma carbonic anhydrases (*γCA*s), four NADP-malate dehydrogenases (*NADP-MDH*s), 13 NAD-malate dehydrogenases (*NAD-MDH*s), seven NADP-malic enzymes (*NADP-ME*s), three NAD-dependent malic enzymes (*NAD-ME*s), four phosphoenolpyruvate carboxylases (*PEPC*s), 12 *PEPCK*s, three phosphoenolpyruvate carboxykinases (*PPCK*s), two pyruvate phosphate dikinases (*PPDK*s), and two pyruvate phosphate dikinase regulatory proteins (*PPDK-R*s) in the *A. hybrid* genome (Table S26, see online supplementary material). Moreover, *A. hybrid* had more *αCAs* and *PEPCK*s than C4 and C3 plants and, overall, had equal or greater numbers of genes in the 12 gene families compared to C4 and C3 plants.

Among the 12 gene families, *PPDK-R, PPCK,* and *PPDK* genes had a single origin in *A. hybrid* (Figs S19–S30, see online supplementary material). By contrast, the remaining genes originated from two or more clusters. PEPC proteins play a crucial role in binding CO_2_ in CAM plants. Thus, we used the sequences of PEPC proteins from 21 species to construct a phylogenetic tree (Fig. S24, see online supplementary material). Consistent with previous reports, PEPCs could be divided into bacterial-type and plant-type [8, 24]. Our ML phylogenetic tree analysis showed that all 12 *PEPCK*s of *A. hybrid* clustered together with *PEPCK*s from two other related species *(A. setaceus* and *A. officinalis)* (Fig. S27, see online supplementary material). Interestingly, two *PEPCK*s (EVM0039299 and EVM0005316) were clustered in the same clade with two *A. setaceus PEPCK*s and two *A. officinalis PEPCKs,* whereas the remaining 10 *PEPCK*s were clustered together with two *A. setaceus PEPCKs* (Fig. S31, see online supplementary material)*.* We found that nine of the 12 *PEPCK*s were distributed on four chromosomes, and the other three were located on contig06585, with chromosome 2 containing the largest number of *PEPCK*s (five genes) (Fig. S32, see online supplementary material). Additionally, a total of six tandem repeat blocks were identified in the *PEPCK* gene families, suggesting that the *PEPCK*s of *A. hybrid* have undergone gene expansion*.* Based on the current results, it can also be preliminarily inferred that the *PEPCK*s in the *A. hybrid* genome may participate in CAM modulation through gene dosage or duplication.

We compared the evolutionary changes in the number of 13 CAM pathway genes across 21 species (Fig. S33, see online supplementary material). For both monocotyledonous and dicotyledonous C3 plants, gene loss significantly outweighs gene gain. In the three C4 plants, gene loss also exceeded gene gain. The gene loss and gain in facultative plants (C3/CAM) were balanced. Overall, CAM plants had more gene losses than gains, but *Kalanchoe laxiflora* and *A. hybrid* had more gene gains than losses.

### Expression patterns of CAM pathway genes

**Figure 3:**
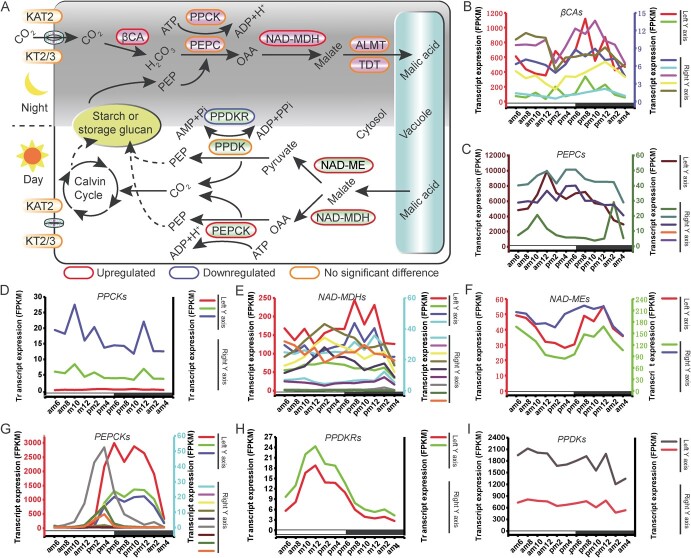
Key CAM pathway genes and their expression patterns in *Agave hybrid*. **A** Overview of the CAM pathway. The carboxylation is shown at the top and decarboxylation at the bottom. (**B**–**I**) Gene expression patterns at diel environmental conditions. βCA, beta carbonic anhydrase; KAT, potassium channel in *Arabidopsis thaliana*; KT, potassium transporter; NAD-malate dehydrogenase; NAD-ME, NAD-dependent malic enzyme; NAD-MDH, ALMT, tonoplast aluminum-activated malate transporter; OAA, oxaloacetate; PEPC, phosphoenolpyruvate carboxylase; PEPCK, phosphoenolpyruvate carboxylase kinase; PPCK, phosphoenolpyruvate carboxykinase; PPDK, pyruvate phosphate dikinase; PPDK-R, pyruvate phosphate dikinase regulatory protein; TDT, tonoplast dicarboxylate transporter. White and black bars indicate daytime (12-h) and nighttime (12-h), respectively.

To identify the expression profiles of CAM-related genes under diel conditions, we analysed RNA-sequencing data by sampling photosynthetic leaf tissues of *A. hybrid* every 2 h over a 24-h period. The results revealed that the expression profiles of some CAM pathway genes in *A. hybrid* changed under diel conditions (Fig. 3; Fig. S34, see online supplementary material). For example, one *βCA* showed strong expression at night with peaks at 8 p.m. and 12 p.m. (Fig. 3B; Fig. S34, see online supplementary material). Similarly, four *NAD-MDH*s were expressed at a higher level at night than in the daytime, with peaks in the at 8 p.m. and 12 p.m. (Fig. 3E; Fig. S34, see online supplementary material). Although the expression of *PEPC*s and *PPCK*s peaked at 12 p.m. and 2 a.m., they did not exhibit significantly higher expression levels at night (Fig. 3C and D; Fig. S34, see online supplementary material).

Genes responsible for decarboxylation during the day, such as three members of *NAD-MDH*s, exhibit higher nighttime expression levels (Fig. 3E; Fig. S34, see online supplementary material). Unexpectedly, all *NADP-MDH*s both showed strong transcript abundance during the day and the night (Fig. S34, see online supplementary material). The expression levels of the three *NAD-ME*s gradually increased from dusk, peaking at midnight, and then began to decrease, with the lowest point occurring at 4 p.m. in the afternoon (Fig. 3F; Fig. S34, see online supplementary material). The two *NADP-ME*s exhibited diurnal peak expression, with the peak occurring at noon (Fig. S34, see online supplementary material). Despite abundant transcripts throughout the day and night, two *PPDK*s did not show diel expression patterns (Fig. 3I; Fig. S34, see online supplementary material). Similarly, we found that three *PEPCK*s showed stronger transcript abundance at night, and only one *PEPCK* was expression at a higher level during the day, increasing in the morning and peaking at 4 p.m. (Fig. 3G; Fig. S34, see online supplementary material). Additionally, we found that the expression of 5 *αCA* were at transcript level was elevated during the night than during the day (Fig. S34, see online supplementary material).

### Convergent evolution of PEPC

**Figure 4:**
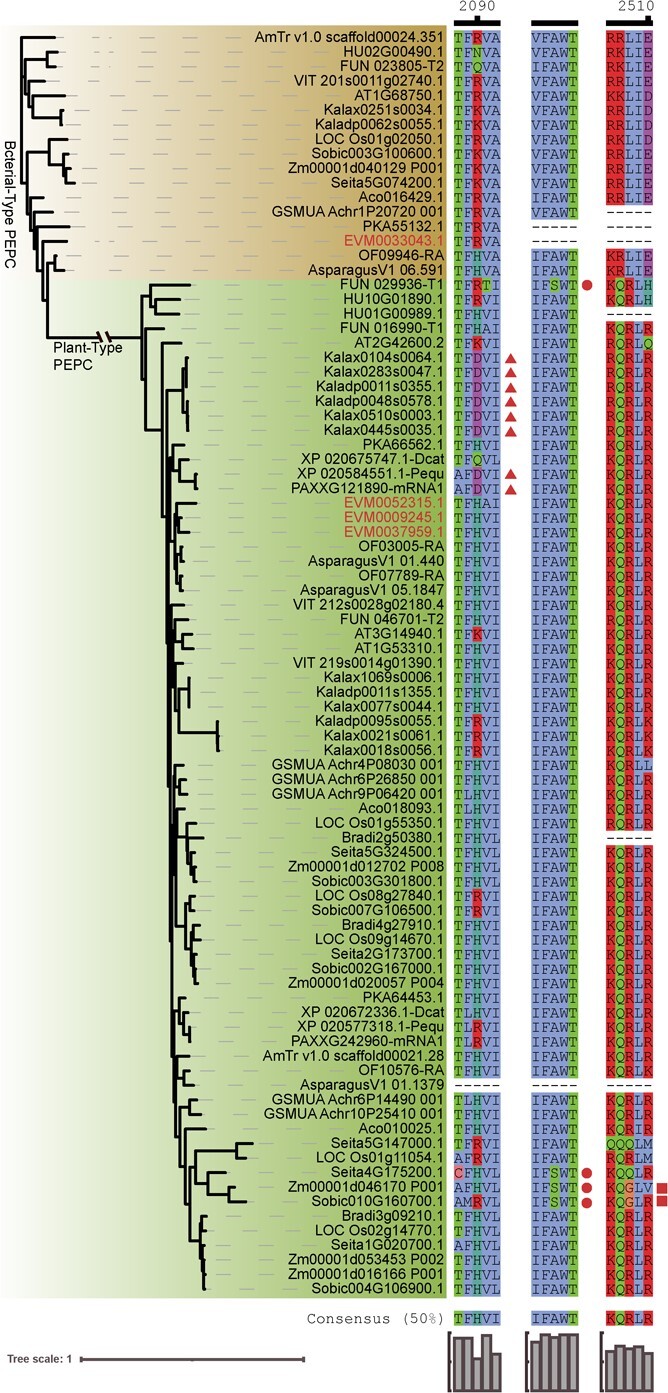
Convergent amino-acid change in PEPC shared by diverse species. Copies with putative convergent amino acid sequence (D at position 3 in alignment) are indicated by the triangle (from R/K/H to D), circle (from A to S), and square (from R to G).

Previous studies have found convergent changes in PEPC protein sequences in some plants. For example, a substitution from A to S and/or R to G led to significantly increased PEPC activity in some C4 plants [24]. Moreover, the substitution from R/K/H to D significantly increased PEPC activity in *P. equestris, Kalanchoe fedtschenkoi*, and *K. laxiflora* [8, 13]. We aligned PEPC protein sequences from 21 species and revealed that these convergent changes in PEPC proteins were not observed in *A. hybrid* (Fig. 4). By contrast, the convergent substitution from R/K/H to D was identified in *Hylocereus undatus* (a succulent and CAM fruit crop), and a mutation from A to S appeared in the PEPC (FUN_029936-T1) of *Portulaca amilis* (a CAM plant). To analyse whether the convergent changes exist in other *Agave* species, nine species and six *A. hybrid* varieties were collected for RNA-sequencing (Table S29, see online supplementary material), and transcriptome data of three species were collected from NCBI. These data were furtherly used to identify PEPC protein sequences. The analysis clearly indicated that the convergent changes were not detected in 12 species and six *A. hybrid* varieties (Fig. S35, see online supplementary material). These findings suggested that single amino acid mutations might be not critical in the evolution of PEPC in *A. hybrid*.

### Key genes involved in CAM pathway in *A. hybrid*

**Figure 5:**
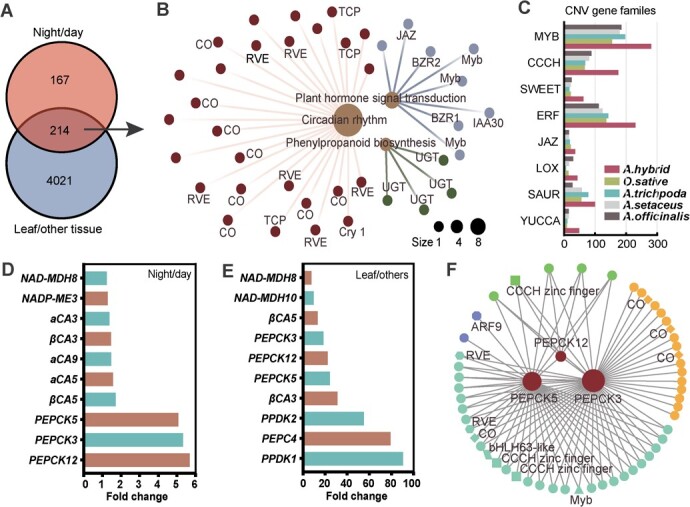
Transcriptional regulation of CAM pathway genes*.***A** Venn diagrams of differentially expressed genes between night and day and between leaf and other tissues. **B** KEGG functional classification of the 214 shared genes, with genes in key enriched KEGG pathways being visualized by different colors. **C** Copy number variations (CNV) of important gene families in *Agave hybrid*, *Oryza sativa*, *Arabidopsis thaliana*, *Asparagus setaceus*, and *Asparagus officinalis*. **D** Top 10 genes with higher expression levels at night than during the day. Bars indicate the expressional fold change of night/day, which calculated as: (night +10)/(day +10). **E** Top 10 genes with higher expression levels in leaves than in other tissues. Bars indicate the expressional fold change of night/day, which calculated as: (leaves +10)/(other tissues +10). **F** Co-expression network of three *PEPCK*s in the greenyellow module.

The temporal modulation of CO_2_ absorption and fixation is the major characteristic of CAM. To explore this phenomenon, we analysed the levels of genes in the leaf and other tissues during day and night periods. Using a screening criterion of a ratio greater than 1.5, we identified 4235 genes with higher levels in the leaf than in other tissues and 381 genes with higher transcript abundance at night than in the day (Fig. 5A). Further analysis revealed that 214 genes were expressed at a higher level in the leaf than in the other tissues and at night than during the day (Fig. 5A). These genes were primarily involved in circadian rhythm, CAM pathways, plant hormone signal transduction, and sugar metabolism (Fig. 5B).

In this cross set, three CAM gene*s* (*PEPCK3*, *PEPCK5*, and *PEPCK12*) showed a 5.57-fold, 5.23-fold, and 5.00-fold higher level at night than in the day, respectively, and a 21.86-fold, 18.31-fold, and 23.94-fold greater expression in the leaf than in the other tissues, respectively (Fig. 5D and E; Figs S36 andS37, Tables S27 andS28, see online supplementary material). Although other CAM genes, such as *PEPC*, were expressed at a higher level in the leaf than in other tissues, they did not exhibit significantly higher expression pattern at night than during the day. Additionally, we found several genes coding transcription factors were differentially expressed under diel conditions, including 28 genes involved in circadian rhythm (six *RVE*s, nine *CO*s, eight *Dof*s, and two *TCP*s), nine genes involved in plant hormone signal transduction (two *BES1/BZR1*s, one *JAZ*, one *AUX/IAA*), two genes involved in sugar metabolism (two *beta-amylase*s) (Fig. 5B). We also found that genes coding homeodomain proteins (*BEL1-like*, *knotted-1-like*), MADS-box, nuclear transcription factor Y, and Zinc-finger homeodomain protein were also differentially expressed (Fig. 5B). These results suggested that *A. hybrid* might achieve CAM by increasing the levels of *PEPCK*s, and genes involved in circadian rhythm, sugar metabolism, and plant hormone signal transduction might regulate the expression of genes in the CAM pathway.

### Identification of potential transcription factors that regulate of three *PEPCK*s by co-expression network analysis (WGCNA)

To investigate the potential molecular mechanisms in the regulation of three *PEPCK*s, we performed WGCNA of the transcriptome data based on all 8468 differentially expressed genes. The analysis revealed 13 co-expression modules (Figs S38–S41, see online supplementary material), three of which (greenyellow, darkorange2, and brown modules) were positively correlated with leaf samples collected during the night period. Co-expressed genes gathered in these three modules were primarily involved in starch and sucrose metabolism, circadian rhythm, and plant hormone signal transduction, including key genes encoding proteins involved in the CAM pathway, such as *PEPCK*s (*PEPCK3*, *PEPCK5*, and *PEPCK12*) in the greenyellow modules (Fig. S42, see online supplementary material). These results suggested that genes in these co-expression modules are crucial in the nighttime processes that define CAM. Alternately, some modules (sienna3, coral1, plum2, and lightcyan1) were associated with the leaf samples collected during the day, with an over-representation of several biological processes. Several key protein-encoding genes involved in CAM, such as *PPDK-R1* (EVM0018250), *PPDK-R2* (EVM0055112), *aCA1* (EVM0001152), *PEPCK1* (EVM0004631), *PEPCK4* (EVM0024279), *PEPCK9* (EVM0044648), *PEPCK10* (EVM0045210), and *PEPCK11* (EVM0048393), were also identified in these modules (Figs S43–S45, see online supplementary material).

Further analysis of the greenyellow modules using WGCNA showed that 54 genes were co-expressed with *PEPCK3*, *PEPCK5*, and *PEPCK12* (Fig. 5F). Notably, the co-expression network of the three *PEPCKs* in these modules was involved in circadian rhythm, as indicated by the presence of *CONSTANS* (*CO*) and *REVEILLE* (*RVE*) genes. Additionally, several transcription factors, including the auxin response factor (ARF), Myb, bHLH, and CCCH zinc finger, were identified in this co-expression network*.* Three *CO* genes and one *ARF9* gene were found to exhibit single co-expression with *PEPCK3* and *PEPCK5*.

Comparative analysis of *A. thaliana*, *O. sativa*, *A. setaceus*, and *A. officinalis* revealed that certain gene families, such as *Myb* (49) and *LOX* (43), involved in IAA and JA synthesis and *SAUR* (100), *ERF* (230), and *JAZ* (36) gene families associated with IAA, ETH, and JA signaling pathway, have undergone gene expansion (Fig. 5C). Furthermore, six, nine, six, eight, and two tandem repeat blocks were identified in the *YUCCA*, *SAUR*, *ERF*, *LOX*, and *JAZ* gene families, respectively (Figs S46–S50, see online supplementary material). Interestingly, gene expansion and tandem repeat were also observed in the *SWEET* gene family (Fig. 5C; Fig. S51, see online supplementary material), which included 62 genes that regulate sugar efflux independently of energy or pH.

Overall, these findings suggest that transcription factors involved in hormone signals and circadian rhythm may be essential in regulating the expression of three *PEPCK*s (*PEPCK3*, *PEPCK5*, and *PEPCK12*) in *A. hybrid*.

### Identification of potential transcription factors involved in regulating *PEPCK3* using yeast one-hybrid system

**Figure 6:**
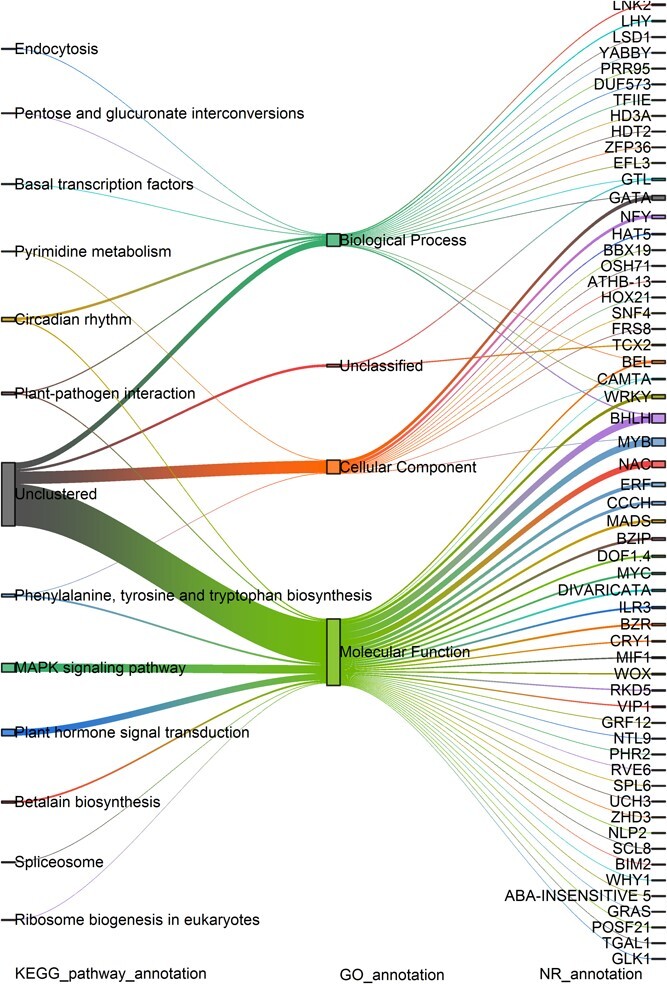
Clustering analysis of transcription factors. KEGG and GO functional classifications of 143 transcription factors are visualized in the left and middle panels, respectively. The abbreviations of transcription factors are shown on the right.

The yeast one-hybrid system was utilized to screen the transcription factors that may regulate *PEPCK*. A total of 143 transcription factors were identified through yeast hybridization using the *PEPCK3* promoter as the bait (Fig. 6; Fig. S52, see online supplementary material). These transcription factors were clustered in three GO and twelve KEGG pathways, encompassing 58 protein families. The top three KEGG pathways were the MAPK signaling pathway, circadian rhythm pathway, and plant hormone signal transduction pathway, based on the analysis of protein functions. The MAPK signaling pathway contained 13 proteins, including four WRKYs, three MYCs, two BHLHs, and one bZIP transcription factor. The transcription factors clustered in plant hormone signal transduction were mainly involved in the ABA and BR signaling, including three BZR and one ABA-INSENSITIVE transcription factor. A total of ten transcription factors were classified under circadian rhythm, including EFL3, PRR9, LNK2, LHY, CRY, and RVE. Based on the number of protein families, BHLH, MYB, NAC, ERF, CCCH, GATA, NYF, BEL, WRKY, and bZIP were the top ten most abundant transcription factors. These findings suggest that multiple pathways may regulate the expression of *PEPCK3* in *A. hybrid*.

## Discussion

*Agave* spp. are native to arid regions and are adapted to extreme heat and drought environments as constitutive CAM plants. Previous transcriptomics studies have attempted to uncover the molecular mechanism underlying the CAM and drought tolerance evolution in *Agave* spp., but the lack of genomic sequence information has limited molecular investigations. The genome sequences yielded in this study offer a relevant reference for molecular research in the *Agave* spp. The genome data were utilized not only for plant comparative genomics and evolutionary research but also for genome-wide analysis of convergent evolution and the regulation of CAM plants.

### Evolution of CAM-related genes in *A. hybrid*

CAM plants exhibit temporal regulation of CO_2_ absorption and fixation, which distinguishes them from the C3 and C4 plants [10]. Previous studies have found altered expression of CAM-related genes under diel conditions in multiple plants [8, 12–13]. Although a few information has been reported in *Agave* spp., data were collected from unreferenced transcriptome [9, 13]. This study revealed CAM pathway genes in *A. hybrid* and compared their expression under diel conditions. We found higher expression levels of *PEPCK3*, *PEPCK5*, *PEPCK12*, *βCA3*, *βCA5*, *aCA3*, *aCA5*, and *aCA9* in the night (Fig. S35, see online supplementary material), suggesting their involvement in the CAM photosynthetic pathway.

Previous studies have reported that βCA was the primary enzyme responsible for converting atmospheric CO_2_ to HCO_3_ in CAM and C4 plants [8, 12–13]. Our results showed that three *aCA*s, in addition to *βCA*s, showed a nocturnal higher expression profile (Fig. S32, see online supplementary material), consistent with a previous study on *Agave* spp. [11]. Additional analysis is required to ascertain the independent origin of aCA and its role in CO_2_ fixation.

We identified four *PEPC*s in *A. hybrid*, with *EVM0052315* showing the highest transcript abundance in midday (Fig. 3C; Fig. S32, see online supplementary material). Two other *PEPC*s (*EVM0033043* and *EVM0042231*) showed transcriptional peaks at night, with 2-fold and 4-fold changes in expression, respectively. In our study, the diurnal changes in peak *PEPC* expression are noteworthy and in line with previous work in *Agave* spp. [11]. These results indicated that carbon fixation in *A. hybrid* occurred within a narrow time frame at night via the PEPC-MDH pathway.

On average, C3, C4, and CAM plants have 2.3, 3.33, and 4.13 *PEPCK*s (Table S26, see online supplementary material), respectively. Among C3, C4, and CAM plants, *Musa acuminate*, *Zea mays*, and *K. laxiflora* have the largest number of *PEPCK*s, with 6, 4, and 8, respectively. Here, we identified 12 *PEPCK*s, in *A. hybrid* genome with three independent origins (Table S26, see online supplementary material). Among them, three genes showed a shift in expression from morning to night, with alternative peak expression (Fig. 3G; Fig. S32, see online supplementary material). These results indicated that gene duplication occurred in the *PEPCK* gene family, leading to changes in gene expression profiles. Furthermore, it implied that CAM regulation in this genome might be influenced by gene dosage and gene duplication.

### Switch of C3-to-CAM in *A. hybrid*

CAM photosynthesis has independently evolved multiple times from C3 photosynthesis in various plant lineages, possibly as an adaptation to decreases in atmospheric CO_2_ concentration on a global scale and water limitations [6,7]. In the subfamily Agavoideae, CAM has been found to have originated from three independent events [25].

One hypothesis suggests that the origin of CAM is associated with the duplication of multiple gene families [9, 10]. Our findings support this hypothesis in CAM plants, as we identified 12 *PEPCK*s (Table S26, see online supplementary material), with more copy numbers in *A. hybrid* than in C3 and C4 plants. Based on our comparative genomic analysis, *PEPCK*s duplication may have resulted from WGD and chromosome rearrangement. Another hypothesis suggests that the origin of CAM may have resulted from adapting the diel expression patterns of CAM genes [8, 10, 12]. Our findings also support this hypothesis in CAM, as several CAM pathway genes, including *PEPCK*s, exhibited nocturnal expression patterns, similar to previous reports in other CAM plants (Fig. 3G; Fig. S32, see online supplementary material). We also found that three *PEPCK*s exhibited the highest nocturnal expression abundance in *A. hybrid*. According to current research reports, the evolution of plant CAM either occurs by increasing gene dosage or by regulating the expression of CAM pathway genes, with no reports of both evolutionary mechanisms appearing in a single species. However, in *A. hybrid*, we found evidence of both gene dosage and gene expression regulation acting on CAM pathway genes. Furthermore, these two evolutionary mechanisms are concentrated in a single gene family in the CAM pathway, namely the *PEPCK* family. This strong CAM evolutionary drive in *A. hybrid* may be the result of *A. hybrid* adaptation to extreme high temperatures and extreme drought conditions. Our findings indicate that gene dosage and gene expression regulation can occur in the same CAM plant and are concentrated in the same gene family.

Two metabolic pathways can convert malate to PEP, one involving NADP^+^-/NAD^+^-ME, which converts malate to pyruvate and then to PEP by PPDK-mediated pyruvate decarboxylation, and the other involving NADP^+^-/NAD^+^-NDH, which converts malate to OAA and then to PEP by PEPCK -mediated OAA decarboxylation. These reactions are thought to occur during the daytime. In orchids, *PPDK* exhibits high expression levels at dark, inferring that *PPDK* participates in CO_2_ fixation during the night via a reversible reaction that converts pyruvate to PEP [25]. In our study, we found that three of 12 *PEPCK*s showed significantly high expression at night than during the day (Fig. S35, see online supplementary material). Additionally, all four *NADP-MDH*s, which convert malate to OAA, kept high level expression both in day and night. Based on the expression profiles of *PEPCK*s and *NADP-MDH*s, we propose that in the dark, PEPCK converts PEP to malate. While this reversible reaction needs to be further confirmed, analysing whether the reverse reaction is unique to CAM species in Asparaceae, Orchidaceae, and *Agave* or whether it exists in other plants can help us further comprehend the evolution of CAM.

### Regulation of *PEPCK* genes in *A. hybrid*

CAM and C3 photosynthesis share many enzymes for concentrating CO_2_ but differ in the timing of carbon fixation [6–7, 10]. One hypothesis suggests that altered diel expression profiles of key genes of CAM pathway might be the mechanism for evolution from C3 to CAM photosynthesis [8, 10, 12]. The circadian rhythm genes, temperature-regulated genes, light-regulated genes, and possibly nutrition-related genes may be crucial in the switch from C3 to CAM photosynthesis [6, 8, 12, 14, 25, 26]. However, the regulatory mechanisms may only conserve in some lineages. For example, the expression of CAM pathway genes was repressed or inactivated by the *trans*-acting elements in pineapple, and no circadian *cis*-regulatory elements were detected in the CAM genes of *Isoetes taiwanensis* [8]. In *K. fedtschenkoi*, researchers have observed a convergent mutation from E to R in the elongated hypocotyl 5 (HY5). This bZIP family transcription factor functions as an input to entrain the circadian clock [13]. Moreover, researchers have also observed altered expression patterns of *REVEILLE1* (*RVE1*) in the output subset of the circadian clock in *Agave americana* [14]. In this study, we found that transcription factors involved in plant hormone signal and circadian rhythm were co-expressed with CAM pathway genes (Fig. 5; Figs S40–S43, see online supplementary material). Furthermore, we identified transcription factors involved in MAPK signaling, plant hormone signals, and circadian rhythm pathway could bind to the promotor of the *PEPCK3* (Fig. 6; Fig. S50, see online supplementary material). Thus, studies on the regulatory relationship between these transcription factors and *PEPCK3* expression could provide insight into the switch from C3 to CAM photosynthesis.

### PEPC in *A. hybrid*

PEPC is a critical protein for CO_2_ fixation in the CAM pathway during the night. Previous genomic studies have reported that substitutions in PEPCs occurred during the evolution of C4 and CAM. A single amino acid mutation of A-to-S and/or R-to-G led to a significant increase in C4 PEPC enzyme activity [25], while the R-to-D mutation enhanced the activity of CAM PEPC [8, 13]. However, we only observed these substitutions in some PEPCs in *Agave* spp. (Fig. 4; Fig. S33, see online supplementary material). Instead, lysine (H) or arginine (R) appeared in this position, similar to PEPCs from many non-CAM species. This suggests that in *A. hybrid,* PEPC evolved differentially to enable PEPC enzymes to participate in the CAM photosynthetic pathway. Interestingly, we found that the A-to-S mutation occurred in *P. amilis* and the R-to-D mutation appeared in *H. undatus*, indicating that the A-to-S mutation not only occurs in C4 plants but also in CAM plants.

Three mechanisms for the evolution from C3 to CAM have been reported so far, including increasing the number of genes [ 9], changing gene expression patterns [12, 25], and amino acid site mutations [13]. Changing the expression pattern of key genes is considered to be the shortest pathway for C3-to-CAM evolution, and amino acid site mutations of key proteins is the best evolutionary pathway for evolving new protein functions while preserving the gene’s temporal expression pattern unchanged [13]. We have not seen any reports showing that two or more types of evolutionary mechanisms occurred in the same gene in any type of organisms to date (Fig. 4; Fig. S33, see online supplementary material). PEPC is a molecular marker for studying CAM evolution. A previous report showed gene duplication of PEPC in the Orchidaceae [9], but subsequent comparative transcriptome analysis did not support this conclusion. Instead, it was found that several genes including PEPC in the Orchidaceae underwent changes in their expression patterns, with their expression levels shifting towards nighttime [25]. Later, a typical nighttime-expressing CAM-type PEPC was found in *Kalanchoe*, and an R/K/H to D mutation was found to significantly increase PEPC enzyme activity [13]，and this mutation is distributed in multiple CAM plants. However, we did not find any evidence of PEPC undergoing the three evolutionary mechanisms in *A. hybrid*. Four *PEPC*s were identified in *A. hybrid*, but their number was not higher than that of C3, C4, or other CAM species. Although the expression of several *A. hybrid PEPC*s peaked at night (Fig. 3C; Fig. S32, see online supplementary material), their expression levels were relatively low and the increase in expression occurred within a narrow timeframe. We analysed the amino acid sequences of PEPC proteins in 12 species and six varieties of Agavoideae, and none of the amino acid site mutations that can increase PEPC enzyme activity found in CAM and C4 plants appeared in *A. hybrid* PEPC (Fig. 4; Fig. S33, see online supplementary material). We do not rule out the possibility that amino acid mutations might be found in other species of Agavoideae, or that *A. hybrid* PEPC might produce amino acid site mutations that increase enzyme activity at other sites. The three aforementioned site mutations that increase PEPC enzyme activity do not rely on PPCK phosphorylation [13]. PPCK binds to PEPC and phosphorylates it to activate PEPC enzyme activity [12, 13]. We noted a peak in *PPCK* expression at midnight, which appeared earlier than the peak in PEPC expression at 2 a.m. We also noted that the expression peaks of three *A. hybrid PEPC*s occur during the day, but their expression relative to themselves remains relatively high at night. We propose a hypothesis that *A. hybrid* PEPC proteins form a polyprotein complex to maintain the stability of the PPCK-PEPC complex or significantly increase PEPC enzyme activity. It is also possible that such complexes can both maintain the stability of the PPCK-PEPC complex and greatly increase PEPC enzyme activity, thereby enabling carbon fixation at night in the CAM pattern without changing the expression pattern of PEPC, without increasing the number of *PEPC*s, and without amino acid mutations occurring in PEPC proteins in *A. hybrid*.

In conclusion, our results offer a high-quality reference genome of *Agaves* spp. and suggest that WGD event and proliferation of LTR were the main factors contributing to the amplification of *A. hybrid* genome size. Both gene duplication and diel gene expression re-programming might conduce to the evolution of CAM photosynthesis. Our results also provide direct evidence that transcription factors, such as ELF3, BZR, and VIP, integrate plant hormone signal, circadian rhythm, and MAPK signaling pathway with the CAM pathway. These findings offer crucial insight for comparative, evolutionary, and functional genomics analyses of plants and significantly promote our understanding of CAM evolution.

## Material and methods

### Sample collection and preparation

*A. hybrid* (four years) was grown in the Agave germplasm garden located in the South Subtropical Crops Research Institute, Guangdong Province, China. Fresh juvenile leaves were collected from a single adult plant, washed with 75% alcohol to remove the impurity at the surface, and congealed in liquid nitrogen. RNAs were isolated from the rhizomes, roots, bulbils, stems, leaves, fruit capsules, and flowers using the Omega Plant RNA kit (Omega Bio-Tek, R6827). Genomic DNA was isolated from the juvenile leaves using the CTAB method. RNA and DNA integrity and purity were analysed using both Nanodrop Qubit (Thermo Fisher Scientific, Waltham, MA, USA) and 0.3% agarose gel electrophoresis. Offshoots were collected from the ends of mature rhizomes and maintained in shoal water until new lateral roots emerged for molecular karyotype analysis. Fresh yellow juvenile leaves (unopened inner leaves near the shoot tip) were crosslinked by immersing in 2% formaldehyde for Hi-C sequencing.

To capture changes in mRNA abundance in response to diel conditions, green (photosynthetic) tissues at the leaf tip were collected from five biological replicates every 2 h over a 24 h time course under a 12 h light/12 h dark photoperiod. For multiple sequence alignment, roots, stems, leaves, flowers, fruit capsules, bulbils, and rhizomes from nine species and six *A. hybrid* varieties were collected for RNA extraction and sequencing. These nine species were *A. americana* L., *Agave neglecta*, *Agave desmetiana* hort., *Agave fourcroydes* Lem., *Agave potatorum* Zucc., *Agave attenuata* Salm-Dyck, *A. amanuensis*, *Agave cantala* Roxb., *A. angustifolia* Haw. These six varieties were *A. hybrid* ‘nanya NO.1’, *A. hybrid* ‘yuexi No.114’, *A. hybrid* ‘76 416’, *A. hybrid* ‘H1002’, *A. hybrid ‘*S0908*’*, and *A. hybrid ‘*D06–556*’.* The abbreviated gene names are listed in the column information on taxa included in this study in Table S28 (see online supplementary material).

### Molecular karyotype analysis of *A. hybrid*

Flow cytometry, molecular karyotype and fluorescence *in situ* hybridization (FISH) assays were performed following the procedures described previously [15]. We observed at least 20 mitotic cells at the mitotic metaphase stage, from the root tips of five individual bulbils.

### Genome, hi-C, and RNA sequencing

For genome sequencing, two different libraries were constructed and sequenced to generate enough reads. The libraries with a short insert size of ~400 bp were built using NEBNext® ULtra™ DNA Library Prep Kit for Illumina (UK) and subsequently sequenced on the Hiseq4000 platform (LC Sciences, Houston, TX, USA). The libraries with a long insert size of ~20 kb were built using SQK-LSK109 ligation kit and subsequently sequenced on the PromethION platform (Oxford Science Park, OX4 4DQ, UK).

For Hi-C sequencing, DNA extracted from the crosslinked samples was digested with Hind III and labeled with biotin. The biotin-labeled samples were fragmented to a size of 300–700 bp and subjected to end-repair, adenylation tailing, and universal adapter ligation. The biotin-containing DNA fragments were caught and used to prepare Hi-C sequencing libraries, which were subsequently sequenced on the HiSeq 4000 platform (LC Sciences, Houston, TX, USA).

For RNA sequencing, an equal quantity of RNAs isolated from each tissue (roots, stems, leaves, flowers, fruit capsules, bulbils, and rhizomes) were mixed and used to construct libraries using RNA library preparation kits (New England Biolabs, #E7530). The libraries were subsequently sequenced on the HiSeq X Ten platform (LC Sciences, Houston, TX, USA).

### Estimation of genome size, repeat content, and heterozygosity

The abundance of 21-K-mers was determined using the data generated from the short-insert-size libraries. The frequency of 21-K-mers was further analysed to obtain the genome size, repeat content, and heterozygosity of *A. hybrid* according to the formula: G = total K-mer number/average K-mer depth [27].

### Genome assembly

For genome assembly, the ONT data were self-corrected using Canu (v1.7) to remove errors [28]. The corrected ONT data were used to preliminarily assemble genome-v1 using Wtdbg2 (v2.1) [29] and genome-v2 using Smartdenovo (v1.0.6) (http://github.com/ruanjue/smartdenovo) independently. After that, genome-v1 was polished three rounds by aligning the corrected ONT reads using Racon (v1.3.1) [30] and corrected three rounds using Pilon (v1.2.2) [31] with Illumina data to obtain genome-v4. Genome-v2 was filtered to remove redundant sequences and duplicates using Purge haplotigs (1.1.0) [32] (l = 5; m = 72; h = 14) to obtain genome-v3. Subsequently, genome-v3 and genome-v4 were merged using Quickmerge [33] and polished using Racon and Pilon with ONT and Illumina data in three rounds for each software to improve its quality and yield the final genome. The Illumina data were mapped to the final genome [34], and tested results of Benchmarking Universal Single-Copy Ortholog (BUSCO) [35], the mapped ratio, and LTR assembly index (LAI) [36] were used to assess the assembly quality of the final genome.

### Chromosome-scale assembly with hi-C data

To assemble *A. hybrid* genome at the chromosome-scale, Hi-C reads were firstly cleaned by removing the low-quality sequences and adapters. The clean sequences were aligned to the final genome using BWA [37]. Duplicates were further removed using HiC-Pro (2.7.8) [38], and the uniquely mapped read pairs were retained to further analyse the valid interaction pairs required for assembly. Secondly, the valid interaction and unique mapped-reads were preassembled to identify and correct possible error regions. Subsequently, the corrected contigs or scaffolds were clustered, oriented, and ordered to reassemble the genome of *A. hybrid* at chromosome-scale using LACHESIS [39]. Finally, a genome-wide Hi-C heatmap was generated with a resolution of 100 kb to estimate the quality of the chromosome-scale genome assembly and visualize the interaction matrix of all chromosomes.

### Genome annotation

To predict protein-coding genes of *A. hybrid* genome, we applied multiple strategies, including *ab initio*-based, transcriptome-based, and homology-based predictions. For transcriptome-based prediction, mixed RNA samples from seven tissues were sequenced, and high-quality clean sequences were yielded after removal of low-quality sequences and adapters. These sequences were mapped to the *A. hybrid* genome using Stringtie (v1.2.3) [40] and Hisat (v2.0.4) [41], and the genome-guided and de novo transcriptomes were assembled using Ttrinity [42]. Subsequently, protein-coding genes were predicted using EST-based PASA (V2.0.2) [43]. For homology-based prediction, protein data from *A. setaceus* (WHSE00000000) [44], *A. officinalis* (PRJNA317340) [45], and *O. sativa* [46] were aligned to *A. hybrid* genome sequences using TBLASTN [47], and used to identify gene structures using GeMoMa (v1.3.1) [48, 49]. For ab initio-based prediction, five predictors, including Genscan [50], GeneID (v1.4) [51], Augustus [52], GlimmerHMM (v3.0.4) [53], and SNAP (v2006-07-28) [54], were used to identify the gene structure models. In the end, all predicted data from the above three strategies were integrated to obtain consensus gene sets using EvidenceModeler (EVM) (v1.1.1) [55].

To annotate the functions of these predicted protein-coding genes, their sequences were searched against multiple functional databases, including KEGG [56], Nr [57], TrEMBL [58], KOG [59], and GO [60] using BLAST (v2.2.31) [61].

Additionally, the genome sequences were aligned to the noncoding RNAs (ncRNAs) library from the Rfam (13.0) database [62] using BLASTN to annotate four types of ncRNAs, including transfer RNAs (tRNAs), ribosomal RNAs (rRNAs), microRNAs (miRNAs), and small nuclear RNAs (snRNAs). These sequences were also screened using tRNAscan-SE (1.3.1) software [63] with default parameters for eukaryotes to identify tRNA and using RNAmmer software (v 1.2) [64] to identify rRNAs.

To predict pseudogene, the protein-coding gene sequences were aligned to the genome sequences to search for orthologous and paralogous fragments using BLAT [65]. The identified orthologous and paralogous fragments were further analysed using GenWise [66] with default parameters to obtain pseudogenes by identifying immature stop codons and frameshift mutations.

### Identification of repeat sequences

To construct a repeat library for *A. hybrid*, we utilized a structure-based *de novo* approach by combining LTR-FINDER (v1.07) [67] and RepeatScout [68]. The predicted repeats in the *de novo* repeat library were classified using PASTEClassifier software [69] and integrated with the Repbase database [70] to create a final repeat library. Subsequently, we mapped the repeat library to the genome sequences using RepeatMasker [71] to search for known and novel repeat sequences.

### Comparative and evolutional analysis of *A. hybrid*

To conduct a comparative and evolutional analysis of *A. hybrid*, we downloaded 13 publicly available genome assemblies, extracted protein sequences from *A. hybrid* and the 13 genomes, were identified and clustered using Orthofinder v2.4 [72]. We annotated the resulting orthologous and paralogous groups using PANTHER V15 database [73]. We further classified and enriched the species-specific orthogroup of *A. hybrid* to the KEGG and GO databases using clusterProfile v3.14.0 [74].

Single-copy orthologous genes were isolated from 14 species and aligned reciprocally all-to-all using MAFFT v7.205 [75]. We converted protein alignments to codon alignments using PAL2NAL V14 [76] and eliminated the poorly sequenced and aligned regions from the codon alignment. The remaining sequences were concatenated to create super-genes using Gblocks v0.91b [77]. The optimal substitution model was determined using the ModelFinder [78] program in IQ-TREE (v1.6.11) [79]. We constructed the phylogenetic tree of 14 genomes based on the optimal substitution model using IQ-TREE (v1.6.11) [79] with the maximum-likelihood method with parameters setting as 1000 bootstrap replications. We further calculated the divergence times of *A. hybrid* from other species with *A. trichopoda* as the outgroup species using the MCMCTREE program in the PAML v4.9i package [80]. The calculation results were assessed and calibrated using the approximate likelihood calculation method based on three fossil records from the TimeTree (http://www.timetree.org/) for *A. trichopoda vs. S. lycopersicum* (168–194 Mya), *S. lycopersicum vs. S. oleracea* (107–116 Mya), and *A. thaliana vs. D. zibethinus* (81–94 Mya). The resulting phylogenetic tree with divergence times was visualized using MCMCTreeR v1.1 [81].

### Expanded and contracted gene families

We used CAFE v4.2 [82] to identify the expanded and contracted gene families in *A. hybrid* using the birth and death parameters according to the gene family classifying results and estimated divergence times. Gene families with both Viterbi and family-wide *P* values less than 0.05 were assumed to have undergone expansion and contraction. Furthermore, we annotated these gene families’ expansions and contractions based on PANTHER v15 [73] database and classified and enriched them based on the GO and KEGG database using the clusterProfiler v3.14.0 [74].

### Determination of positively selected codons

Single-copy orthologous genes were isolated from seven species (including *M. schizocarpa*, *P. dactylifera*, *O. sativa*, *P. equestris*, *A. hybrid*, *A. officinalis*, and *A. setaceus*) and aligned using MAFFT v7.205 [75]. Protein alignments were then converted into codon alignments using the PAL2NAL v14 [76] program. To identify positively selected codon sites, likelihood ratio tests were conducted using CodeML based on the Branch-site model in PAML [80], with a significance threshold of *P* < 0.01. Codon sites that had a probability of more than 0.95 based on Bayes empirical method were considered to be positively selected.

### Identification of synteny blocks and whole-genome duplications

Gene sequences were aligned using Diamond v0.929.130 [83] to identify pairwise genes with similar sequences (C score > 0.5, e < 1e-5) within and between genomes. To identify synteny blocks in chromosomes, the gff3 data were analysed using MCScanX [84] and visualized using JCVI v0.9.13 [85] and VGSC [86].

For WGD analysis, the substitution rate (Ks) of each gene was calculated using Wgd v1.1.1 [87], and the four-fold synonymous third codon transversion (4DTV) rate of each pairwise gene was estimated based on the published parameters (https://github.com/JinfengChen/Scripts) and further corrected using the HKY substitution model.

### Determination of LTR insertion times

LTR sequences (score >6) were searched in *A. hybrid* genome using LTR_FINDER v1.07 [67]. The flanking sequences at each side of non-redundant LTRs were isolated and aligned using MAFFT with parameters seting as —localpair —maxiterate 1000. The distance K was assessed based on the Kimura model in EMBOSS v6.6.0 [88] using the formula: T = K/(2 × r), where T is the divergence times and r is the neutral substitution rate (r = 7 × 10^−9^) [89].

### Constructing co-expression network

The co-expression network was generated using R package weighted gene co-expression network analysis (WGCNA) [90] and visualized using Cytoscape [91] and GraphPad Prism [92].

### Yeast one-hybrid assay

For yeast one-hybrid assay, we first constructed a normalized cDNA library by combining the Duplex-Specific Nuclease (DSN) and Switching Mechanism at the 5′ end of RNA Transcript (SMART) technology. The promotor sequences of *PEPK3* were cloned in the *pHIS2.1* vector (Clontech). A yeast library screening was performed following the methods described by Chen [93].

### Phylogenetic analysis

The one-step, build a maximum likelihood (ML) tree plugin of TBtools [94] was utilized in this study to phylogenetic analysis. This plugin integrates Muscle [95] for aligning protein or nucleotide sequences, TrimAI program [96] for removing poorly aligned regions or columns from the multiple sequence alignment (MSA), and IQ-TREE [79] for ML tree construction. These tools were employed in accordance with established academic conventions to perform a comprehensive phylogenetic analysis in this study. The phylogenetic tree is visualized using iTOL (https://itol.embl.de/).

### Gene gain and loss analysis

The Gene Gain & Lost Analysis plugin of TBtools [94] was utilized in this study to investigate the acquisition and loss of gene family members. This plugin integrates IQtree [79] for ML tree construction and incorporates Notung [97, 98] software for member acquisition and loss analysis. These tools were employed in accordance with established academic conventions to analyse the acquisition and loss of gene family members in this study.

## Supplementary Material

Web_Material_uhad269

## References

[1] Good-Avila SV , Souza V, Gaut BS. et al. Timing and rate of speciation in *Agave* (Agavaceae). Proc Natl Acad Sci U S A. 2006;103:9124–9 16757559 10.1073/pnas.0603312103 PMC1482577

[2] The Angiosperm Phylogeny Group and others . An update of the Angiosperm Phylogeny Group classification for the orders and families of flowering plants: APGIV. Bot J Linn Soc. 2016;181:1–20

[3] Davis SC , Simpson J, Gil-Vega KC. et al. Undervalued potential of crassulacean acid metabolism for current and future agricultural production. J Exp Bot. 2019;70:6521–37 31087091 10.1093/jxb/erz223 PMC6883259

[4] Stewart JR . *Agave* as a model CAM crop system for a warming and drying world. Front Plant Sci. 2015;6:684 26442005 10.3389/fpls.2015.00684 PMC4585221

[5] Trejo L , Limones V, Peña G. et al. Genetic variation and relationships among agaves related to the production of tequila and mezcal in Jalisco. Ind Crop Prod. 2018;125:140–9

[6] Yang XH , Cushman JC, Borland AM. et al. A roadmap for research on crassulacean acid metabolism (CAM) to enhance sustainable food and bioenergy production in a hotter, drier world. New Phytol. 2015;207:491–504 26153373 10.1111/nph.13393

[7] Silvera K , Neubig KM, Whitten WM. et al. Evolution along the crassulacean acid metabolism continuum. Funct Plant Biol. 2010;37:995–1010

[8] Wickell D , Kuo LY, Yang HP. et al. Underwater CAM photosynthesis elucidated by *Isoetes* genome. Nat Commun. 2021;12:6348 34732722 10.1038/s41467-021-26644-7 PMC8566536

[9] Cai J , Liu X, Vanneste K. et al. The genome sequence of the orchid *Phalaenopsis equestris*. Nat Genet. 2015;47:65–72 25420146 10.1038/ng.3149

[10] West-Eberhard MJ , Smith JAC, Winter K. Photosynthesis, reorganized. Science. 2011;332:311–2 21493847 10.1126/science.1205336

[11] Heyduk K , Ray JN, Ayyampalayam S. et al. Shifts in gene expression profiles are associated with weak and strong Crassulacean acid metabolism. Am J Bot. 2018;105:587–601 29746718 10.1002/ajb2.1017

[12] Ming R , VanBuren R, Wai CM. et al. The pineapple genome and the evolution of CAM photosynthesis. Nat Genet. 2015;47:1435–42 26523774 10.1038/ng.3435 PMC4867222

[13] Yang XH , Hu R, Yin H. et al. The *Kalanchoë* genome provides insights into convergent evolution and building blocks of crassulacean acid metabolism. Nat Commun. 2017;8:1899 29196618 10.1038/s41467-017-01491-7 PMC5711932

[14] Yin HF , Guo HB, Weston DJ. et al. Diel rewiring and positive selection of ancient plant proteins enabled evolution of CAM photosynthesis in *Agave*. BMC Genomics. 2018;19:588 30081833 10.1186/s12864-018-4964-7 PMC6090859

[15] Robert ML , Lim KY, Hanson L. et al. Wild and agronomically important *Agave* species (Asparagaceae) show proportional increases in chromosome number, genome size, and genetic markers with increasing ploidy. Bot J Lin Soc. 2008;158:215–22

[16] Bousios A , Saldana-Oyarzabal I, Valenzuela-Zapata AG. et al. Isolation and characterization of Ty1-copia retrotransposon sequences in the blue agave (*Agave tequilana* Weber var. Azul) and their development as SSAP markers for phylogenetic analysis. Plant Sci. 2007;172:291–8

[17] Sandoval S d CD , Juárez MJA, Simpson J. *Agave tequilana* MADS genes show novel expression patterns in meristems, developing bulbils and floral organs. Sex Plant Reprod. 2012;25:11–26 22012076 10.1007/s00497-011-0176-x

[18] Sun XD , Zhu S, Li N. et al. A chromosome-level genome assembly of garlic (*Allium sativum*) provides insights into genome evolution and allicin biosynthesis. Mol Plant. 2020;13:1328–39 32730994 10.1016/j.molp.2020.07.019

[19] Cheng H , Song X, Hu Y. et al. Chromosome-level wild *Hevea brasiliensis* genome provides new tools for genomic-assisted breeding and valuable loci to elevate rubber yield. Plant Biotechnol J. 2023;21:1058–72 36710373 10.1111/pbi.14018 PMC10106855

[20] Castorena-Sánchez I , Escobedo RM, Quiroz A. New cytotaxonomical determinants recognized in six taxa of Agave in the sections Rigidae and Sisalanae. Can J Bot. 1991;69:1257–64

[21] Ou CQ , Wang F, Wang J. et al. A de novo genome assembly of the dwarfing pear rootstock Zhongai 1. Sci Data. 2019;6:281 31767847 10.1038/s41597-019-0291-3 PMC6877535

[22] Wu HL , Ma T, Kang M. et al. A high-quality *Actinidia chinensis* (kiwifruit) genome. Hortic Res. 2019;6:117 31645971 10.1038/s41438-019-0202-y PMC6804796

[23] Trapnell C , Pachter L, Salzberg SL. TopHat: discovering splice junctions with RNA-Seq. Bioinformatics. 2009;25:1105–11 19289445 10.1093/bioinformatics/btp120 PMC2672628

[24] Deng H , Zhang LS, Zhang GQ. et al. Evolutionary history of PEPC genes in green plants: implications for the evolution of CAM in orchids. Mol Phylogenet Evol. 2016;94:559–64 26493226 10.1016/j.ympev.2015.10.007

[25] Zhang LS , Chen F, Zhang GQ. et al. Origin and mechanism of crassulacean acid metabolism in orchids as implied by comparative transcriptomics and genomics of the carbon fixation pathway. Plant J. 2016;86:175–85 26959080 10.1111/tpj.13159

[26] Heyduk K , Mckain MR, Lalani F. et al. Evolution of a CAM anatomy predates the origins of Crassulacean acid metabolism in the Agavoideae (Asparagaceae). Mol Phylogenet Evol. 2016;105:102–13 27591171 10.1016/j.ympev.2016.08.018

[27] Liu B , Shi Y, Yuan J. et al. Estimation of genomic characteristics by analyzing k-mer frequency in de novo genome projects. arXiv. 2013. preprint: not peer reviewed

[28] Koren S , Walenz BP, Berlin K. et al. Canu: scalable and accurate long-read assembly via adaptive k-mer weighting and repeat separation. Genome Res. 2017;27:722–36 28298431 10.1101/gr.215087.116 PMC5411767

[29] Ruan J , Li H. Fast and accurate long-read assembly with Wtdbg2. Nat Methods. 2019;17:155–8 31819265 10.1038/s41592-019-0669-3 PMC7004874

[30] Vaser R , Sovic I, Nagarajan N. et al. Fast and accurate de novo genome assembly from long uncorrected reads. Genome Res. 2017;27:737–46 28100585 10.1101/gr.214270.116 PMC5411768

[31] Walker BJ , Abeel T, Shea T. et al. Pilon: an integrated tool for comprehensive microbial variant detection and genome assembly improvement. PLoS One. 2014;9:e112963 25409509 10.1371/journal.pone.0112963 PMC4237348

[32] Roach MJ , Schmidt SA, Borneman AR. Purge Haplotigs: allelic contig reassignment for third-gen diploid genome assemblies. BMC Bioinformatics. 2018;19:460 30497373 10.1186/s12859-018-2485-7 PMC6267036

[33] Chakraborty M , Baldwin-Brown JG, Long AD. et al. Contiguous and accurate de novo assembly of metazoan genomes with modest long read coverage. Nucl Acids Res. 2016;44:e147 27458204 10.1093/nar/gkw654 PMC5100563

[34] Li H . Aligning sequence reads, clone sequences and assembly contigs with BWA-MEM. arXiv. 2013. preprint: not peer reviewed

[35] Simão FA , Waterhouse RM, Ioannidis P. et al. BUSCO: assessing genome assembly and annotation completeness with single-copy orthologs. Bioinformatics. 2015;31:3210–2 26059717 10.1093/bioinformatics/btv351

[36] Ou SJ , Chen JF, Jiang N. Assessing genome assembly quality using the LTR Assembly Index (LAI). Nucleic Acids Res. 2018;46:e126 30107434 10.1093/nar/gky730 PMC6265445

[37] Li H , Durbin R. Fast and accurate short read alignment with burrows–wheeler transform. Bioinformatics. 2009;25:1754–60 19451168 10.1093/bioinformatics/btp324 PMC2705234

[38] Servant N , Varoquaux N, Lajoie BR. et al. HiC-pro: an optimized and flexible pipeline for Hi-C data processing. Genome Biol. 2015;16:259 26619908 10.1186/s13059-015-0831-x PMC4665391

[39] Burton JN , Adey A, Patwardhan RP. et al. Chromosome-scale scaffolding of de novo genome assemblies based on chromatin interactions. Nat Biotechnol. 2013;31:1119–25 24185095 10.1038/nbt.2727 PMC4117202

[40] Pertea M , Pertea GM, Antonescu CM. et al. StringTie enables improved reconstruction of a transcriptome from RNA-seq reads. Nat Biotechnol. 2015;33:290–5 25690850 10.1038/nbt.3122 PMC4643835

[41] Kim D , Langmead B, Salzberg SL. HISAT: a fast spliced aligner with low memory requirements. Nat Methods. 2015;12:357–60 25751142 10.1038/nmeth.3317 PMC4655817

[42] Grabherr MG , Haas BJ, Yassour M. et al. Trinity: reconstructing a full-length transcriptome without a genome from RNA-Seq data. Nat Biotechnol. 2011;29:644–52 21572440 10.1038/nbt.1883 PMC3571712

[43] Campbell MA , Haas BJ, Hamilton JP. et al. Comprehensive analysis of alternative splicing in rice and comparative analyses with Arabidopsis. BMC Genomics. 2006;7:327 17194304 10.1186/1471-2164-7-327 PMC1769492

[44] Li SF , Wang J, Dong R. et al. Chromosome-level genome assembly, annotation and evolutionary analysis of the ornamental plant *Asparagus setaceus*. Hortic Res. 2020;7:48 32257234 10.1038/s41438-020-0271-y PMC7109074

[45] Harkess A , Zhou J, Xu C. et al. The asparagus genome sheds light on the origin and evolution of a young Y chromosome. Nat Commun. 2017;8:1279 29093472 10.1038/s41467-017-01064-8 PMC5665984

[46] Ouyang S , Zhu W, Hamilton J. et al. The TIGR rice genome annotation resource: improvements and new features. Nucleic Acids Res. 2006;35:D883–7 17145706 10.1093/nar/gkl976 PMC1751532

[47] Altschul SF , Madden TL, Schäffer AA. et al. Gapped BLAST and PSI-BLAST: a new generation of protein database search programs. Nucleic Acids Res. 1997;25:3389–402 9254694 10.1093/nar/25.17.3389 PMC146917

[48] Keilwagen J , Wenk M, Erickson JL. et al. Using intron position conservation for homology-based gene prediction. Nucleic Acids Res. 2016;44:e89 26893356 10.1093/nar/gkw092 PMC4872089

[49] Keilwagen J , Hartung F, Paulini M. et al. Combining RNA-seq data and homology-based gene prediction for plants, animals and fungi. BMC Bioinformatics. 2018;19:189 29843602 10.1186/s12859-018-2203-5 PMC5975413

[50] Burge C , Karlin S. Prediction of complete gene structures in human genomic DNA. J Mol Biol. 1997;268:78–94 9149143 10.1006/jmbi.1997.0951

[51] Alioto T , Blanco E, Parra G. et al. Using geneid to identify Genes. Curr Protoc Bioinformatics. 2018;64:e56 30332532 10.1002/cpbi.56

[52] Stanke M , Waack S. Gene prediction with a hidden Markov model and a new intron submodel. Bioinformatics. 2003;19:ii215-225 14534192 10.1093/bioinformatics/btg1080

[53] Majoros WH , Pertea M, Salzberg SL. TigrScan and GlimmerHMM: two open source ab initio eukaryotic gene-finders. Bioinformatics. 2004;20:2878–9 15145805 10.1093/bioinformatics/bth315

[54] Korf I . Gene finding in novel genomes. BMC Bioinformatics. 2004;5:59 15144565 10.1186/1471-2105-5-59 PMC421630

[55] Haas BJ , Salzberg SL, Zhu W. et al. Automated eukaryotic gene structure annotation using EVidenceModeler and the program to assemble spliced alignments. Genome Biol. 2008;9:R7 18190707 10.1186/gb-2008-9-1-r7 PMC2395244

[56] Kanehisa M , Goto S. KEGG: Kyoto Encyclopedia of Genes and Genomes. Nucleic Acids Res. 2000;28:27–30 10592173 10.1093/nar/28.1.27 PMC102409

[57] Koonin EV , Fedorova ND, Jackson JD. et al. A comprehensive evolutionary classification of proteins encoded in complete eukaryotic genomes. Genome Biol. 2004;5:R7 14759257 10.1186/gb-2004-5-2-r7 PMC395751

[58] Boeckmann B , Bairoch A, Apweiler R. et al. The SWISS-PROT protein knowledgebase and its supplement TrEMBL in 2003. Nucleic Acids Res. 2003;31:365–70 12520024 10.1093/nar/gkg095 PMC165542

[59] Marchler-Bauer A , Lu S, Anderson JB. et al. CDD: a Conserved Domain Database for the functional annotation of proteins. Nucleic Acids Res. 2011;39:D225–9 21109532 10.1093/nar/gkq1189 PMC3013737

[60] Dimmer EC , Huntley RP, Alam-Faruque Y. et al. The UniProt-GO annotation database in 2011. Nucleic Acids Res. 2012;40:D565–70 22123736 10.1093/nar/gkr1048 PMC3245010

[61] Altschul SF , Gish W, Miller W. et al. Basic local alignment search tool. J Mol Biol. 1990;215:403–10 2231712 10.1016/S0022-2836(05)80360-2

[62] Griffiths-Jones S , Moxon S, Marshall M. et al. Rfam: annotating non-coding RNAs in complete genomes. Nucleic Acids Res. 2005;33:D121–4 15608160 10.1093/nar/gki081 PMC540035

[63] Lowe TM , Eddy SR. tRNAscan-SE: a program for improved detection of transfer RNA genes in genomic sequence. Nucleic Acids Res. 1997;25:955–64 9023104 10.1093/nar/25.5.955 PMC146525

[64] Lagesen K , Hallin P, Rødland EA. et al. RNAmmer: consistent and rapid annotation of ribosomal RNA genes. Nucleic Acids Res. 2007;35:3100–8 17452365 10.1093/nar/gkm160 PMC1888812

[65] Kent WJ . BLAT--the BLAST-like alignment tool. Genome Res. 2002;12:656–64 11932250 10.1101/gr.229202 PMC187518

[66] Birney E , Clamp M, Durbin R. GeneWise and Genomewise. Genome Res. 2004;14:988–95 15123596 10.1101/gr.1865504 PMC479130

[67] Xu Z , Wang H. LTR_FINDER: an efficient tool for the prediction of full-length LTR retrotransposons. Nucleic Acids Res. 2007;35:W265–8 17485477 10.1093/nar/gkm286 PMC1933203

[68] Price AL , Jones NC, Pevzner PA. De novo identification of repeat families in large genomes. Bioinformatics. 2005;21:i351–8 15961478 10.1093/bioinformatics/bti1018

[69] Hoede C , Arnoux S, Moisset M. et al. PASTEC: an automatic transposable element classification tool. PLoS One. 2014;9:e91929 24786468 10.1371/journal.pone.0091929 PMC4008368

[70] Jurka J , Kapitonov VV, Pavlicek A. et al. Repbase update, a database of eukaryotic repetitive elements. Cytogenet Genome Res. 2005;110:462–7 16093699 10.1159/000084979

[71] Tarailo-Graovac M , Chen NS. Using RepeatMasker to identify repetitive elements in genomic sequences. Curr Protoc Bioinformatics. 2009;4:4.10.11–14.10.14 10.1002/0471250953.bi0410s25 19274634

[72] Emms DM , Kelly S. OrthoFinder: phylogenetic orthology inference for comparative genomics. Genome Biol. 2019;20:238 31727128 10.1186/s13059-019-1832-y PMC6857279

[73] Mi HY , Muruganujan A, Ebert D. et al. PANTHER version 14: more genomes, a new PANTHER GO-slim and improvements in enrichment analysis tools. Nucleic Acids Res. 2019;47:D419–26 30407594 10.1093/nar/gky1038 PMC6323939

[74] Yu GC , Wang LG, Han YY. et al. ClusterProfiler: an R package for comparing biological themes among gene clusters. OMICS. 2012;16:284–7 22455463 10.1089/omi.2011.0118 PMC3339379

[75] Katoh K , Asimenos G, Toh H. Multiple alignment of DNA sequences with MAFFT. Methods Mol Biol. 2009;537:39–64 19378139 10.1007/978-1-59745-251-9_3

[76] Suyama M , Torrents D, Bork P. PAL2NAL: robust conversion of protein sequence alignments into the corresponding codon alignments. Nucleic Acids Res. 2006;34:W609–12 16845082 10.1093/nar/gkl315 PMC1538804

[77] Talavera G , Castresana J. Improvement of phylogenies after removing divergent and ambiguously aligned blocks from protein sequence alignments. Syst Biol. 2007;56:564–77 17654362 10.1080/10635150701472164

[78] Kalyaanamoorthy S , Minh BQ, Wong TKF. et al. ModelFinder: fast model selection for accurate phylogenetic estimates. Nat Methods. 2017;14:587–9 28481363 10.1038/nmeth.4285 PMC5453245

[79] Nguyen LT , Schmidt HA, Von Haeseler A. et al. IQ-TREE: a fast and effective stochastic algorithm for estimating maximum-likelihood phylogenies. Mol Biol Evol. 2015;32:268–74 25371430 10.1093/molbev/msu300 PMC4271533

[80] Yang ZH . PAML: a program package for phylogenetic analysis by maximum likelihood. Bioinformatics. 1997;13:555–6 10.1093/bioinformatics/13.5.555 9367129

[81] Puttick MN . MCMCtreeR: functions to prepare MCMCtree analyses and visualize posterior ages on trees. Bioinformatics. 2019;35:5321–2 31292621 10.1093/bioinformatics/btz554

[82] Han MV , Thomas GW, Lugo-Martinez J. et al. Estimating gene gain and loss rates in the presence of error in genome assembly and annotation using CAFE 3. Mol Biol Evol. 2013;30:1987–97 23709260 10.1093/molbev/mst100

[83] Buchfink B , Xie C, Huson DH. Fast and sensitive protein alignment using DIAMOND. Nat Methods. 2015;12:59–60 25402007 10.1038/nmeth.3176

[84] Wang YP , Tang H, DeBarry JD. et al. MCScanX: a toolkit for detection and evolutionary analysis of gene synteny and collinearity. Nucleic Acids Res. 2012;40:e49 22217600 10.1093/nar/gkr1293 PMC3326336

[85] Tang HB , Krishnakumar VJ, Li JP. jcvi: JCVI utility libraries. Zenodo. 2015

[86] Xu YQ , Bi C, Wu G. et al. VGSC: a web-based vector graph toolkit of genome synteny and collinearity. Biomed Res Int. 2016;2016:7823429 27006949 10.1155/2016/7823429 PMC4783527

[87] Zwaenepoel A , Van de Peer Y. Wgd-simple command line tools for the analysis of ancient whole-genome duplications. Bioinformatics. 2019;35:2153–5 30398564 10.1093/bioinformatics/bty915 PMC6581438

[88] Rice P , Longden I, Bleasby A. EMBOSS: the European molecular biology open software suite. Trends Genet. 2000;16:276–7 10827456 10.1016/s0168-9525(00)02024-2

[89] Ossowski S , Schneeberger K, Lucas-Lledó JI. et al. The rate and molecular spectrum of spontaneous mutations in *Arabidopsis thaliana*. Science. 2010;327:92–4 20044577 10.1126/science.1180677 PMC3878865

[90] Langfelder P , Horvath S. WGCNA: an R package for weighted gene co-expression network analysis. BMC Bioinformatics. 2008;9:559 19114008 10.1186/1471-2105-9-559 PMC2631488

[91] Shannon P , Markiel A, Ozier O. et al. Cytoscape: a software environment for integrated models of biomolecular interaction networks. Genome Res. 2003;13:2498–504 14597658 10.1101/gr.1239303 PMC403769

[92] Prism 9 user guide, https://www.graphpad.com/guides/prism/latest/user-guide/index.htm.

[93] Chen X , Zhu Q, Nie Y. et al. Determination of conifer age biomarker DAL1 interactome using Y2H-seq. For Res. 2021;1:12 10.48130/FR-2021-0012 PMC11524280 39524519

[94] Chen CJ , Chen H, Zhang Y. et al. TBtools: an integrative toolkit developed for interactive analyses of big biological data. Mol Plant. 2020;13:1194–202 32585190 10.1016/j.molp.2020.06.009

[95] Edgar RC . MUSCLE: multiple sequence alignment with high accuracy and high throughput. Nucleic Acids Res. 2004;32:1792–7 15034147 10.1093/nar/gkh340 PMC390337

[96] Capella-Gutiérrez S , Silla-Martínez JM, Gabaldón T. TrimAl: a tool for automated alignment trimming in large-scale phylogenetic analyses. Bioinformatics. 2009;25:1972–3 19505945 10.1093/bioinformatics/btp348 PMC2712344

[97] Stolzer M , Lai H, Xu M. et al. Inferring duplications, losses, transfers and incomplete lineage sorting with nonbinary species trees. Bioinformatics. 2012;28:i409–15 22962460 10.1093/bioinformatics/bts386 PMC3436813

[98] Chen K , Durand D, Farach-Colton M. NOTUNG: a program for dating gene duplications and optimizing gene family trees. J Comput Biol. 2000;7:429–47 11108472 10.1089/106652700750050871

