# Supplementary material for: A chromosome-level genome assembly of Agave hybrid NO.11648 provides insights into the CAM photosynthesis
Source: Hortic Res. 2023 Dec 19;11(2):uhad269. doi: 10.1093/hr/uhad269 (PMC10848310; doi:10.1093/hr/uhad269)
Supplement: Web_Material_uhad269 [file web_material_uhad269.pdf]

Supplementary Information

A chromosome-level genome assembly of *Agave hybrid* NO.11648 provides insights into the CAM photosynthesis

Ziping YANG[1,*], Qian YANG[1,*], Qi LIU[2], Xiaolong LI[3], Luli WANG[1], Yanmei ZHANG[1], Zhi KE[1], Zhiwei LU[1], Huibang SHEN[1], Junfeng LI[1], Wenzhao ZHOU[1]

[1]South Subtropical Crops Research Institute, Chinese Academy of Tropical Agricultural Sciences/Zhanjiang Key Laboratory of Tropical Crop Genetic Improvement, 524091 Zhanjiang, Guangdong, China. [2]Wuhan Onemore-tech Co., Ltd, 430076 Wuhan, Hubei, China. [3]Biomarker Technologies Corporation, 101300 Beijing, China. [*]These authors contributed equally to this work and should be considered co-first authors. Correspondence should be addressed to Ziping YANG (yangziping302@163.com or yangziping302@catas.cn) and Wenzhao ZHOU (zwenzhao@catas.cn).

Content

Supplementary Tables 5

Table S1. The data statistics of flow cytometry analysis 5
Table S2. Sequencing data used for *A. hybrid* genome assembly and annotation 5
Table S3. The data statistics of the 21-mer analysis and heterozygosity in *A. hybrid* genome 5
Table S4. Sequencing statistics from the Nanopore..5
Table S5. Statistics of data generated using the Illumina platform..6
Table S6. Size distribution of the Nanopore subreads 6
Table S7. Summary statistics of the assembled genome by WTDBG 6
Table S8. Summary statistics of the assembled genome by Smartdenovo 6
Table S9. Summary statistics of the assembled genome by Quickmerge 7
Table S10. Statistics of data generated by Hi-C..7
Table S11. Mapping statistics of Hi-C clean read pairs to the merge-genomen 7
Table S12. Summary statistics of different types of Hi-C read pairs.......................................8
Table S13. Statistics of the chromosome assemblies using Hi-C data.....................................9
Table S14. Statistics of the *A. hybrid* genome assembly based on the Hi-C data using LACHESIS...10
Table S15. BUSCO assessment of the *A. hybrid* genome assembly......................................10
Table S16. Mapping statistics of Illumina data to the final genome......................................10
Table S17. Evaluation of the genome assembly with the RNA-Seq data 10
Table S18. Evaluation of the genome assembly with LTR assembly index 10
Table S19. Statistics of the repeat sequences in the *A. hybrid* genome 11
Table S20. Statistics of gene prediction 12
Table S21. Statistics of all predicted genes...12
Table S22. Statistics of function annotation of the *A. hybrid* protein-coding genes 12
Table S23. Mapping statistics of transcriptome data to the final genome 12
Table S24. Statistics of the noncoding RNA in the *A. hybrid* genome 13
Table S25. Statistics of the pseudogene in the *A. hybrid* genome..13
Table S26. The number of each CAM-related genes in each taxon.......................................13
Table S27. Expression of CAM gene families in *A. hybrid* in leaf and other tissues 14
Table S28. Expression of CAM gene families in *A. hybrid* at night and in the daytime 15
Table S29. Information on taxa included in this study..17

Supplementary Figures..19

Figure S1. Cytological analysis of *A. hybrid* chromosomes 19
Figure S2. Distribution of 21-k-mers from the Illumina data 19
Figure S3. An overview of genome assembly workflow 20
Figure S4. The genome-wide Hi-C heatmap of *A. hybrid*..21
Figure S5. An overview of genome annotation workflow 22
Figure S6. Distribution map of integrated gene numbers derived from three prediction methods 23

Figure S7. Venn diagram of orthologous gene families among *A. hybrid*, *A. officinalis*, *A. setaceus*, *P. dactylifera*, and *M. schizocarpa* 23
Figure S8. Distribution of genes and gene families of 14 plant species 24
Figure S9. Enrichment analysis of unique gene families from *A. hybrid* genome 24
Figure S10. Phylogenetic tree constructed with orthologs 25
Figure S11. Divergence time estimated for *A. hybrid* 25
Figure S12. Enrichment analysis of significantly expanded gene families in *A. hybrid* genome 26
Figure S13. Enrichment analysis of positive selection gene families from *A. hybrid* genome 26
Figure S14. Ka/Ks distribution in *A. hybrid* and other representative plant species 27
Figure S15. Synteny analysis within A. hybrid genomes 27
Figure S16. Synteny analysis within A. hybrid genomes 28
Figure S17. Comparison of *A. hybrid* genome with *A. setaceus* genome 28
Figure S18. The synteny relationship between *A. hybrid* and *A. officinalis* (A) and between *A. hybrid* and *A. setaceus* (B) 29
Figure S19. Phylogenetic tree of *αCA* gene family 30
Figure S20. Phylogenetic tree of *βCA* gene family 31
Figure S21. Phylogenetic tree of *γCA* gene family 32
Figure S22. Phylogenetic tree of *NAD-MDH* gene family 33
Figure S23. Phylogenetic tree of *NAD-ME* gene family 34
Figure S24. Phylogenetic tree of *NADP-MDH* gene family 35
Figure S25. Phylogenetic tree of *NADP-ME* gene family 36
Figure S26. Phylogenetic tree of *PEPC* gene family 37
Figure S27. Phylogenetic tree of *PEPCK* gene family 38
Figure S28. Phylogenetic tree of *PPCK* gene family 39
Figure S29. Phylogenetic tree of *PPDK* gene family 40
Figure S30. Phylogenetic tree of *PPDK-R* gene family 41
Figure S31. Phylogenetic tree of *PEPCK* gene family of *A. hybrid* 42
Figure S32. Distribution of *PEPCK* genes on the 4 chromosomes of *A. hybrid* genome 43
Figure S33. Statistics of gene gain and loss in 21 species 43
Figure S34. Key genes of the CAM pathway and their diel expression profiles 44
Figure S35. A maximum-likelihood phylogeny of phosphoenolpyruvate carboxylase (PEPC) 45
Figure S36. Expression of gene families in *A. hybrid* in leaf and other tissues 46
Figure S37. Expression of gene families in *A. hybrid* in night and day 47
Figure S38. Clustering dendrogram of genes and merged module colors 48
Figure S39. Hierarchical cluster tree and the relationship between gene co-expression modules (heatmap) 49
Figure S40. Module-phenolics weight correlations 50
Figure S41. KEGG classification of co-expressed genes in 11 modules (bisque4, sienna3, pluml, lightcyanl1, lavenderblush, grey60, greenyellow, darkorange2, darkorange, corall, brown, and lightblue4) 51
Figure S42. Cytoscape representation of co-expressed genes involved in the CAM pathway in the greenyellow modules 52

Figure S43. Cytoscape representation of co-expressed genes involved in the CAM pathway in darkorange2 53
Figure S44. Cytoscape representation of co-expressed genes involved in the CAM pathway in sienna3 54
Figure S45. Cytoscape representation of co-expressed genes involved in the CAM pathway in grey60 55
Figure S46. Distribution of *YUCCA* genes on the 16 chromosomes of *A. hybrid* genome 56
Figure S47. Distribution of *SAUR* genes on the 17 chromosomes of *A. hybrid* genome 56
Figure S48. Distribution of *ERF* genes on the 28 chromosomes of *A. hybrid* genome 57
Figure S49. Distribution of *LOX* genes on the 16 chromosomes of *A. hybrid* genome 57
Figure S50. Distribution of *JAZ* genes on the 11 chromosomes of *A. hybrid* genome 58
Figure S51. Distribution of *SWEET* genes on the 17 chromosomes of the *A. hybrid* genome 58
Figure S52. Yeast one-hybrid assay for screening transcription factors 59

References 59

Supplementary Tables

Table S1. The data statistics of flow cytometry analysis.

Sample ID	Internal reference species	Internal reference fluorescence intensity	Sample fluorescence intensity	Ratio	Genome size（G）	Average（G）	Stdev
1	*Zea mays*	97.12	175.94	1.81	4.17	4.14	0.03
2	*Zea mays*	97.33	175.15	1.80	4.14		
3	*Zea mays*	97.48	174.44	1.79	4.12		

Table S2. Sequencing data used for *A. hybrid* genome assembly and annotation. The coverage was calculated using an estimated genome size of 4.25 Gb based on *k-mer* analysis.

Types	Sequencing Libraries	Sequencing Plantform	Insert Size (bp)	Clean data (Gbp)	Coverage (X)	Use of the Data
Genome	Illumina	Hiseq4000	350	488.22	114.87	Genome estimation and polishing
Genome	Nanopore	PromethION		410.91	96.68	Genome assembly
Genome	Hi-C	HiSeq 4000	350	478.02	112.47	Chromosome construction
Transcriptome	Illumina	HiSeq X ten	350	10.56	-	Annotation

Table S3. The data statistics of the 21-mer analysis and heterozygosity in *A. hybrid* genome.

kmer	Total ker Number	kmer Depth	Genome Size (bp)	Repeat (%)	GC content (%)	Heterozygosity (%)
21	377,139,903,527	86	4.25	80.29	38.97	0.42

Table S4. Sequencing statistics from the Nanopore.

Data Type	Sequencing Number	Sequencing Base (bp)	N50Length (bp)	N90Length (bp)	MeanLength (bp)	MaxLength (bp)	Mean Qual
Raw data	35,885,482	472,918,756,901	33,646	16,575	13,178	1,004,160	4.51
Clean data	14,610,915	410,908,581,397	34,576	18,956	28,123	964,668	7.74

Table S5. Statistics of data generated using the Illumina platform.

	Clean Data(bp)	Q20(%)	Q30(%)	N(%)	GC(%)
Before Filter	488221105200 (100.00%)	474943550026 (97.28%)	451286573875 (92.43%)	3069236 (0.00%)	195951625144 (40.14%)
After Filter	469294641313 (96.12%)	460132516971 (98.05%)	438924950210 (93.53%)	2912930 (0.00%)	187940114372 (40.05%)

Table S6. Size distribution of the Nanopore subreads.

Length (bp)	Reads Number	Total Length (bp)	Percent	AverageLength (bp)
2000～5000	1,194,778	4,046,002,129	0.98%	3,386.40
5000～10000	1,330,596	9,820,189,722	2.38%	7,380.29
10000～20000	2,162,939	32,626,373,707	7.94%	15,084.27
20000～30000	4,470,670	112,649,878,728	27.41%	25,197.53
30000～40000	2,533,092	86,782,978,563	21.11%	34,259.70
40000～50000	1,249,188	55,691,657,799	13.55%	44,582.28
50000～60000	766,318	41,812,004,158	10.17%	54,562.21
60000～70000	436,268	28,139,393,422	6.84%	64,500.24
70000～80000	233,566	17,393,203,063	4.23%	74,468.04
>=80000	233,500	21,946,900,106	5.34%	93,991
Total	14,610,915	410,908,581,397	100%	28,123

Table S7. Summary statistics of the assembled genome by WTDBG.

Contig number	Contig length (bp)	Contig N50 (bp)	Contig N90 (bp)	Contig max (bp)	GC content (%)	Gap toltal length (bp)
28,955	4,871,066,853	311,144	68,937	3,665,869	38.42	0

Table S8. Summary statistics of the assembled genome by Smartdenovo.

Contig number	Contig length (bp)	Contig N50 (bp)	Contig N90 (bp)	Contig max (bp)	GC content (%)	Gap toltal length (bp)
13,484	5,914,610,519	685,738	207,883	4,641,582	37.83	0

Table S9. Summary statistics of the assembled genome by Quickmerge.

Contig number	Contig length (bp)	Contig N50 (bp)	Contig N90 (bp)	Contig max (bp)	GC content (%)	Gap toltal length (bp)
7,279	4,875,385,898	1,062,458	328,773	9,469,737	38.36	0

Table S10. Statistics of data generated Hi-C data.

Library	ReadSum	BaseSum	GC (%)	N (%)	Q20 (%)	Q30 (%)
H01	277,604,389	82,757,657,778	41.87	0	98	94.26
H02	127,029,910	37,885,442,386	41.78	0	97.2	92.16
H03	302,457,281	90,372,416,412	43.03	0	97.98	94.13
H04	100,294,857	29,966,306,442	43.1	0	97.78	93.5
H05	20,980,230	6,284,567,140	39.72	0	97.08	91.77
H06	770,274,866	230,751,381,612	39.99	0	97.49	92.8
Total	1,598,641,533	478,017,771,770	-	-	-	-

Table S11. Mapping statistics of Hi-C clean read pairs to the merge-genome.

Library	Mapping Type	Number	Ratio（%）
H01	Total Read Pairs	277,604,389	100
	Mapped Reads	353,813,169	63.73
	Unique Mapped Read Pairs	58,574,736	21.1
H02	Total Read Pairs	127,029,910	100
	Mapped Reads	161,607,566	63.61
	Unique Mapped Read Pairs	26,905,008	21.18
H03	Total Read Pairs	302,457,281	100
	Mapped Reads	393,271,244	65.01
	Unique Mapped Read Pairs	63,581,938	21.02
H04	Total Read Pairs	100,294,857	100
	Mapped Reads	130,226,561	64.92
	Unique Mapped Read Pairs	21,055,576	20.99
H05	Total Read Pairs	20,980,230	100
	Mapped Reads	31,985,292	76.23
	Unique Mapped Read Pairs	4,417,909	21.06
H06	Total Read Pairs	770,274,866	100
	Mapped Reads	1,177,056,333	76.4
	Unique Mapped Read Pairs	160,563,561	20.84

Table S12. Summary statistics of different types of Hi-C read pairs.

Library	Mapping Type	Number	Ratio（%）
H01	Unique Paired Alignments	58,574,736	100
	Valid Interaction Pairs	13,771,082	23.51
	Dangling End Pairs	39,143,174	66.83
	Re-ligation Pairs	1,407,539	2.4
	Self-cycle Pairs	68,331	0.12
	Dumped Pairs	4,184,610	7.14
H02	Unique Paired Alignments	26,905,008	100
	Valid Interaction Pairs	6,224,293	23.13
	Dangling End Pairs	18,136,906	67.41
	Re-ligation Pairs	648,980	2.41
	Self-cycle Pairs	30,877	0.11
	Dumped Pairs	1,863,952	6.93
H03	Unique Paired Alignments	63,581,938	100
	Valid Interaction Pairs	14,739,014	23.18
	Dangling End Pairs	42,873,457	67.43
	Re-ligation Pairs	1,528,138	2.4
	Self-cycle Pairs	57,604	0.09
	Dumped Pairs	4,383,725	6.89
H04	Unique Paired Alignments	21,055,576	100
	Valid Interaction Pairs	4,848,154	23.03
	Dangling End Pairs	14,225,737	67.56
	Re-ligation Pairs	509,513	2.42
	Self-cycle Pairs	18,586	0.09
	Dumped Pairs	1,453,586	6.9
H05	Unique Paired Alignments	4,417,909	100
	Valid Interaction Pairs	1,811,355	41
	Dangling End Pairs	1,675,836	37.93
	Re-ligation Pairs	79,705	1.8
	Self-cycle Pairs	364,341	8.25
	Dumped Pairs	486,672	11.02
H06	Unique Paired Alignments	160,563,561	100
	Valid Interaction Pairs	67,487,513	42.03
	Dangling End Pairs	57,619,091	35.89
	Re-ligation Pairs	2,932,368	1.83
	Self-cycle Pairs	13,187,646	8.21
	Dumped Pairs	19,336,943	12.04

Table S13. Statistics of the chromosome assemblies using Hi-C data.

Lachesis Group	Clustered sequences		Ordered and oriented sequences		Gene number	Gap number	Gap base （N）	N (%)
	Number	Length (bp)	Number	Length (bp)				
Chr 01	753	584,219,279	656	567,083,952	4740	655	65500	0.01%
Chr 02	606	493,015,331	517	480,722,948	3673	516	51600	0.01%
Chr 03	633	506,806,792	537	492,125,811	3848	536	53600	0.01%
Chr 04	638	435,824,761	517	419,573,385	3853	516	51600	0.01%
Chr 05	677	458,594,586	528	438,074,831	4401	527	52700	0.01%
Chr 06	317	196,303,097	245	186,399,855	2728	244	24400	0.01%
Chr 07	227	183,459,067	178	173,251,141	1735	177	17700	0.01%
Chr 08	192	149,873,448	146	139,386,507	1408	145	14500	0.01%
Chr 09	293	148,851,573	191	132,903,991	2981	190	19000	0.01%
Chr 10	125	77,918,623	91	73,153,047	1100	90	9000	0.01%
Chr 11	102	54,079,761	64	48,880,695	698	63	6300	0.01%
Chr 12	176	109,785,879	135	102,397,193	1598	134	13400	0.01%
Chr 13	173	109,893,287	133	104,167,531	1303	132	13200	0.01%
Chr 14	182	112,072,332	123	101,443,392	1329	122	12200	0.01%
Chr 15	166	99,423,801	129	94,216,449	1447	128	12800	0.01%
Chr 16	160	100,810,371	127	95,365,090	1315	126	12600	0.01%
Chr 17	181	93,367,099	137	85,956,028	1504	136	13600	0.02%
Chr 18	143	87,962,552	105	82,670,778	1161	104	10400	0.01%
Chr 19	110	72,641,647	89	69,818,142	871	88	8800	0.01%
Chr 20	140	74,126,233	101	68,802,295	854	100	10000	0.01%
Chr 21	119	71,423,710	86	66,975,850	1006	85	8500	0.01%
Chr 22	115	69,203,615	84	63,713,717	852	83	8300	0.01%
Chr 23	112	66,193,799	80	60,026,272	870	79	7900	0.01%
Chr 24	103	64,298,821	79	61,291,038	752	78	7800	0.01%
Chr 25	193	97,307,222	123	85,551,391	1661	122	12200	0.01%
Chr 26	235	115,876,924	142	103,311,517	2288	141	14100	0.01%
Chr 27	96	57,877,754	64	50,370,507	467	63	6300	0.01%
Chr 28	61	43,536,143	42	40,205,524	418	41	4100	0.01%
Chr 29	45	36,406,158	30	34,437,724	438	29	2900	0.01%
Chr 30	83	51,151,697	35	36,998,176	272	34	3400	0.01%
Total of Chr 01-30 (Ratio %)	7156 (96.07)	4822305362 (98.91)	5514 (77.05)	4559274777 (94.55)	51571	5484	548400	0.37%

Sequence number and length was calculated with all clustered sequences > 1Kb.

The sequence length are the sum length of corresponding number contigs in which are without Ns.

Table S14. Statistics of the *A. hybrid* genome assembly based on the Hi-C data using LACHESIS.

Statistical level	Scaffold	Contig
Total number	1,965	7,449
Total Length(bp）	4,875,934,298	4,875,385,898
N50 Length(bp）	186,424,255	1,007,035
N90 Length(bp）	50,376,807	328,773
Maximum Length(bp）	567,149,452	7,500,000
Gap total length (bp)	548,400	-
GC content (%)	-	38.36

Table S15. BUSCO assessment of the *A. hybrid* genome assembly.

BUSCO benchmark	Number	Percent (%)
Complete BUSCOs(C)	1306	90. 69
Complete and single-copy BUSCOs(S)	1012	70. 28
Complete and duplicated BUSCOs(D)	294	20. 42
Fragmented BUSCOs(F)	39	2. 71
Missing BUSCOs(M)	95	6. 60
Total Lineage BUSCOs	1440	100

Table S16. Mapping statistics of Illumina data to the final genome.

Total reads	Mapped reads	Mapped (%)	Properly Mapped Reads	Properly Mapped (%)
2948066355.00	2921891310.00	99.11	2262663538.00	76.75

Table S17. Evaluation of the genome assembly with the RNA-Seq data.

	Unigene Number	Percent (%)	>50% Number	Percent (%)	>90% Number	Percent
All	287926	99.63%	287043	99.33%	276013	95.51%
>=500	139673	99.65%	139543	99.56%	135927	96.98%
>=1000	65957	99.66%	65944	99.64%	65128	98.41%

Table S18. Evaluation of the genome assembly with LTR Assembly Index.

**Chr**	**From**	**To**	**Intact**	**Total**	**Raw LAI**	**LAI**
Whole Genome	1	4875934298	0.0394	0.6215	6.34	10.95

Table S19. Statistics of the repeat sequences in the *A. hybrid* genome.

Type	Number	Length (bp)	Rate (%)
ClassI:Retroelement	3447840	3267690538	67.02
ClassI/DIRS	427	274242	0.01
ClassI/LINE	119972	41554602	0.85
ClassI/LTR/Caulimovirus	737	630819	0.01
ClassI/LTR/Copia	808420	894178492	18.34
ClassI/LTR/ERV	11831	850968	0.02
ClassI/LTR/Gypsy	538502	658445887	13.5
ClassI/LTR/Ngaro	1004	75002	0
ClassI/LTR/Pao	423	30413	0
ClassI/LTR/Unknown	1957342	1670528981	34.26
ClassI/SINE	9182	1121132	0.02
ClassII:DNA transposon	887759	482434508	9.89
ClassII/Academ	4	242	0
ClassII/CACTA	5606	369135	0.01
ClassII/Crypton	39	1549	0
ClassII/Dada	538	25655	0
ClassII/Ginger	66	2694	0
ClassII/Helitron	554986	328843663	6.74
ClassII/IS3EU	509	35954	0
ClassII/Kolobok	1846	202467	0
ClassII/Maverick	515	38023	0
ClassII/Merlin	271	11467	0
ClassII/Mutator	18866	11047652	0.23
ClassII/P	216	14684	0
ClassII/PIF-Harbinger	1100	62999	0
ClassII/PiggyBac	149	5517	0
ClassII/Tc1-Mariner	494	27663	0
ClassII/Unknown	281607	130518765	2.68
ClassII/Zisupton	297	15232	0
ClassII/hAT	20650	11211147	0.23
SSR	9,279	2,340,405	0.05
Unknown	61	3341	0
Total	4335660	3750128387	76.91

Table S20. Statistics of gene prediction.

Method	Software	Sepecies	Gene number
Ab initio	Genscan	-	79,289
	Augustus	-	67,571
	GlimmerHMM	-	174,625
	GeneID	-	170,836
	SNAP	-	144,917
Homology-based	GeMoMa	*Asparagus officinalis*	53,472
		Asparagus setaceus	52,323
		*Oryza_sativa*_Japonica.MSU_v7.0	46,750
RNAseq	TransDecoder	-	67,465
	GeneMarkS-T	-	74,389
	PASA	-	45,370
Integration	EVM	-	58,841

Table S21. Statistics of all predicted genes.

Type	Length (bp)	Average Length (bp)	Number	Average Number
Exon	76,125,799	1,293.75	329,056	5.59
CDS	63,963,396	1,087.05	319,992	5.44
Intron	469,484,563	7,978.87	270,215	4.59
Total Gene	545,610,362	9,272.62	58,841	-

Table S22. Statistics of function annotation of the *A. hybrid* protein-coding genes.

Annotation database	Annotated number	Percentage (%)
GO_Annotation	28,133	47.81
KEGG_Annotation	20,677	35.14
KOG_Annotation	30,302	51.5
TrEMBL_Annotation	53,085	90.22
nr_Annotation	53,768	91.38
All_Annotation	53,849	91.52

Table S23. Mapping statistics of transcriptome data to the final genome.

Type	Base Number (bp)	Mapped Length (bp)	Mapped Ratio (%)	Mapped Number	Mapped Ratio (%)
Exon	74438084	57449567	77.18%	7018007789	77.09%
Intro	469484563	46959472	10.00%	566490118	6.22%
Intergenic	4331463251	47920856	1.11%	1519741705	16.69%
Total	4875385898	-	-	9104239612	-

Table S24. Statistics of the noncoding RNA in the *A. hybrid* genome.

RNA Type	Number	Family
miRNA	291	23
rRNA	1,718	4
tRNA	1,263	24

Table S25. Statistics of the pseudogene in the *A. hybrid* genome.

Sotfware	Number	Total length (bp)	Average length (bp)
GeneWise	5,264	41,883,528	7,956.60

Table S26. The number of each CAM-related genes in each taxon.

Pho[a]	Species name	Gene name												
		αCA	βCA	γCA	NADP-MDH	NAD-MDH	NADP-ME	NAD-ME	PEPC	PEPCK	PEPC-R	PPCK	PPDK	PPDK-R
C3	*Amborella trichopoda*	5	2	2	2	4	1	2	2	1	2	3	1	1
C3	*Arabidopsis thaliana*	8	6	5	4	5	4	2	4	2	2	2	1	2
C3	*Asparagus officinalis*	9	2	3	4	3	4	2	4	2	2	1	1	1
C3	*Brachypodium distachyon*	6	3	3	3	5	5	2	3	0	2	1	1	1
C3	*Musa acuminata*	9	4	2	4	8	3	2	6	6	4	3	3	1
C3	*Oryza sativa*	8	2	4	3	7	4	2	6	2	2	1	2	1
C3	*Vitis vinifera*	5	6	2	3	6	2	2	3	2	3	0	1	1
C3	*Apostasia shenzhenica*	3	2	2	4	3	4	2	3	2	1	2	1	0
C3	*Asparagus setaceus*	3	2	4	1	6	3	2	4	4	0	1	1	1
C3/CAM	*Dendrobium catenatum*	4	4	2	3	4	3	2	2	2	4	2	1	1
C4	*Setaria italica*	9	4	3	4	7	4	2	6	3	3	1	2	1
C4	*Sorghum bicolor*	10	3	1	4	6	6	2	6	3	3	1	2	3
C4	*Zea mays*	9	5	1	4	8	6	2	6	4	3	2	2	2
CAM	*Ananas comosus*	3	3	3	3	5	2	2	3	2	2	1	1	1
CAM	*Kalanchoe fedtschenkoi*	3	6	5	4	8	5	6	4	4	2	1	1	2
CAM	*Kalanchoe laxiflora*	7	11	10	8	15	9	10	8	8	2	4	2	4
CAM	*Portulaca amilis*	7	6	4	1	7	3	1	4	1	2	1	2	2
CAM	*Hylocereus undatus*	5	7	4	5	5	6	3	3	2	1	1	2	2
CAM	*Phalaenopsis aphrodite*	9	2	2	2	2	3	2	2	2	1	2	1	1
CAM	*Phalaenopsis equestris*	6	3	2	2	3	3	2	2	2	0	2	1	1
CAM	*Agave hybrid* No.11648	12	7	8	4	13	7	3	4	12	0	3	2	2

[a]Photosynthetic types

Table S27. Expression of gene families in *A. hybrid* in leaf and other tissues.

Gene	Leaf	Others tissues	（Leaf +10）/(Others tissues+10)
PPDK1	1773.162727	10.62036992	86.47578747
PEPC4	6003.194865	67.59533508	77.49428311
PPDK2	690.9718534	3.09405675	53.53358908
βCA3	595.4164954	10.06987558	30.16543341
PEPCK5	1303.007675	44.844407	23.9405939
PEPCK12	619.80244	18.80158358	21.86693791
PEPCK3	511.5135364	18.47762933	18.31309518
βCA5	126.6542096	0.508225583	13.00449905
NAD-MDH10	166.2278743	8.787823585	9.379898289
NAD-MDH8	121.2483321	7.819984418	7.365232711
NADP-MDH4	340.6873083	45.09539025	6.365093463
NADP-MDH2	645.5169594	103.012786	5.800378724
NAD-ME3	192.3465538	36.52567642	4.34913728
NAD-ME2	125.0288264	52.70251575	2.153483393
NAD-MDH13	62.17828125	25.13669534	2.054213709
PPDK-R2	12.54670506	1.274295	1.99983281
NAD-MDH12	24.82863442	8.460707084	1.886635992
PPDK-R1	9.007554806	0.691392167	1.777837209
NADP-ME2	153.465677	82.82139708	1.761077533
NAD-ME1	40.52823756	19.58828775	1.707710767
NADP-ME4	27.65454114	14.03857175	1.56642173
βCA7	9.901583861	3.734152667	1.449058005
PEPC1	43.42675109	27.25673634	1.434015868
aCA6	5.874112417	1.089399083	1.431467323
aCA7	5.090963306	0.650316167	1.416949795
γCA5	22.45195958	14.99594625	1.298288901
βCA4	6.774823444	3.19191125	1.271599174
βCA2	8.516951305	4.571320833	1.270780564
γCA3	28.41602242	22.4311325	1.184541502
γCA8	15.65670942	12.1059835	1.160622843
PEPC3	30.73677719	25.47927625	1.148185124
γCA7	38.67429297	34.78890408	1.086748916
aCA9	7.404715583	6.071860333	1.082930988
βCA6	4.516376139	3.94813925	1.040739261
βCA1	1.257759667	1.061982	1.017698245
γCA2	42.77998847	41.99002917	1.015194439
aCA1	0.98133	1.045784	0.994164833
aCA4	0	0.062893584	0.99374995
γCA6	2.831608583	2.940716417	0.99156864
PEPCK7	0.030228222	0.163823917	0.986855765
aCA12	0	0.331597583	0.96790452
aCA2	0	0.438577417	0.957984944

aCA11	1.026521111	1.56756025	0.953227895
PEPCK8	0.259508167	0.766883167	0.952876335
PEPCK6	0.302171555	0.922918333	0.943170245
aCA8	0.026334111	1.1591245	0.898487521
NAD-MDH7	31.10598253	35.86792908	0.896181348
PEPCK10	1.358616472	2.695094167	0.89472487
PEPCK1	2.346108945	4.184369916	0.870402353
PPCK3	0.279815833	1.895204667	0.864198315
PEPCK9	2.182173056	4.1477275	0.861069246
PEPCK11	2.413510223	4.443373334	0.859460594
NAD-MDH1	0.330604472	2.104301	0.853465596
γCA1	14.33365397	18.52273525	0.853131853
NAD-MDH9	23.81752155	30.287874	0.839397024
γCA4	11.68123883	16.689452	0.812352342
PEPCK2	0.094368833	2.513687	0.806666239
NADP-MDH1	59.76589603	78.48511383	0.78844783
NAD-MDH5	17.37988289	25.08467817	0.780394301
PEPCK4	13.26342553	21.12789375	0.747349812
NADP-MDH3	41.86358036	70.43570667	0.644783051
NAD-MDH6	109.0228806	181.0857437	0.622876821
NADP-ME10	0.230845944	6.508896668	0.619717123
NAD-MDH2	0.07722125	6.549410667	0.608917227
PEPC2	9.805194611	27.04810758	0.534580466
aCA10	0.269932472	12.08473358	0.46502406
NAD-MDH4	5.190244111	24.46470409	0.440747847
NAD-MDH11	6.638161972	31.17126192	0.404120768
NADP-ME5	5.380580806	29.51199592	0.389263575
NADP-ME1	16.6994858	92.32123007	0.260937889
PPCK2	4.990751139	49.11191025	0.253599504
NAD-MDH3	0.686906444	38.08443133	0.222252944
PPCK1	16.91778047	150.6700109	0.167534566
NADP-ME6	1.945218806	123.4454258	0.089513887
aCA5	17.46931944	418.9025437	0.064045597
aCA3	8.899020111	362.135913	0.050785263
NADP-ME3	5.625587305	386.3060163	0.039428085

Table S28. Expression of CAM gene families in *A. hybrid* at night and in the daytime.

Gene	Night	Day	（Night+10）/（Day+10)
PEPCK12	1058.05974	181.5451402	5.576021083
PEPCK3	865.6255477	157.4015252	5.23069038
PEPCK5	2178.960399	427.0549523	5.008432891
βCA5	160.8998498	92.40856938	1.668804192
aCA5	23.22474628	11.71389261	1.5301147

aCA9	10.58513394	4.224297222	1.447181089
βCA3	692.5632664	498.2697245	1.382264637
aCA3	11.77374483	6.024295389	1.358795773
NADP-ME3	7.443675444	3.807499167	1.263347927
NAD-MDH8	131.5542039	110.9424604	1.170426031
NAD-MDH10	179.3422071	153.1135415	1.16080005
βCA6	5.230755	3.801997278	1.103518186
NAD-MDH6	114.7810013	103.2647599	1.101675414
NAD-ME2	131.5313339	118.5263189	1.101185618
NADP-ME6	2.492497556	1.397940056	1.096031168
NAD-ME1	42.77162372	38.28485139	1.092922981
βCA7	10.64396294	9.159204777	1.077495814
NADP-MDH3	43.77934822	39.9478125	1.076710781
NADP-MDH1	62.04606272	57.48572933	1.067574781
βCA4	7.312599222	6.237047667	1.066240586
NAD-MDH11	7.146202222	6.130121722	1.062992736
NAD-MDH4	5.606884778	4.773603444	1.056403391
βCA1	1.525176945	0.990342389	1.048664049
NAD-ME3	197.1455672	187.5475403	1.048585909
γCA5	23.16460078	21.73931839	1.044905892
NAD-MDH3	0.859782167	0.514030722	1.032884766
aCA10	0.432169945	0.107695	1.032101774
γCA3	28.67271078	28.15933406	1.013453503
γCA4	11.80254517	11.5599325	1.011252942
γCA1	14.44609794	14.22121	1.009284753
NADP-ME10	0.259997722	0.201694167	1.005715086
aCA8	0.048837722	0.0038305	1.004498999
PPCK3	0.28482	0.274811667	1.000974065
aCA12	0	0	1
aCA2	0	0	1
aCA4	0	0	1
aCA6	5.872606111	5.875618722	0.999810237
NAD-MDH2	0.074825445	0.079617056	0.999524624
NADP-MDH4	340.3633032	341.0113135	0.998153877
PEPCK7	0.019376056	0.041080389	0.997838446
PEPCK2	0.070675833	0.118061833	0.995316692
NAD-MDH1	0.305193333	0.356015611	0.995092487
γCA2	42.61003028	42.94994667	0.993580421
NADP-MDH2	638.6019627	652.431956	0.979122394
NADP-ME5	5.160875611	5.600286	0.971833184
NAD-MDH12	24.32575589	25.33151295	0.971533711
γCA6	2.613530945	3.049686222	0.966577336
PEPCK6	0.113712833	0.490630278	0.964071039
PEPCK8	0.062734722	0.456281611	0.962362635
aCA1	0.728752667	1.233907333	0.95503304

aCA11	0.743016167	1.310026056	0.949866615
NADP-ME1	15.9609325	17.43803911	0.946165737
PEPC1	41.939745	44.91375717	0.945842129
γCA7	37.04377645	40.3048095	0.935174527
PEPC3	29.14852956	32.32502483	0.924949949
NAD-MDH9	22.48104978	25.15399333	0.923964725
aCA7	4.4698335	5.712093111	0.920936084
PEPC2	8.970243333	10.64014589	0.91909444
PPCK2	4.302619056	5.678883222	0.912221799
βCA2	7.610571056	9.423331555	0.90667098
NAD-MDH5	16.02510306	18.73466272	0.905704142
NAD-MDH7	28.81709545	33.39486961	0.894508862
PPDK2	647.6554718	734.2882351	0.883603208
NAD-MDH13	57.03221195	67.32435056	0.86689654
PPDK1	1635.932529	1910.392924	0.857081126
PPCK1	14.64291517	19.19264578	0.844148055
γCA8	13.2404505	18.07296834	0.82785868
PEPCK10	0.278267889	2.438965056	0.826296066
PEPC4	5326.986464	6679.403266	0.797826989
PEPCK1	0.745719667	3.946498223	0.770495898
PEPCK11	0.744537889	4.082482556	0.762971859
PEPCK9	0.484777056	3.879569056	0.75541085
NADP-ME4	22.07930678	33.2297755	0.742065079
NADP-ME2	122.046387	184.884967	0.677560661
PPDK-R1	5.180326112	12.8347835	0.664789579
PPDK-R2	7.248312222	17.84509789	0.619438017
PEPCK4	7.140484166	19.38636689	0.583280139

Table S29. Information on taxa included in this study.

Photosynthesis type	Species Name	Name in phylogenetic Tree	Soures
C3	*Amborella trichopoda* v1.0	AmTr	JGI
CAM	*Ananas comosus* v3	Aco	JGI
C3	*Arabidopsis thaliana* TAIR10	ATxG	JGI
C3	*Asparagus officinalis*	AsparagusV1	JGI
C3	*Brachypodium distachyon* v3.2	Bradi	JGI
CAM	*Kalanchoe fedtschenkoi* v1.1	Kaladp	JGI
CAM	*Kalanchoe laxiflora* v1.1	Kalax	JGI
C3	*Musa acuminata* v1	GSMUA	JGI
C3	*Oryza sativa* v7	LOC Os	JGI
CAM	*Portulaca amilis* v1.0	FUN	JGI
C4	*Setaria italica* v2.2	Seta	JGI
C4	*Sorghum bicolor* v3.1.1	Sobic	JGI

C3	*Vitis vinifera* v2.1	VIT	JGI
C4	*Zea mays* v4	Zm	JGI
CAM	*Hylocereus undatus*	HU	Chen et al., 2021
CAM	*Phalaenopsis aphrodite*	Paph	Chao et al., 2018
C3	*Apostasia shenzhenica*	Ashen	Chao et al., 2018
C3/CAM	*Dendrobium catenatum*	Dcat	Chao et al., 2018
CAM	*Phalaenopsis equestris*	Pequ	Cai et al., 2015
C3	*Asparagus setaceus*	OF	Li et al., 2020
CAM	*Agave hybrid*	EVM	This study
CAM	*Agave americana* L.	Aamer	This study
CAM	*Agave fourcroydes* Lem.	Afou	This study
CAM	*Agave neglecta*	Anegl	This study
CAM	*Agave desmetiana* hort. ex Baker (1877)	Adesm	This study
CAM	*Agave potatorum* Zucc.	Apota	This study
CAM	*Agave attenuata* Salm-Dyck	Aatte	This study
CAM	*Agave amanuensis*	Aaman	This study
CAM	*Agave cantala* Roxb.	Acant	This study
CAM	*A.hybrid* 'nanya NO.1'	RemaNO1	This study
CAM	*A.hybrid* 'yuexi No.114'	Yx114	This study
CAM	*A.hybrid* 76416	Gx76416	This study
CAM	*A.hybrid* H1002	H1002	This study
CAM	*A.hybrid* S0908	S0908	This study
CAM	*A.hybrid* D06-556	D06-556	This study
CAM	*Agave angustifolia* Haw	Aangu	This study
CAM	*Agave deserti*	Adeser	Gross. et al., 2013
CAM	*Agave tequilana*	Atequ	Gross. et al., 2013
CAM	*Agave sisalana*	Asisa	Sarwar. et al., 2019

Supplementary Figures

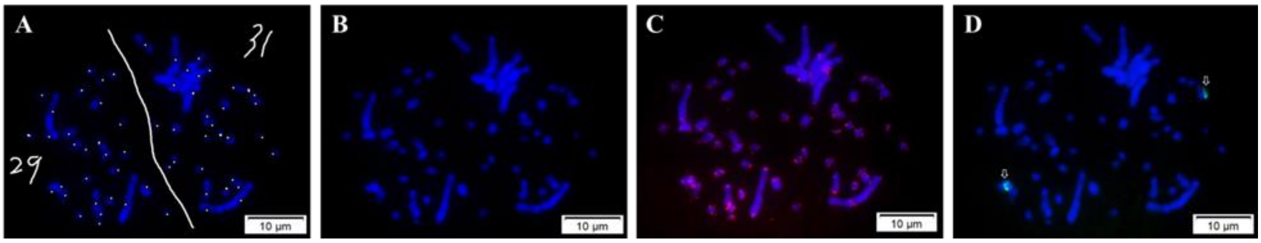

Figure S1. Cytological analysis of *A. hybrid* chromosomes. **(A)** Chromosome numbers of *A. hybrid*. **(B)** DAPI staining of *A. hybrid* chromosome. **(C)** Fluorescence in situ hybridization (FISH) assay using telomere repeat sequence as probes. **(D)** FISH assay using 5S rDNA as probes.

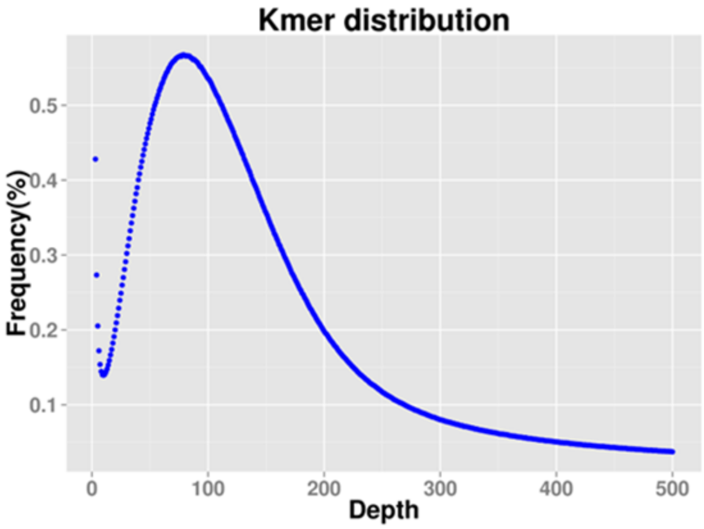

Figure S2. Distribution of 21-k-mers from the Illumina data

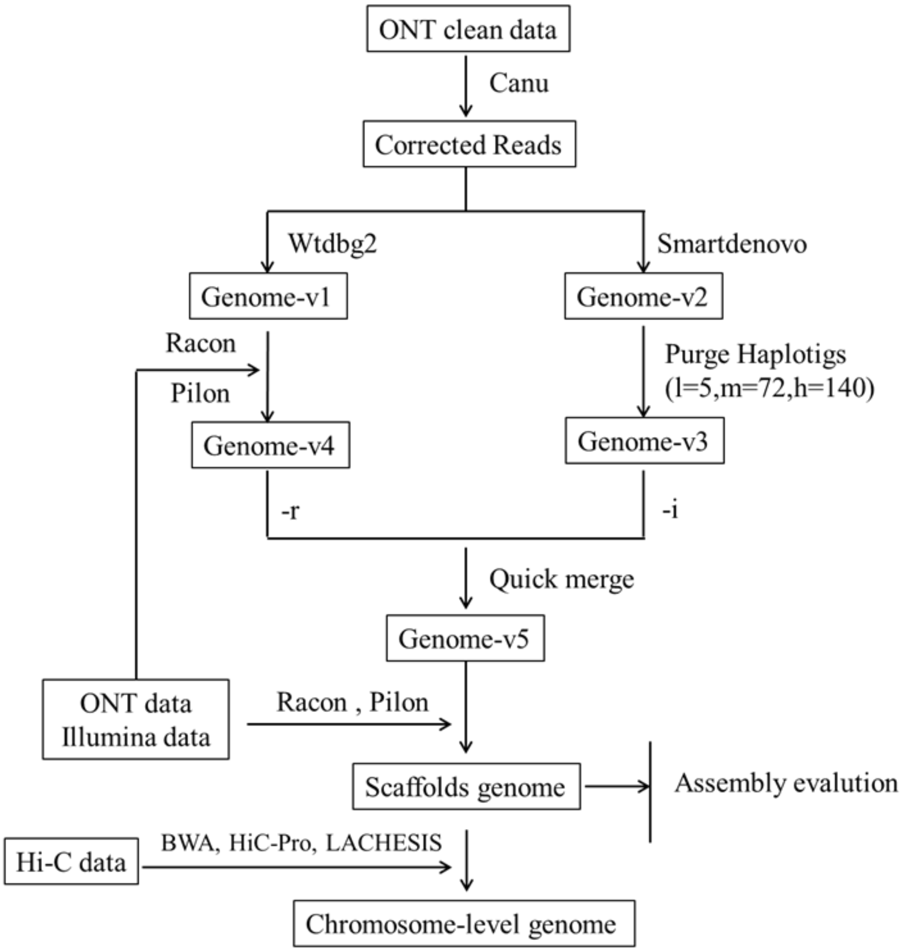

Figure S3. An overview of genome assembly workflow

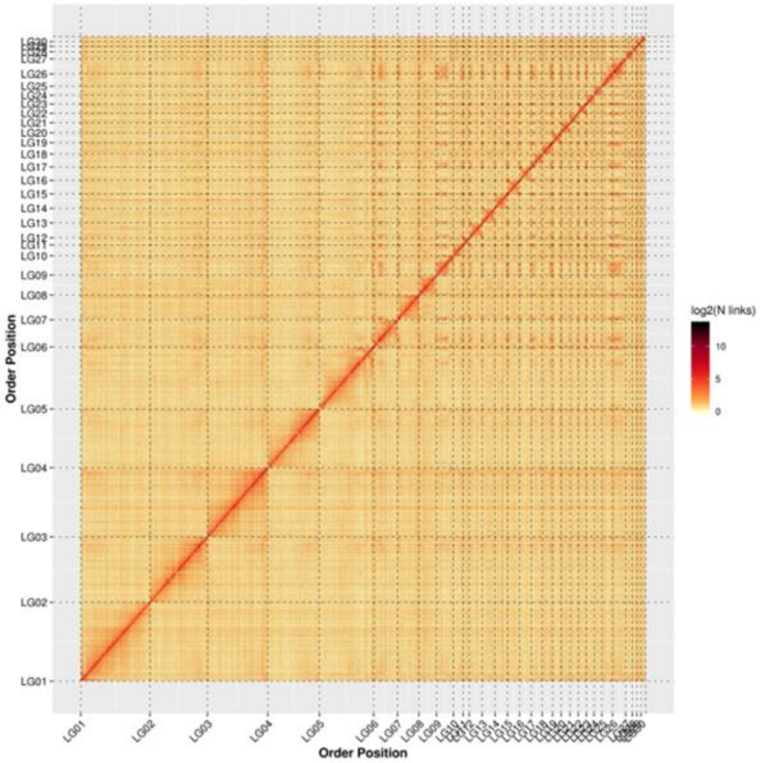

Figure S4. The genome-wide Hi-C heatmap of *A. hybrid.* LG1-30 are the abbreviations for Lachesis Group1-30, representing the 30 pseudochromosomes.

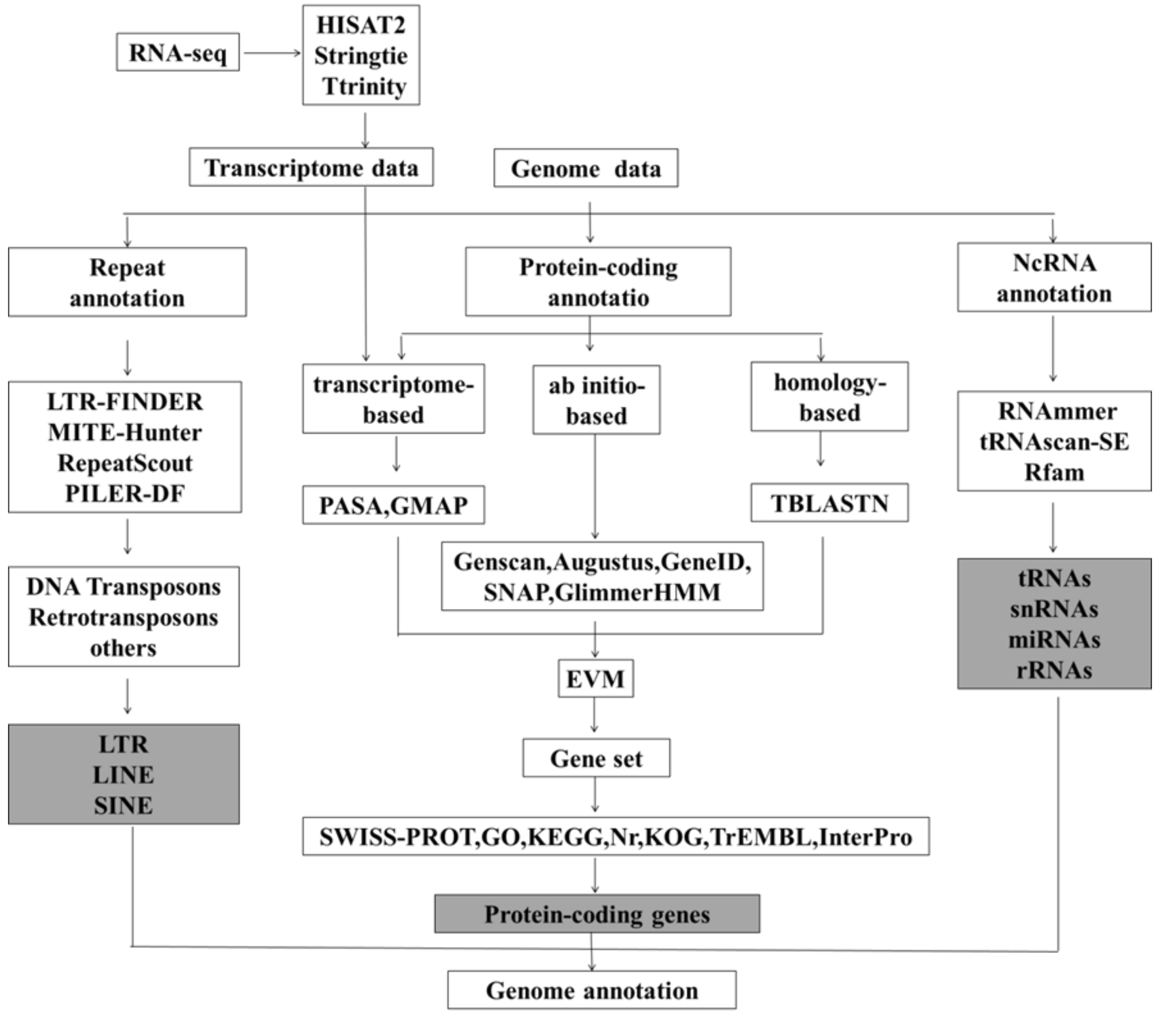

Figure S5. An overview of genome annotation workflow

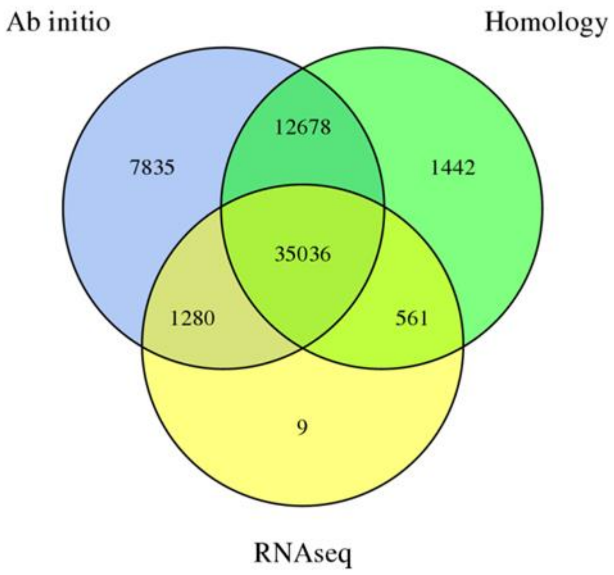

Figure S6. Distribution map of integrated gene numbers derived from three prediction methods.

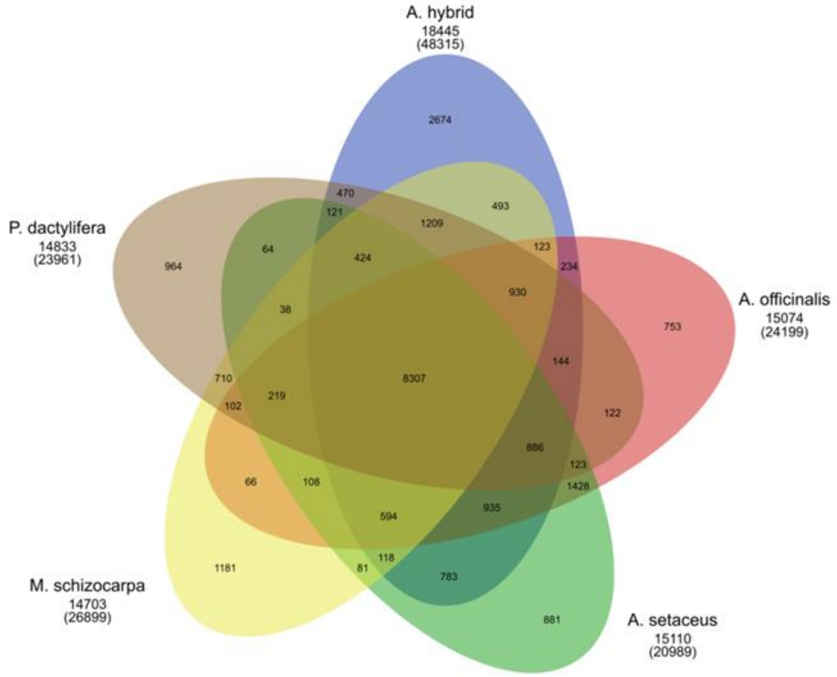

Figure S7. Venn diagram of orthologous gene families among *A. hybrid*, *A. officinalis*, *A. setaceus*, *P. dactylifera*, and *M. schizocarpa*.

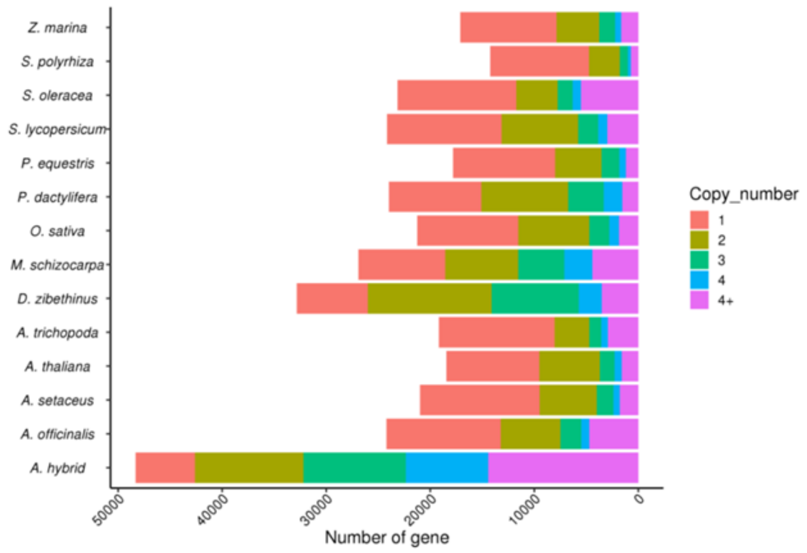

Figure S8. Distribution of genes and gene families of 14 plant species.

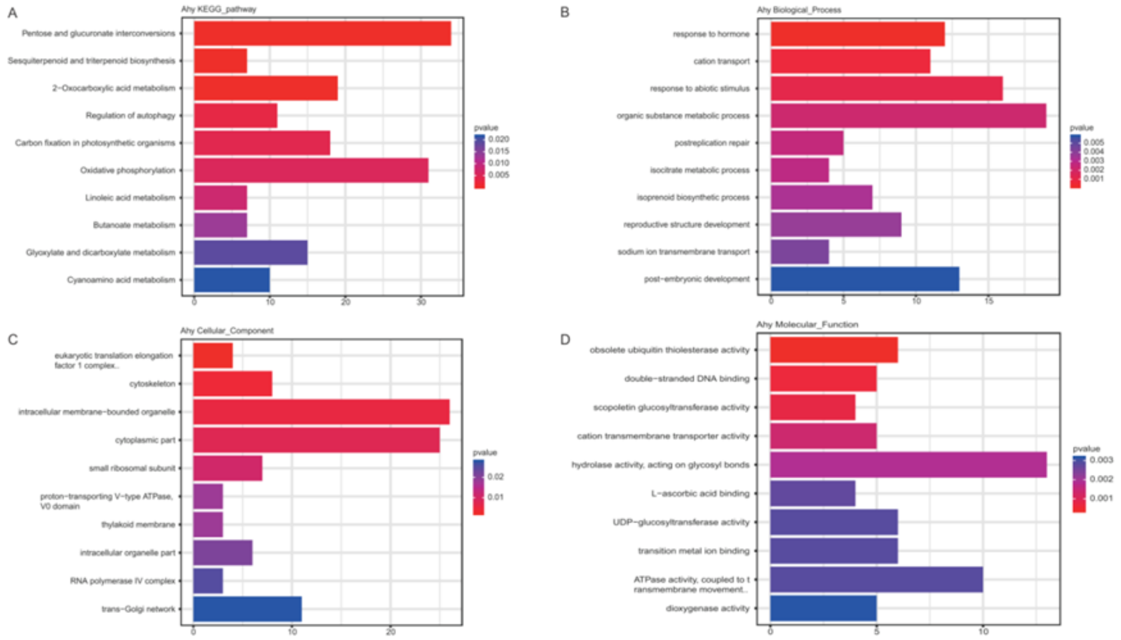

Figure S9. Enrichment analysis of unique gene families from *A. hybrid* genome. (A-D) KEGG pathway, GO biological process, GO cellular component, and GO molecular function enrichment of unique gene families from *A. hybrid* genome.

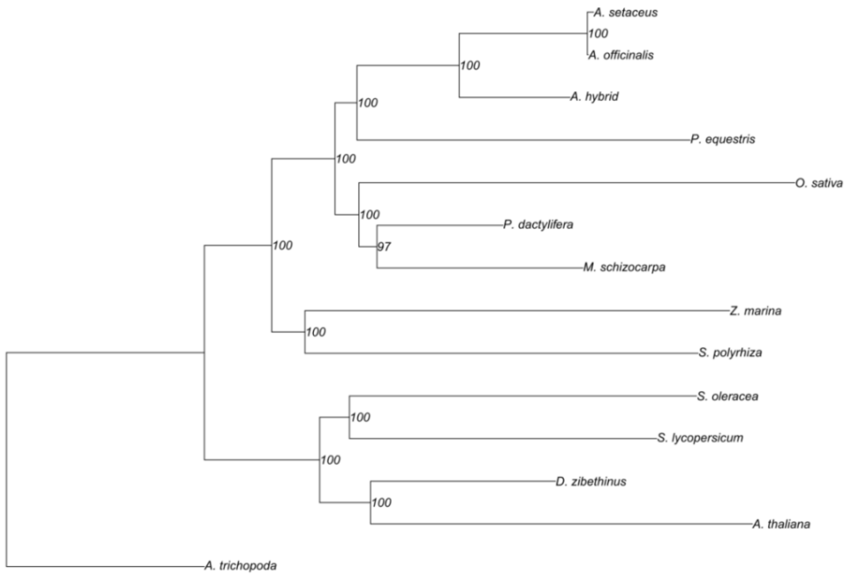

Figure S10. Phylogenetic tree constructed with orthologs. The taxon names in the phylogenetic tree are listed in Supplementary Table 26.

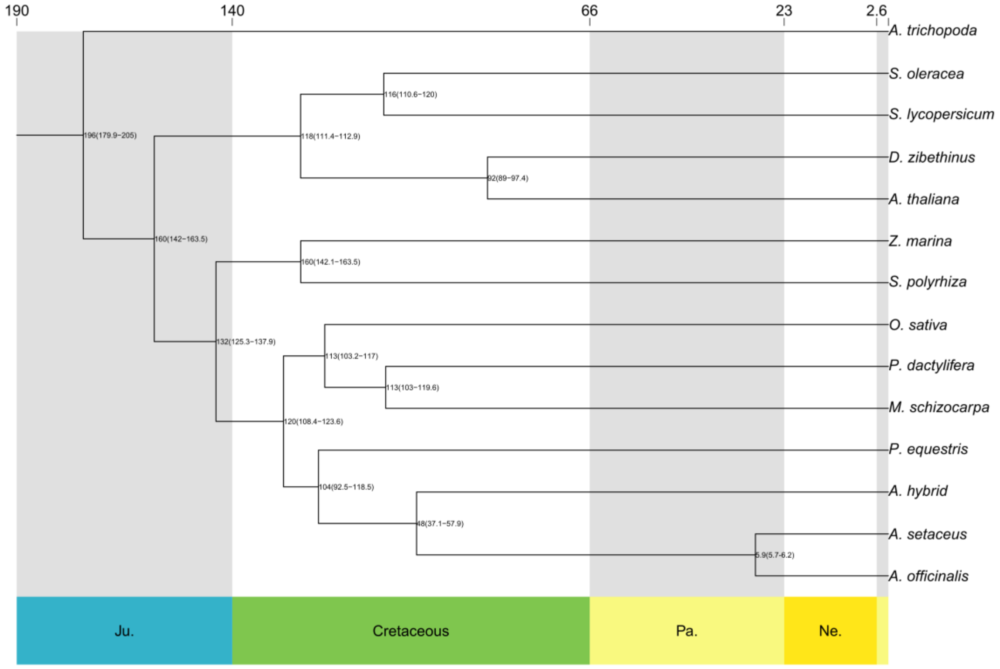

Figure S11. Divergence time estimated for *A. hybrid*. The taxon names in the phylogenetic tree are listed in Supplementary Table 26. Ju, Pa, and Ne are abbreviations for Jurassic, Paleogene, and Neogene respectively.

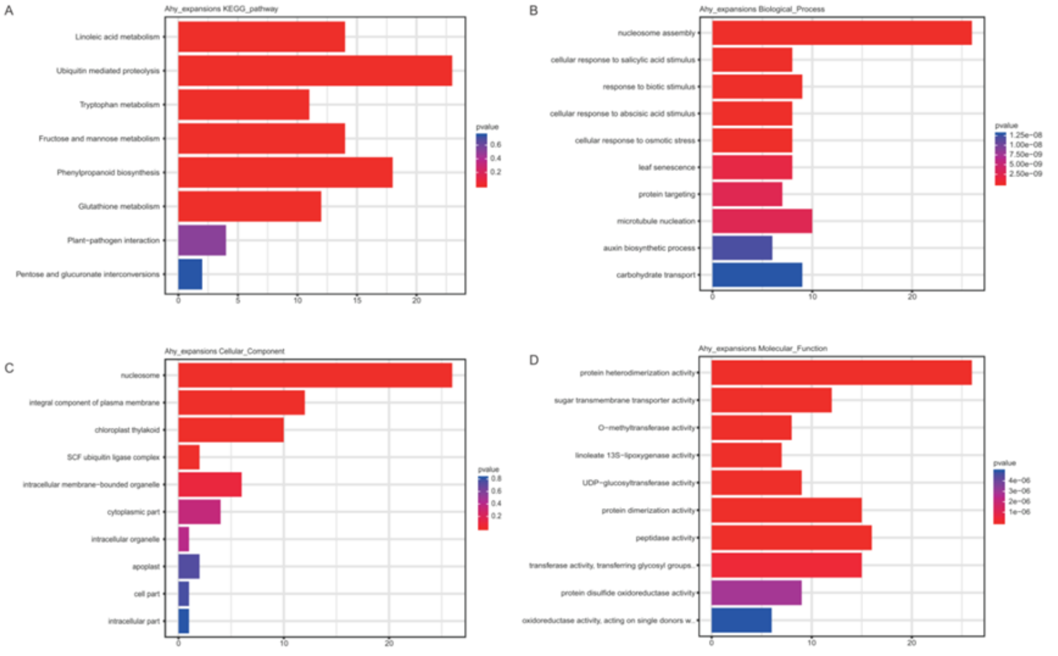

Figure S12. Enrichment analysis of significantly expanded gene families in *A. hybrid* genome. (A-D) KEGG pathway, GO biological process, GO cellular component, and GO molecular function enrichment of significantly expanded gene families in *A. hybrid* genome.

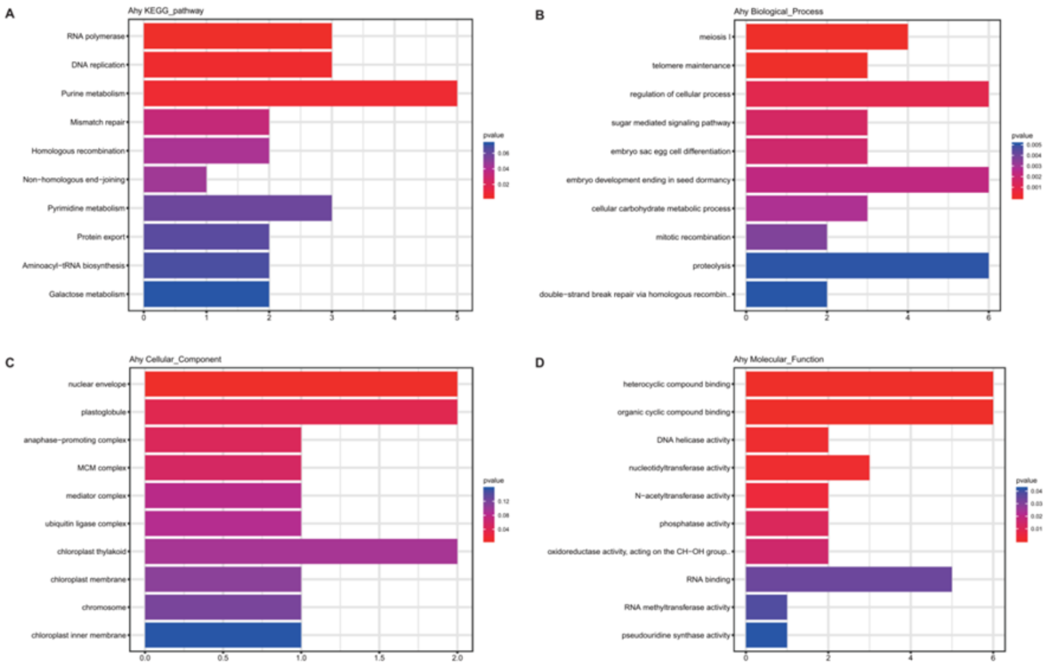

Figure S13. Enrichment analysis of positive selection gene families from *A. hybrid* genome. (A-D) KEGG pathway, GO biological process, GO cellular component, and GO molecular function enrichment of positive selection gene families from *A. hybrid* genome.

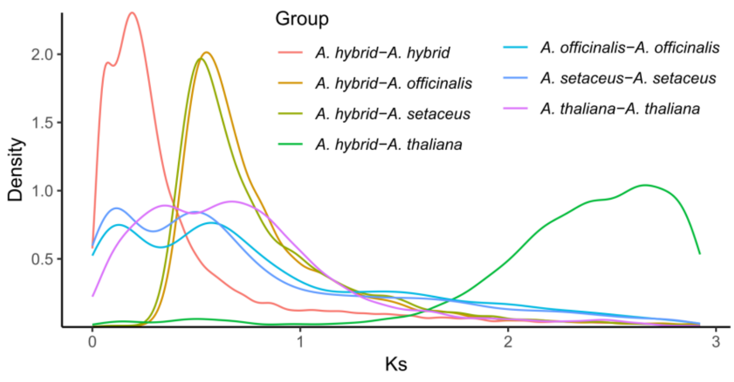

Figure S14. Ka/Ks distribution in *A. hybrid* and other representative plant species.

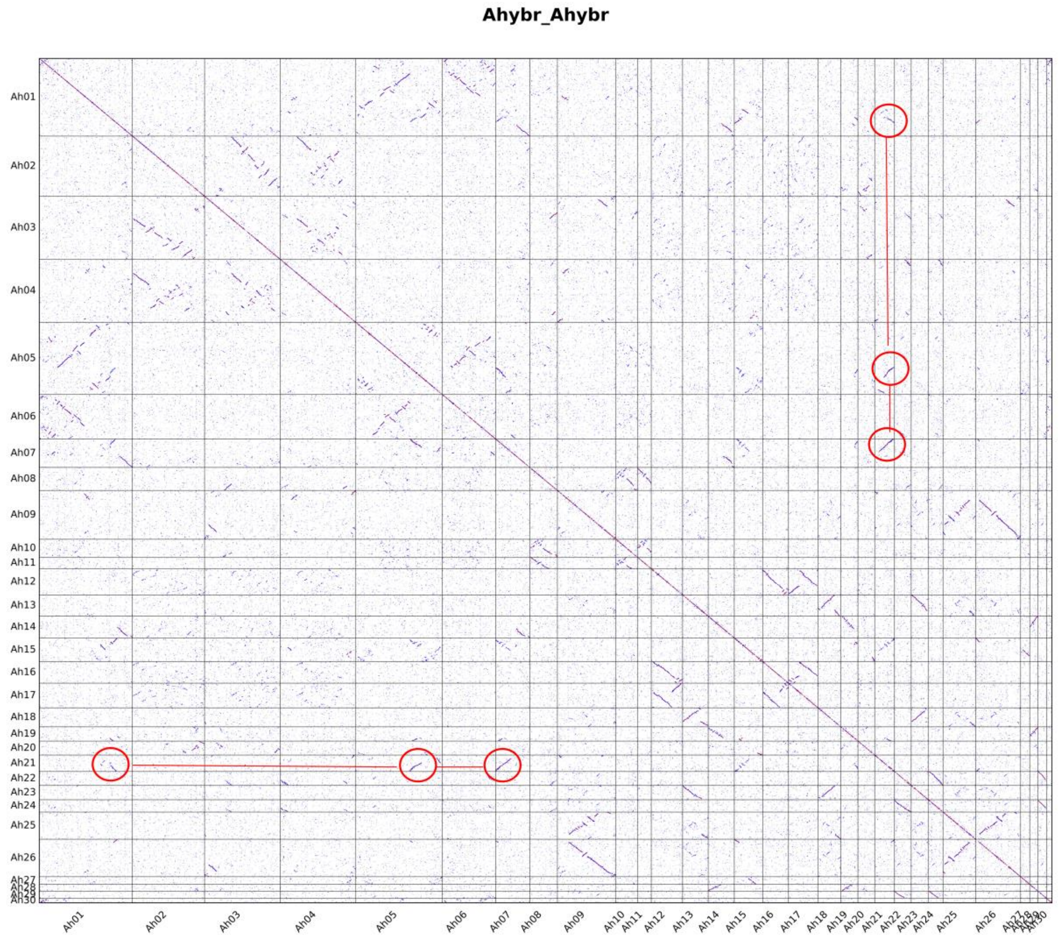

Figure S15 Synteny analysis within *A. hybrid* genomes.

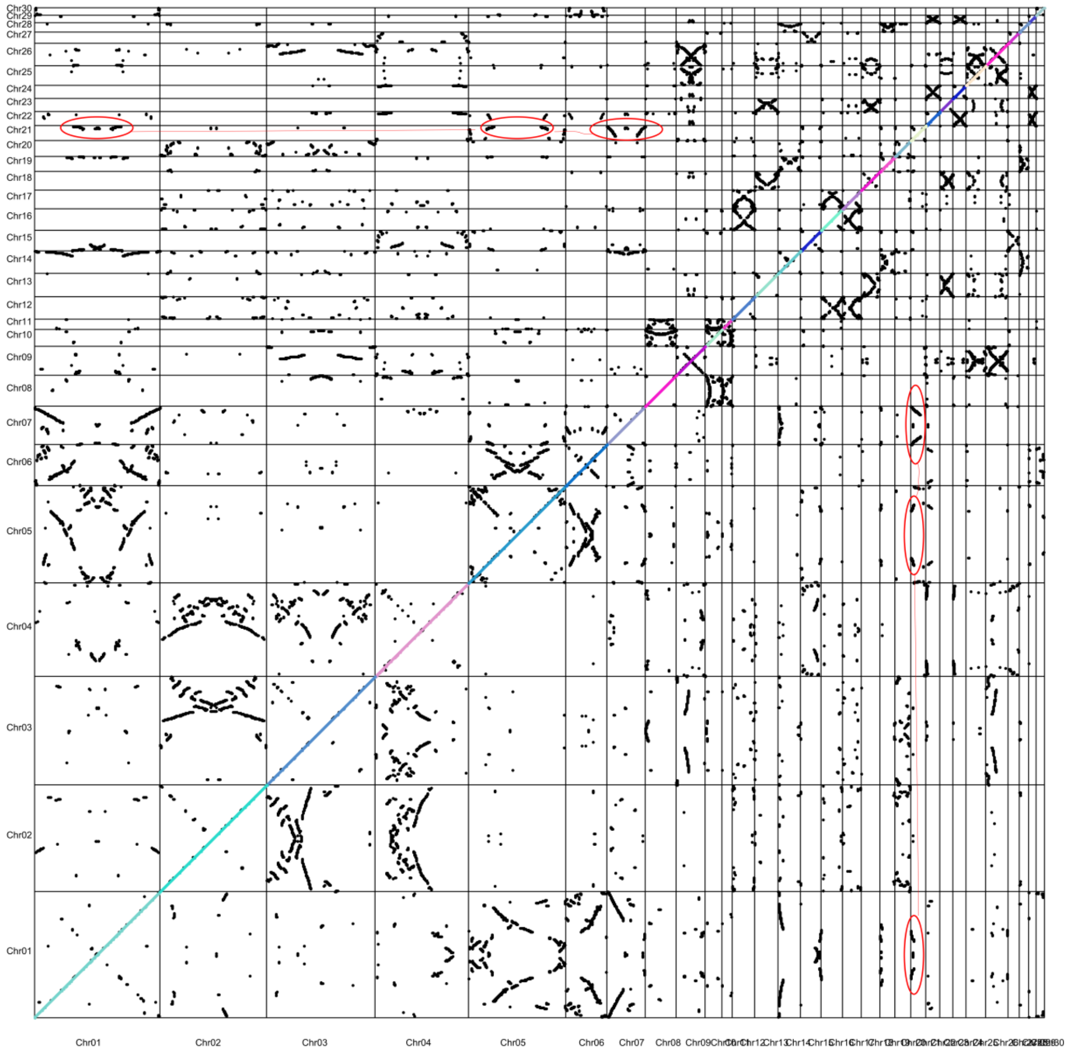

Figure S16 Synteny analysis within *A. hybrid* genomes.

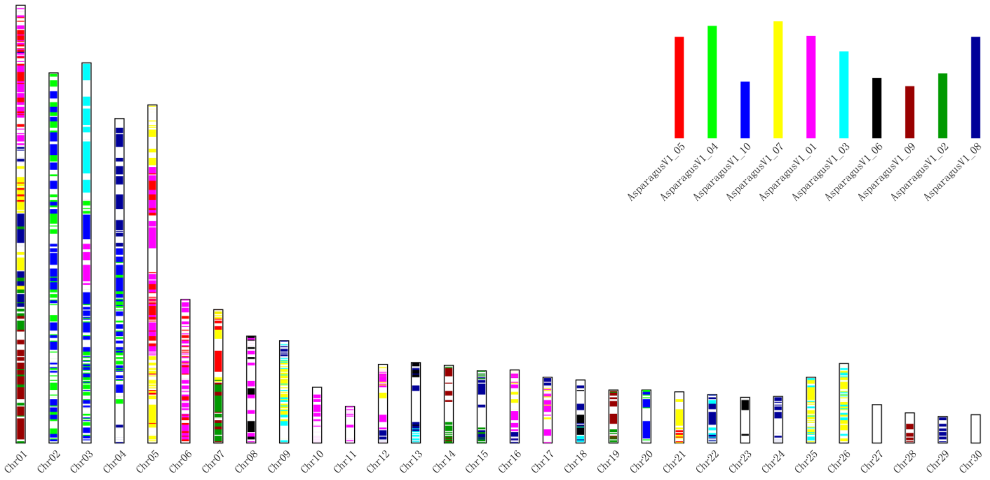

Figure S17. Comparison of *A. hybrid* genome with *A. setaceus* genome.

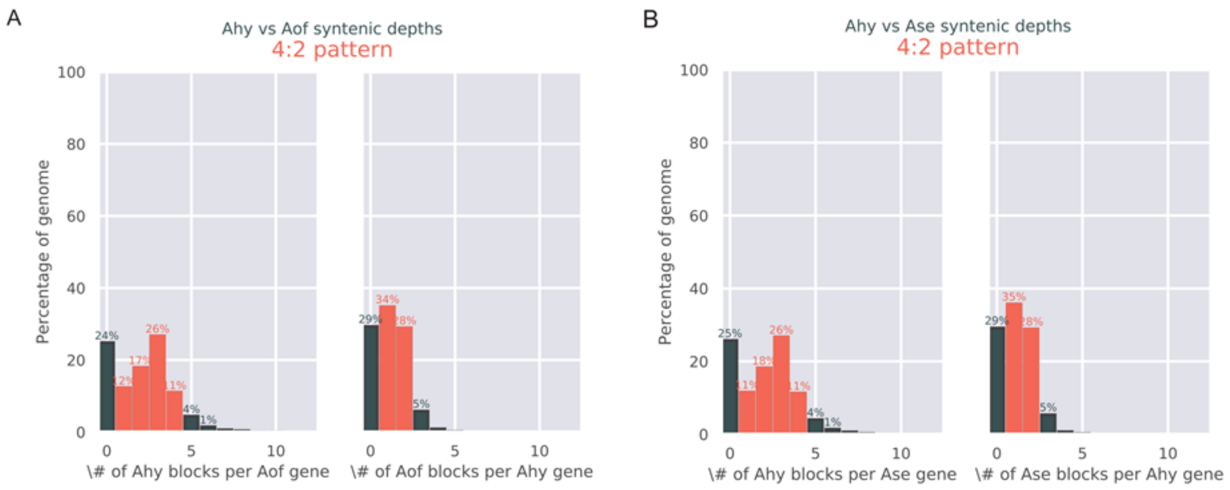

Figure S18. The synteny relationship between *A. hybrid* and *A. officinalis* (A) and between *A. hybrid* and *A. setaceus* (B).

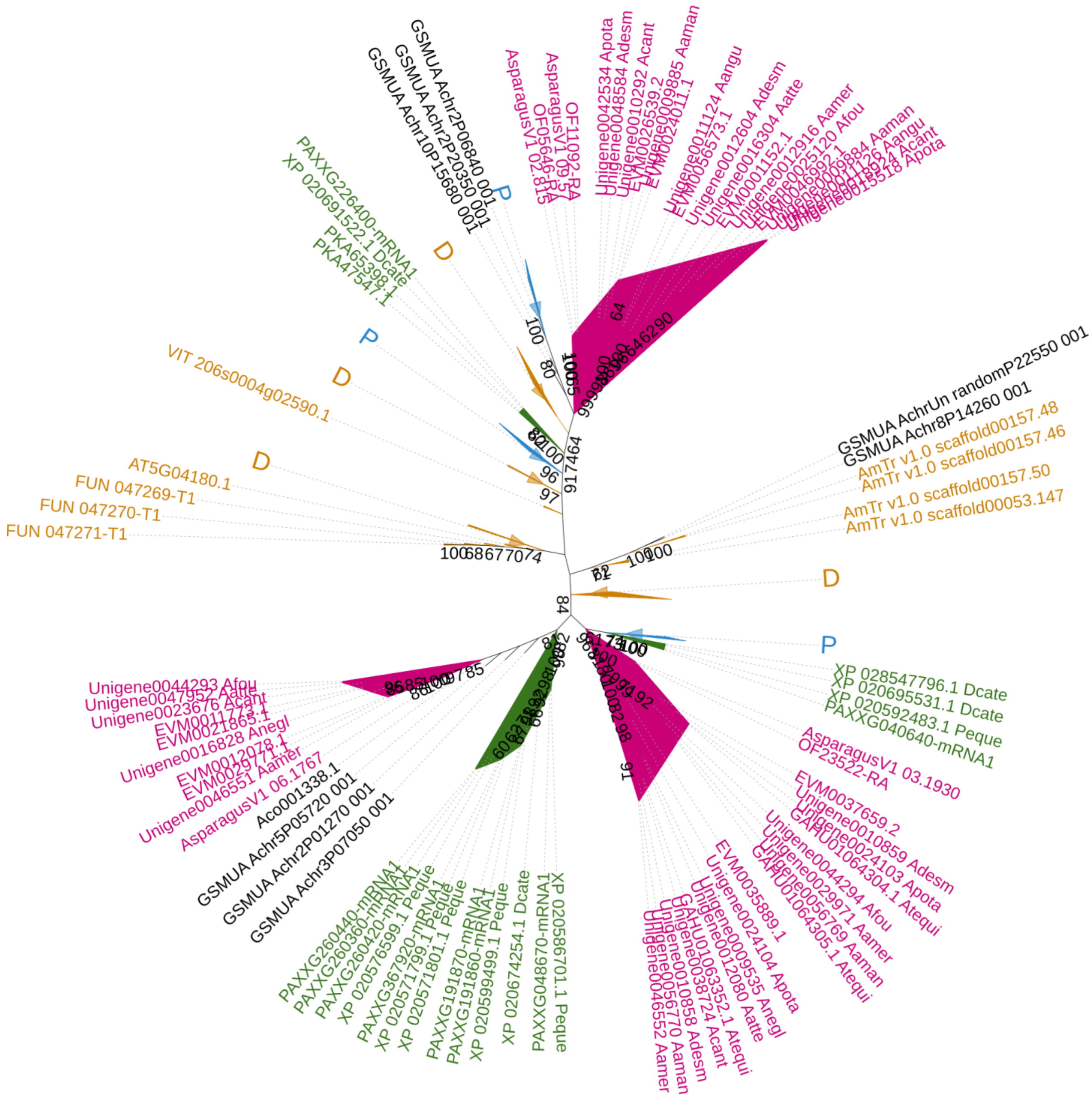

Figure S19. Phylogenetic tree of ***α CA*** **gene family.** The abbreviated gene names are listed in column of information on taxa included in this study in Table S29. Asparagaceae, Orchidaceae, dicotyledons, and Poales (monocotyledons) are respectively marked in red, green, brown, and blue. Among these, Asparagaceae and Orchidaceae belong to Asparagales. D and P are the initial letters of Dicotyledons and Poales.

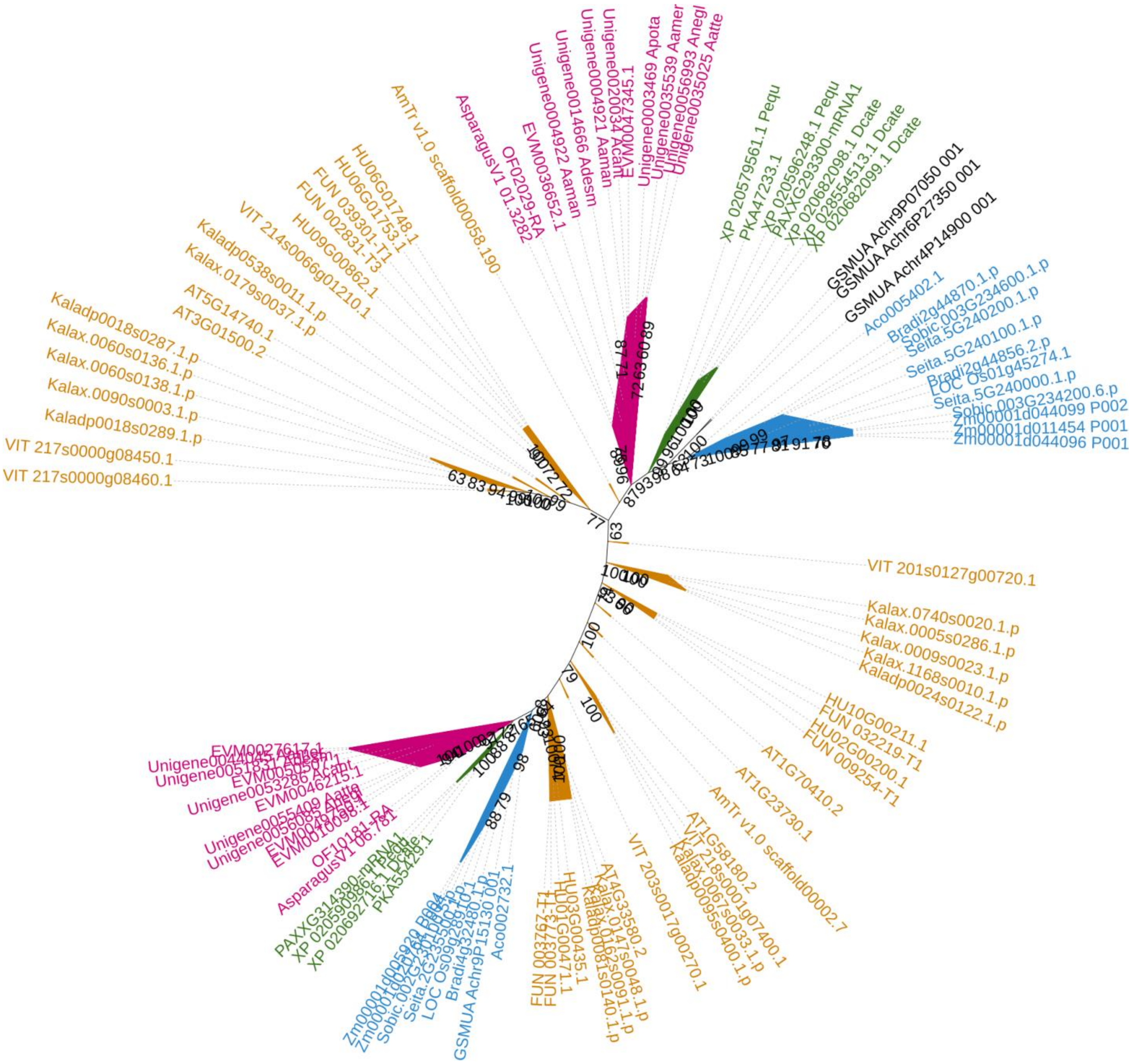

Figure S20. Phylogenetic tree of β***CA* gene family.** The abbreviated gene names are listed in column of information on taxa included in this study in Table S29. Asparagaceae, Orchidaceae, dicotyledons, and Poales (monocotyledons) are respectively marked in red, green, brown, and blue. Among these, Asparagaceae and Orchidaceae belong to Asparagales.

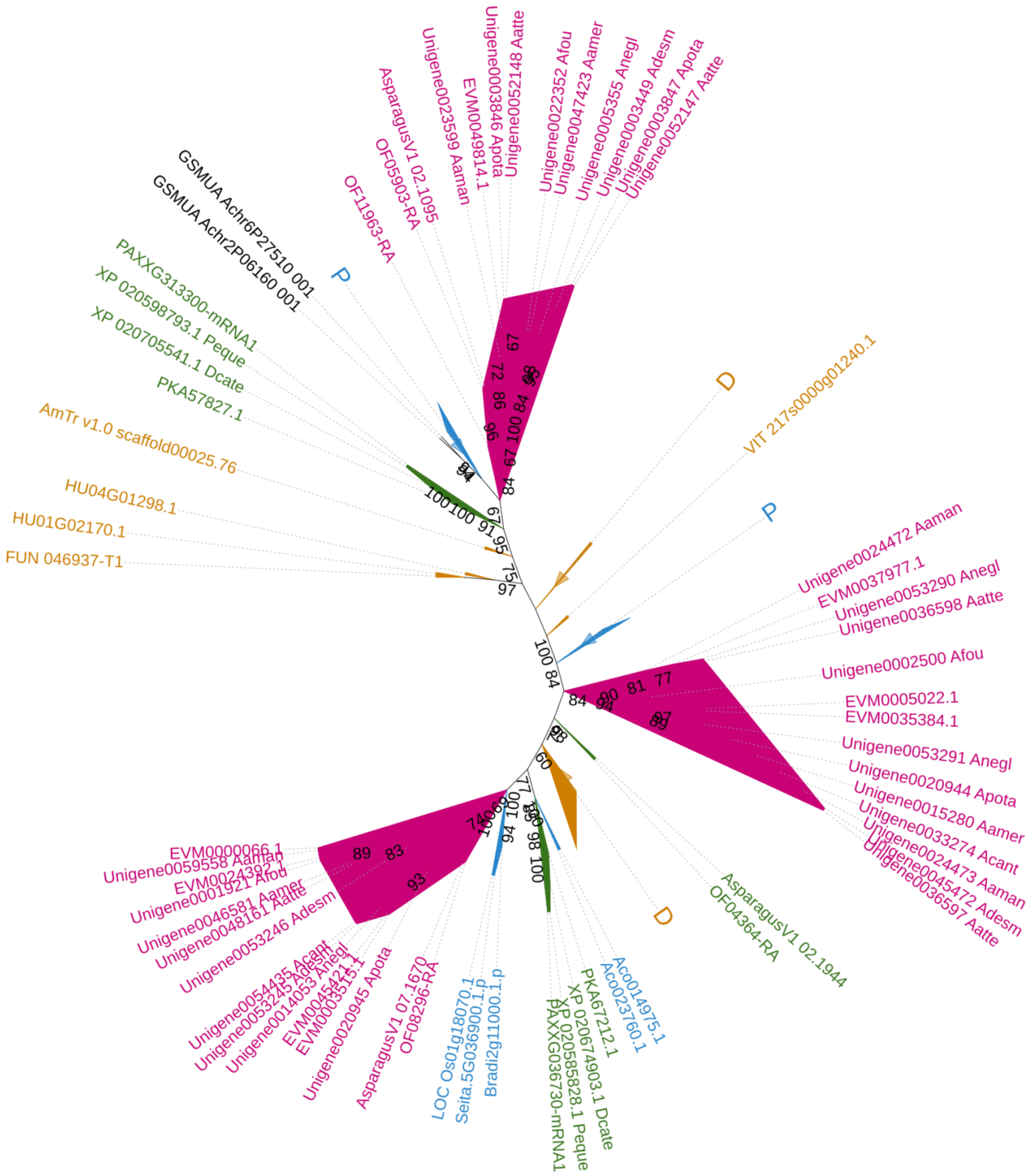

Figure S21. Phylogenetic tree of *γCA* gene family. The abbreviated gene names are listed in column of information on taxa included in this study in Table S29. Asparagaceae, Orchidaceae, dicotyledons, and Poales (monocotyledons) are respectively marked in red, green, brown, and blue. Among these, Asparagaceae and Orchidaceae belong to Asparagales. D and P are the initial letters of Dicotyledons and Poales.

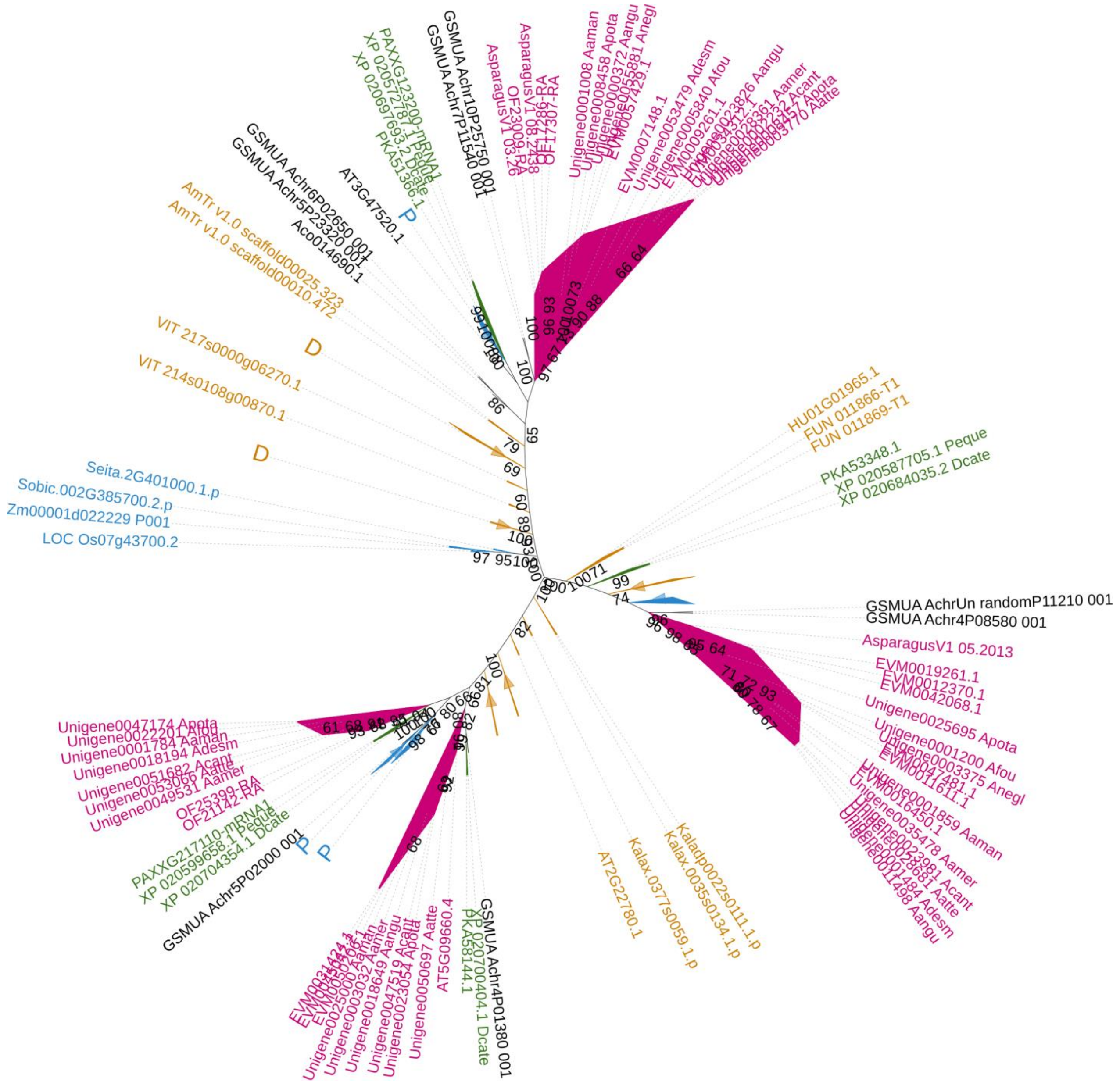

Figure S22. Phylogenetic tree of *NAD-MDH* gene family. The abbreviated gene names are listed in column of information on taxa included in this study in Table S29. Asparagaceae, Orchidaceae, dicotyledons, and Poales (monocotyledons) are respectively marked in red, green, brown, and blue. Among these, Asparagaceae and Orchidaceae belong to Asparagales. D and P are the initial letters of Dicotyledons and Poales.

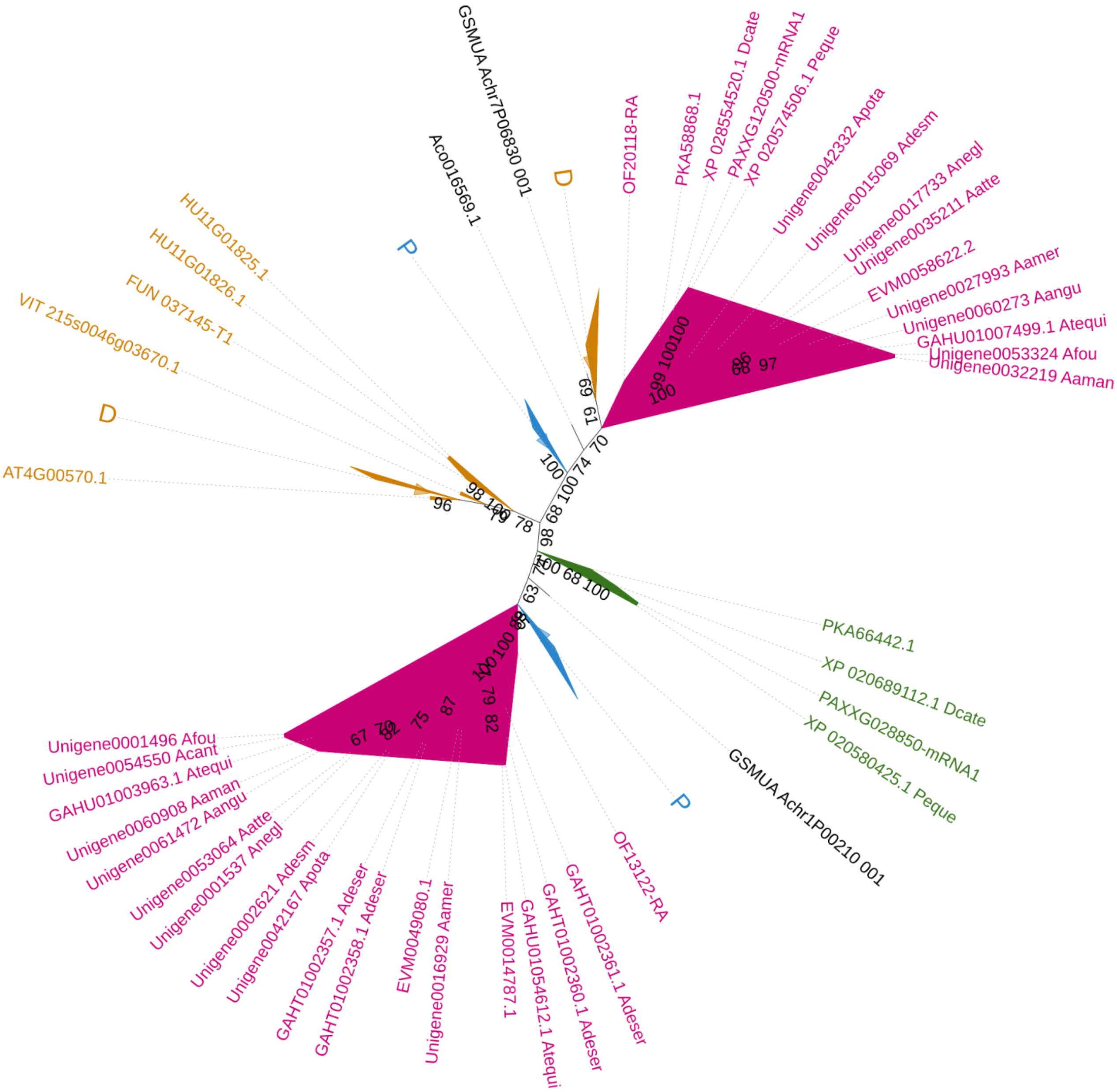

Figure S23. Phylogenetic tree of *NAD-ME* gene family. The abbreviated gene names are listed in column of information on taxa included in this study in Table S29. Asparagaceae, Orchidaceae, dicotyledons, and Poales (monocotyledons) are respectively marked in red, green, brown, and blue. Among these, Asparagaceae and Orchidaceae belong to Asparagales. D and P are the initial letters of Dicotyledons and Poales.

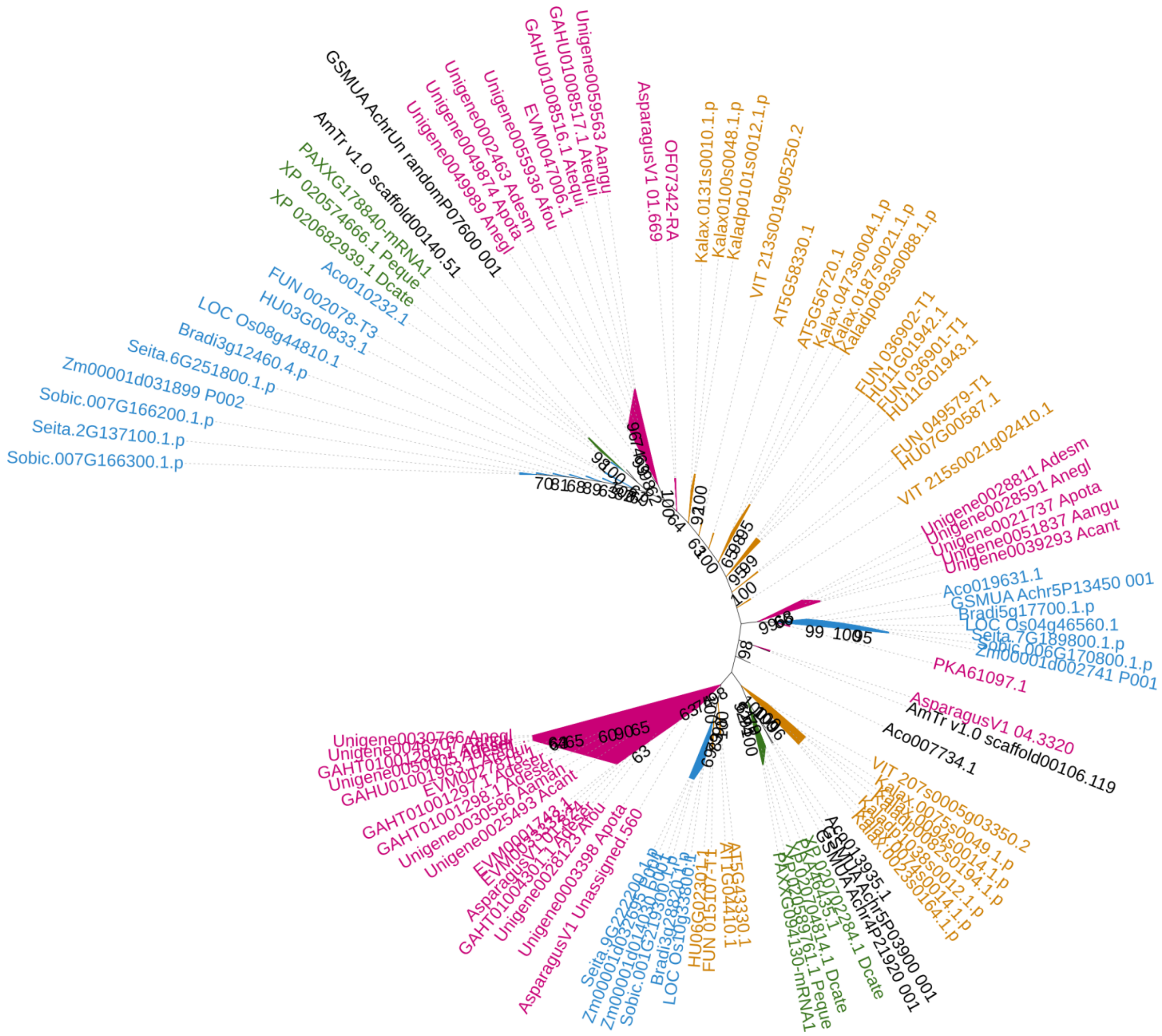

Figure S24. Phylogenetic tree of *NADP-MDH* gene family. The abbreviated gene names are listed in column of information on taxa included in this study in Table S29. Asparagaceae, Orchidaceae, dicotyledons, and Poales (monocotyledons) are respectively marked in red, green, brown, and blue. Among these, Asparagaceae and Orchidaceae belong to Asparagales.

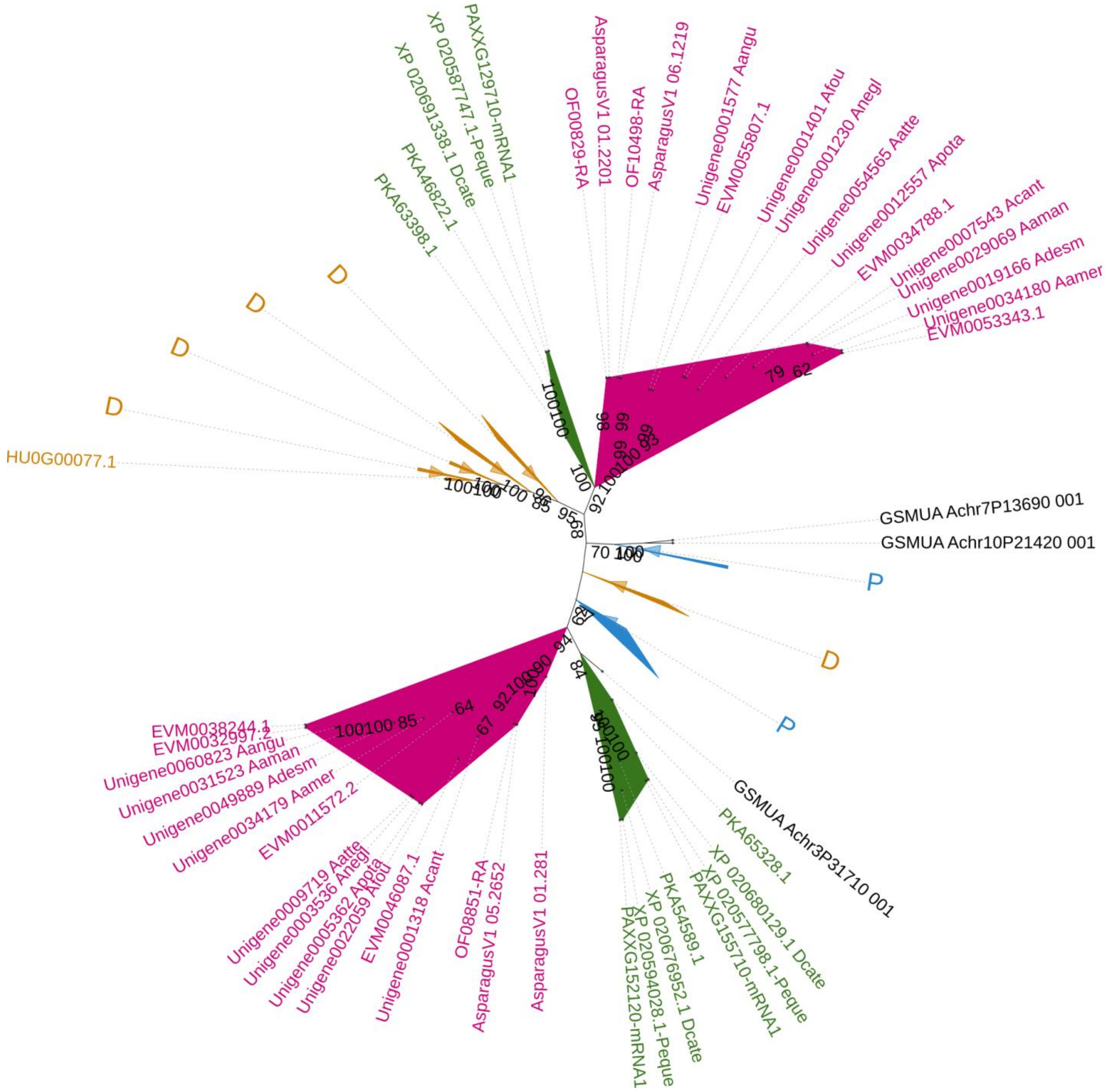

Figure S25. Phylogenetic tree of *NADP-ME* gene family. The abbreviated gene names are listed in column of information on taxa included in this study in Table S29. Asparagaceae, Orchidaceae, dicotyledons, and Poales (monocotyledons) are respectively marked in red, green, brown, and blue. Among these, Asparagaceae and Orchidaceae belong to Asparagales. D and P are the initial letters of Dicotyledons and Poales.

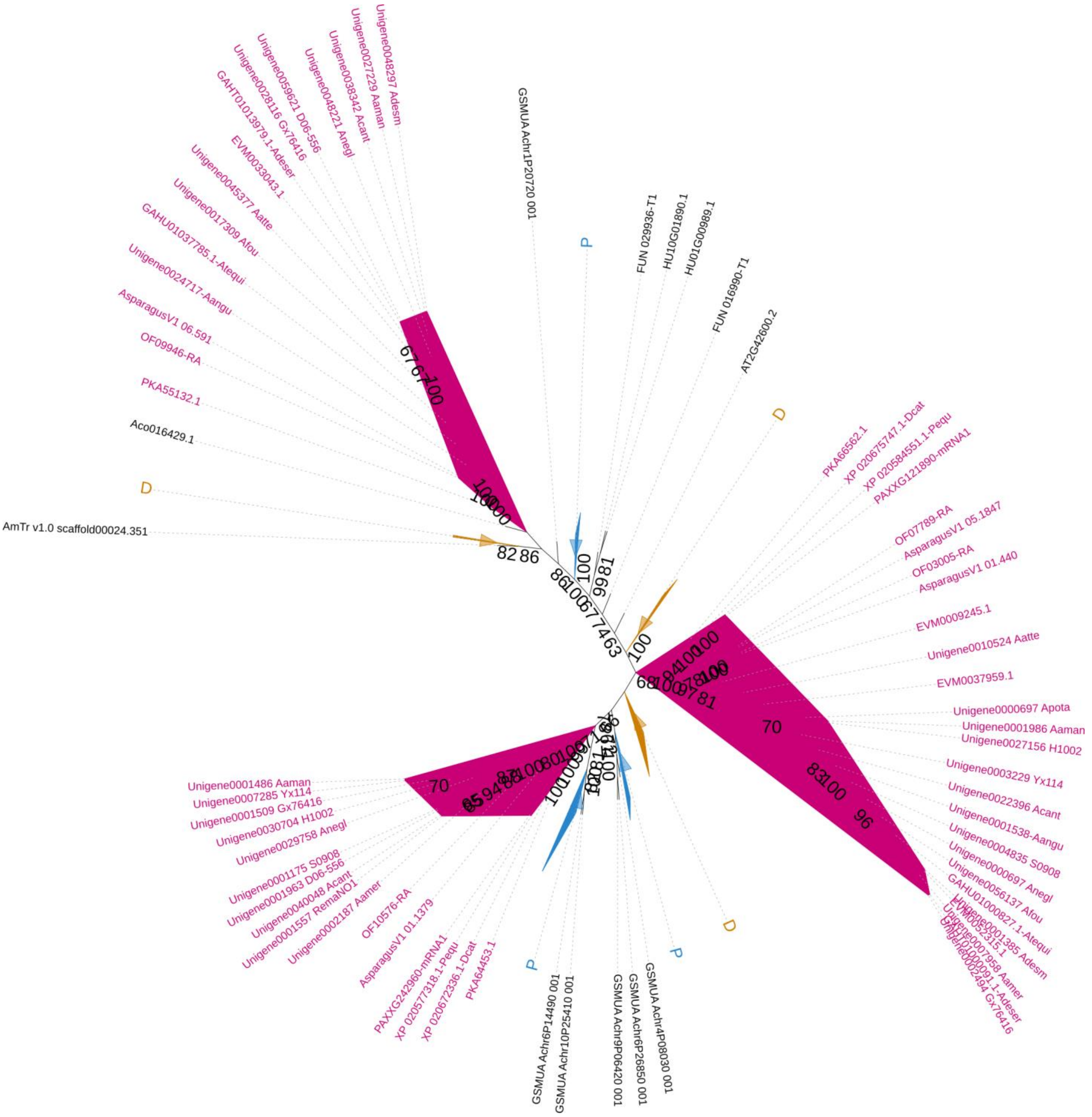

Figure S26. Phylogenetic tree of *PEPC* gene family. The abbreviated gene names are listed in column of information on taxa included in this study in Table S29. Asparagaceae, Orchidaceae, dicotyledons, and Poales (monocotyledons) are respectively marked in red, green, brown, and blue. Among these, Asparagaceae and Orchidaceae belong to Asparagales. D and P are the initial letters of Dicotyledons and Poales.

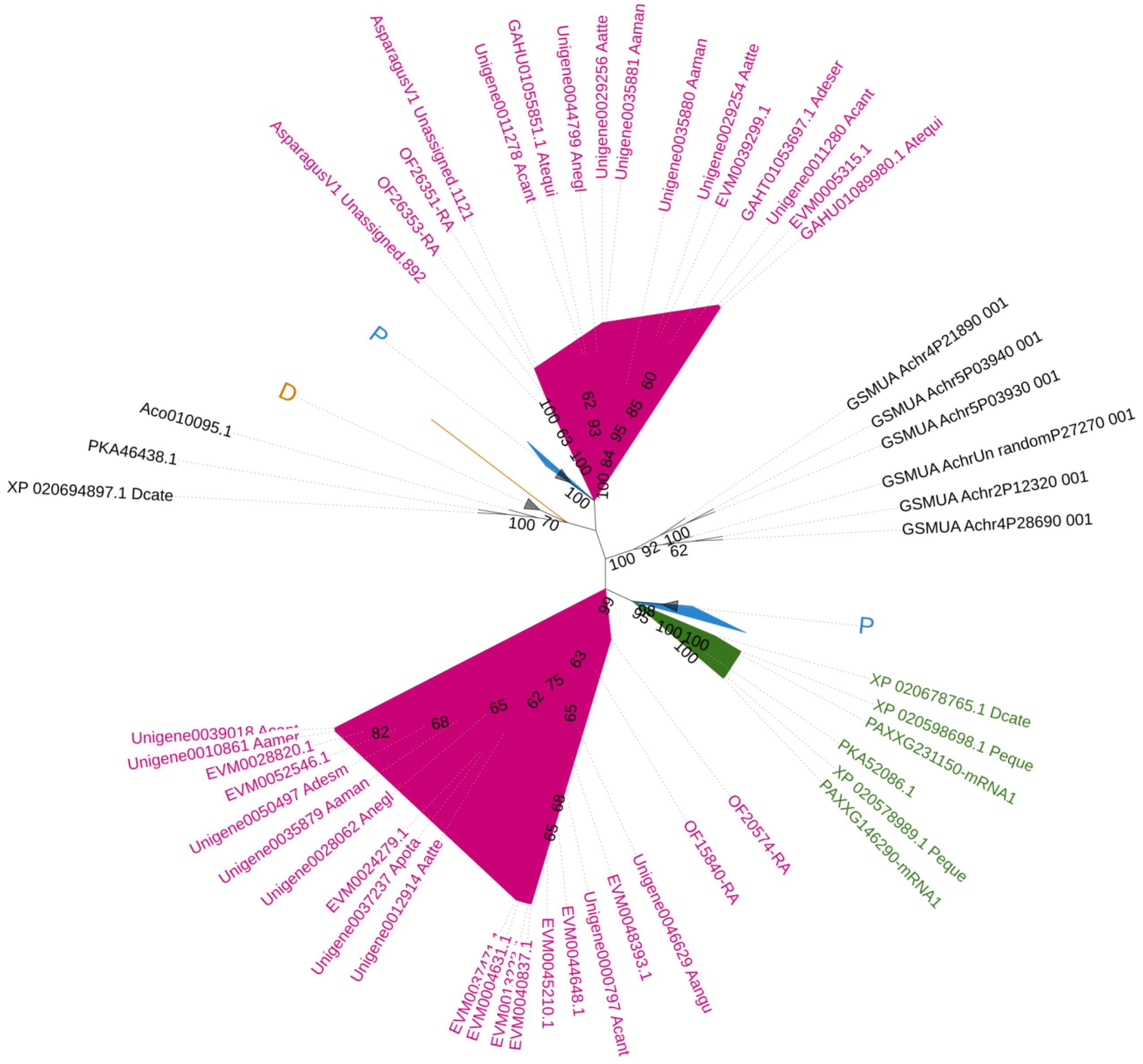

Figure S27. Phylogenetic tree of *PEPCK* gene family. The abbreviated gene names are listed in column of information on taxa included in this study in Table S29. Asparagaceae, Orchidaceae, dicotyledons, and Poales (monocotyledons) are respectively marked in red, green, brown, and blue. Among these, Asparagaceae and Orchidaceae belong to Asparagales. D and P are the initial letters of Dicotyledons and Poales.

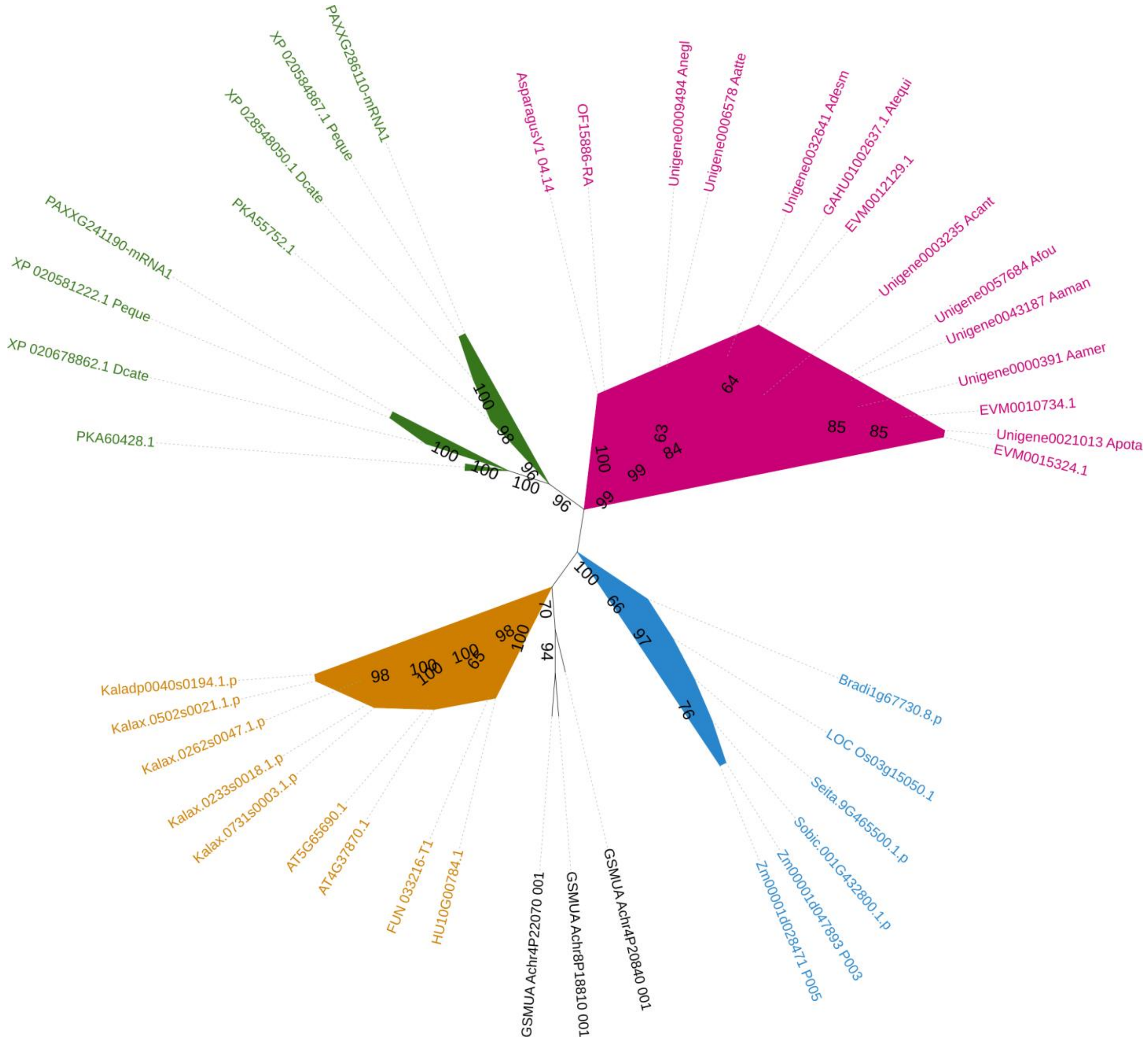

Figure S28. Phylogenetic tree of *PPCK* gene family. The abbreviated gene names are listed in column of information on taxa included in this study in Table S29. Asparagaceae, Orchidaceae, dicotyledons, and Poales (monocotyledons) are respectively marked in red, green, brown, and blue. Among these, Asparagaceae and Orchidaceae belong to Asparagales.

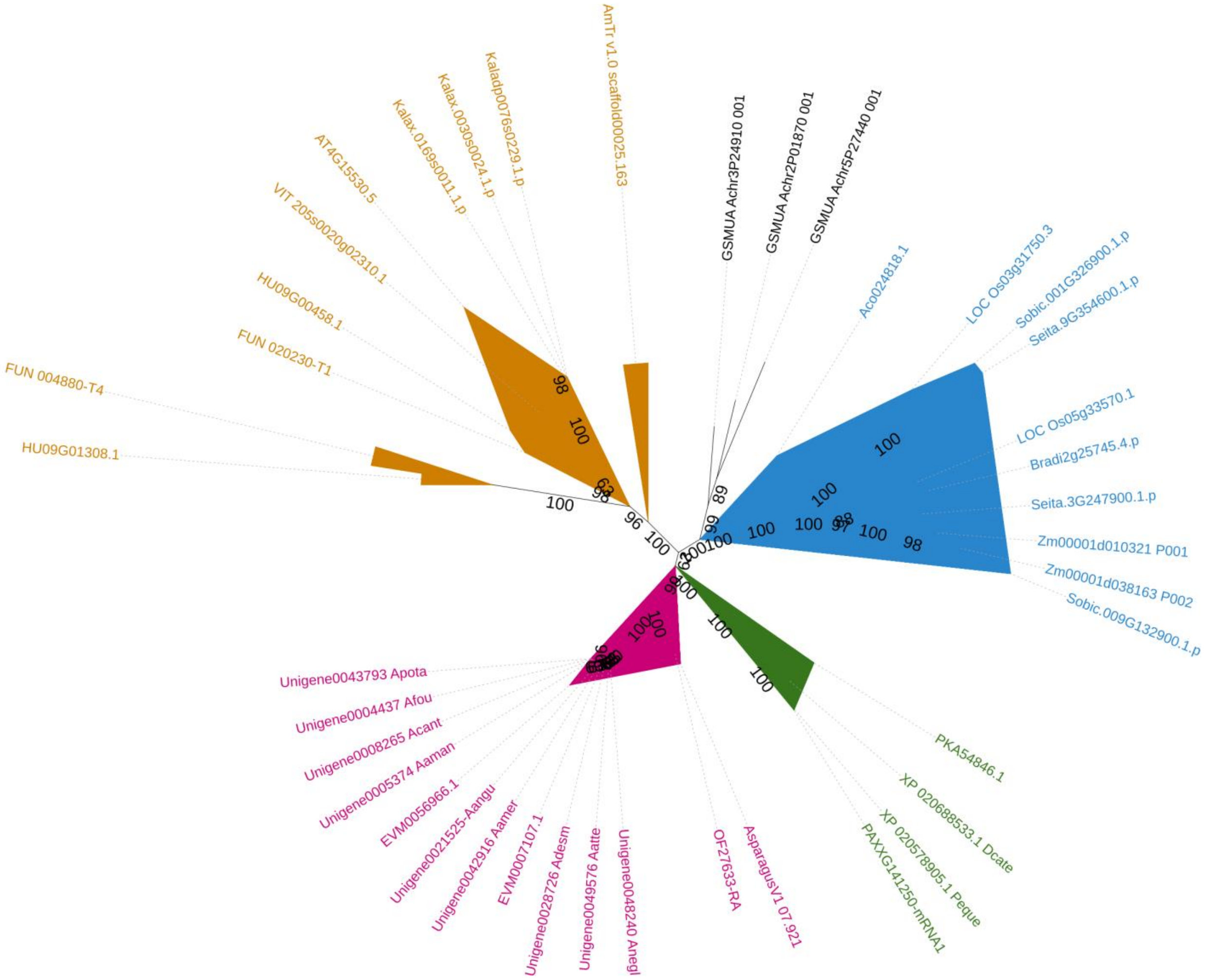

Figure S29. Phylogenetic tree of *PPDK* gene family. The abbreviated gene names are listed in column of information on taxa included in this study in Table S29. Asparagaceae, Orchidaceae, dicotyledons, and Poales (monocotyledons) are respectively marked in red, green, brown, and blue. Among these, Asparagaceae and Orchidaceae belong to Asparagales.

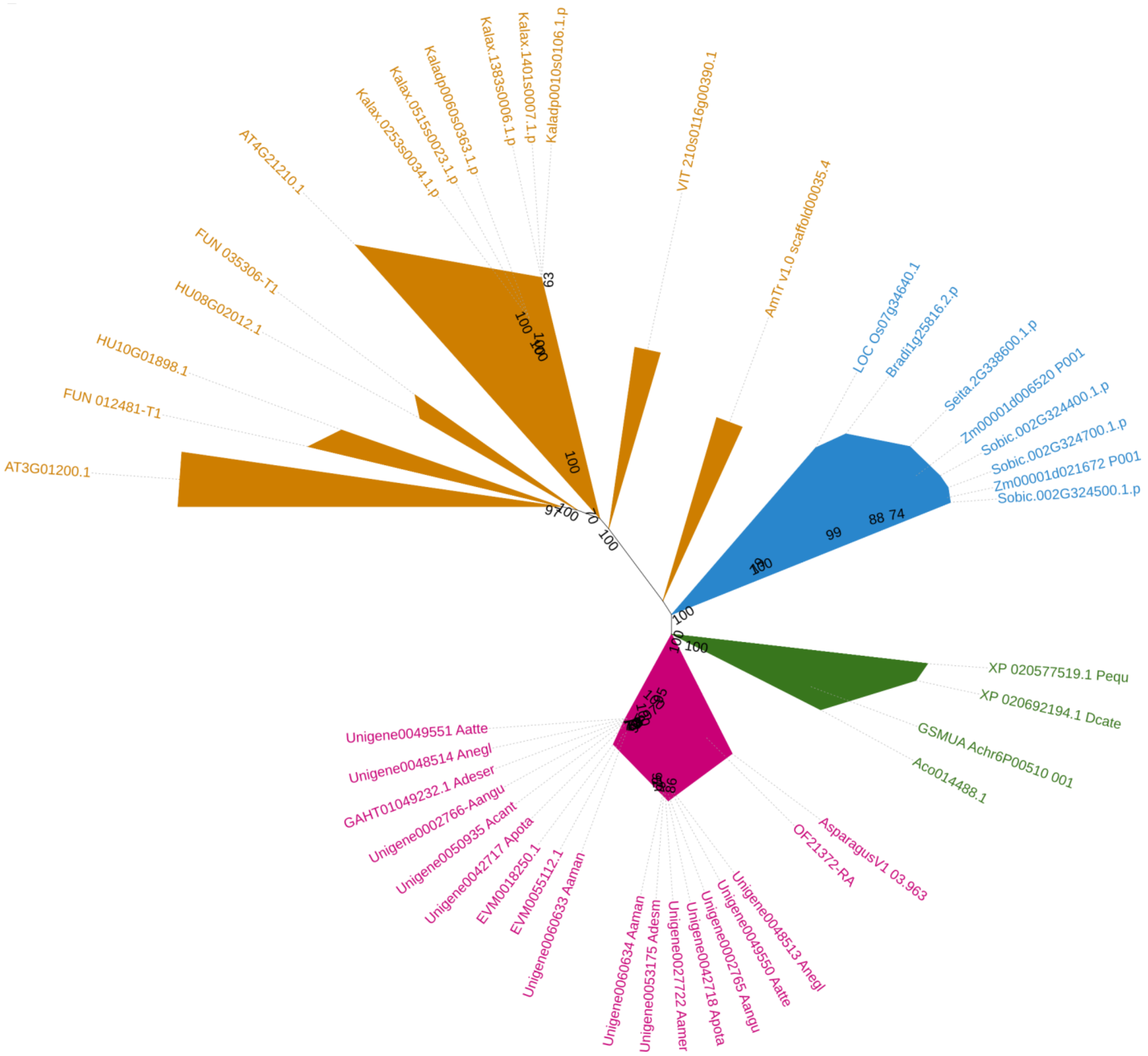

Figure S30. Phylogenetic tree of *PPDK-R* gene family. The abbreviated gene names are listed in column of information on taxa included in this study in Table S29. Asparagaceae, Orchidaceae, dicotyledons, and Poales (monocotyledons) are respectively marked in red, green, brown, and blue. Among these, Asparagaceae and Orchidaceae belong to Asparagales.

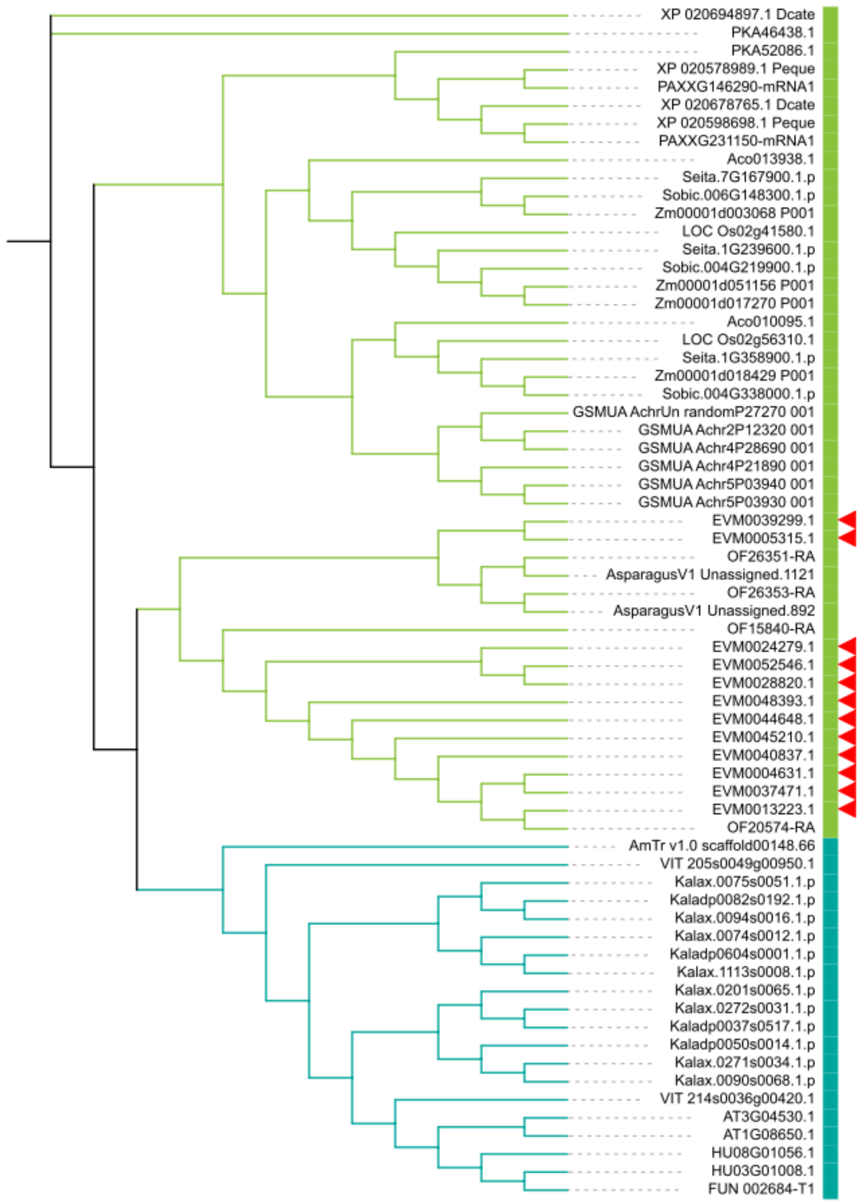

Figure S31. Phylogenetic tree of *PEPCK* gene family of *A. hybrid*. The abbreviated gene names are listed in column of information on taxa included in this study in Table S29. D and P are the initial letters of Dicotyledons and Poales.

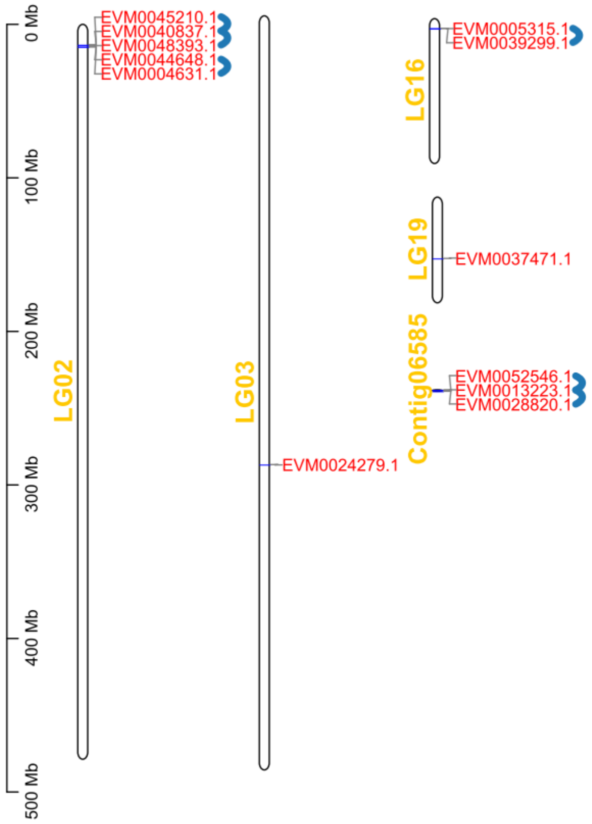

Figure S32. Distribution of *PEPCK* genes on the 4 chromosomes of *A.hybrid* genome. Blue are indicates tandem repeats, respectively.

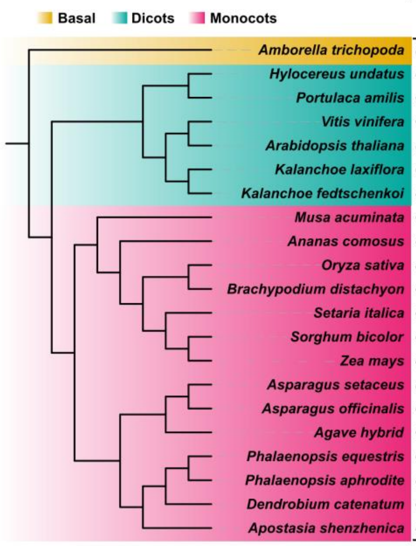

Species		Gain/Loss	αCA	βCA	γCA	NADP-MDH	NAD-MDH	NADP-ME	NAD-ME	PEPC	PEPCK	PEPC-R	PPCK	PPDK	PPDK-R
Amborella trichopoda	C3	+5/-76	+2/-5	+0/-6	+0/-10	+1/-12	+1/-21	+0/-0	+0/-6	+0/-11	+0/-0	+0/-5	+2/-0	+0/-0	+0/-0
Hylocereus undatus	CAM	+5/-7	+1/-2	+1/-0	+0/-1	+1/-0	+0/-1	+0/-0	+1/0	+1/-2	+0/-0	+0/-1	+0/-0	+0/-0	+0/-0
Portulaca amilis	CAM	+6/-13	+3/-2	+2/-2	+0/-1	+0/-3	+1/-0	+0/-3	+0/-1	+0/-0	+0/-1	+0/-0	+0/-0	+0/-0	+0/-0
Vitis vinifera	C3	+1/-29	+0/-3	+1/-4	+0/-3	+0/-3	+0/-4	+0/-3	+0/-2	+0/-3	+0/-1	+0/-1	NS/NS	+0/-0	+0/-2
Arabidopsis thaliana	C3	+13/-31	+4/-4	+1/-4	+2/-2	+1/-3	+1/-6	+1/-2	+0/-2	+1/-3	+1/-2	+0/-2	+1/-0	+0/-0	+0/-1
Kalanchoe laxiflora	CAM	+14/-4	+1/-0	+2/-1	+0/-0	+0/-0	+3/-1	+1/-0	+2/-1	+2/-0	+1/-0	+1/-1	+1/-0	+0/-0	+0/-0
Kalanchoe fedtschenkoi	CAM	+0/-37	+0/-3	+0/-4	+0/-5	+0/-4	+0/-5	+0/-3	+0/-3	+0/-2	+0/-3	+0/-0	+0/-2	+0/-1	+0/-2
Musa acuminata	C3	+21/-45	+3/-5	+1/-1	+1/-5	+0/-5	+3/-14	+1/-1	+0/-3	+3/-6	+5/-1	+0/-1	+2/-2	+2/-0	+0/-1
Ananas comosus	CAM	+1/-32	+0/-3	+0/-1	+1/-3	+0/-3	+0/-12	+0/-2	+0/-1	+0/-5	+0/-0	+0/-0	+0/-1	+0/-0	+0/-1
Oryza sativa	C3	+2/-23	+2/-4	+0/-2	+0/-3	+0/-2	+0/-4	+0/-2	+0/-1	+0/-1	+0/-1	+0/-2	+0/-1	+0/-0	+0/-0
Brachypodium distachyon	C3	+2/-30	+0/-4	+0/-1	+0/-4	+0/-2	+1/-7	+1/-2	+0/-1	+0/-4	NS/NS	+0/-2	+0/-1	+0/-1	+0/-0
Setaria italica	C4	+1/-19	+0/-5	+0/-0	+0/-0	+0/-3	+1/-8	+0/-1	+0/-0	+0/-2	+0/-0	+0/-0	+0/-0	+0/-0	+0/-0
Sorghum bicolor	C4	+3/-15	+2/-3	+0/-1	+0/-0	+0/-2	+0/-5	+0/-1	+0/-0	+0/-2	+0/-0	+0/-0	+0/-0	+0/-1	+1/-0
Zea mays	C4	+6/-14	+1/-3	+2/-1	+0/-0	+0/-2	+0/-3	+1/-2	+0/-0	+0/-2	+1/-0	+0/-0	+1/-0	+0/-1	+0/-0
Asparagus setaceus	C3	+2/-8	+0/-2	+0/-0	+0/-1	+0/-3	+2/-1	+0/-1	+0/-0	+0/-0	+0/-0	NS/NS	+0/-0	+0/-0	+0/-0
Asparagus officinalis	C3	+4/-8	+4/-0	+0/-0	+0/-2	+0/-0	+0/-2	+0/-0	+0/-0	+0/-0	+0/-2	+0/-2	+0/-0	+0/-0	+0/-0
Agave hybrid	CAM	+40/-14	+5/-1	+5/-0	+5/-3	+1/-3	+9/-2	+5/-1	+1/-1	+2/-2	+3/-1	NS/NS	+2/-0	+1/-0	+1/-0
Phalaenopsis equestris	CAM	+1/-4	+1/-3	+0/-0	+0/-0	+0/-0	+0/-0	+0/-0	+0/-0	+0/-0	+0/-0	NS/NS	+0/-0	+0/-0	+0/-1
Phalaenopsis aphrodite	CAM	+1/-4	+1/-0	+0/-1	+0/-0	+0/-0	+0/-1	+0/-0	+0/-0	+0/-0	+0/-0	+0/-1	+0/-0	+0/-0	+0/-1
Dendrobium catenatum	C3/CAM	+6/-6	+1/-1	+2/-1	+0/-0	+0/-0	+0/-0	+0/-0	+0/-0	+0/-0	+0/-1	+3/-1	+0/-1	+0/-0	+0/-1
Apostasia shenzhenica	C3	+2/-11	+1/-2	+0/-1	+0/-0	+0/-2	+0/-2	+1/-1	+0/-0	+0/-0	+0/-0	+0/-1	+0/-2	+0/-0	NS/NS

Figure S33. Statistics of gene gain and loss in 21 species. The numbers of gene gained and loss is indicated in yellow and blue, respectively. Branches of different groups of species in the species tree are marked with corresponding colors. As the gene count is zero, it is not feasible to analyze gene gain and loss, which is indicated as NS in the table.

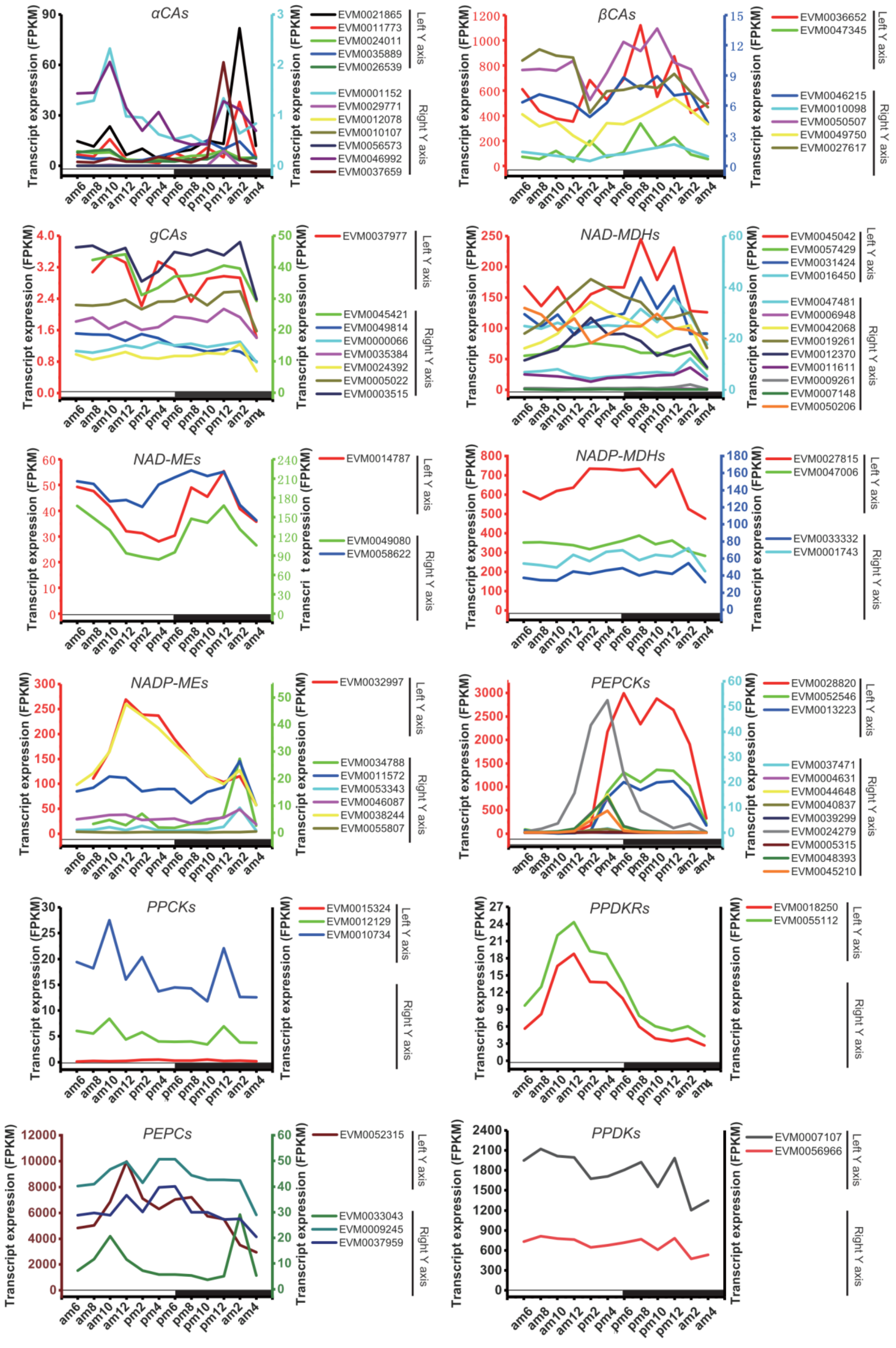

Figure S34. Key genes of the CAM pathway and their diel expression profiles. White and black bars indicate daytime (12-h) and nighttime (12-h), respectively.

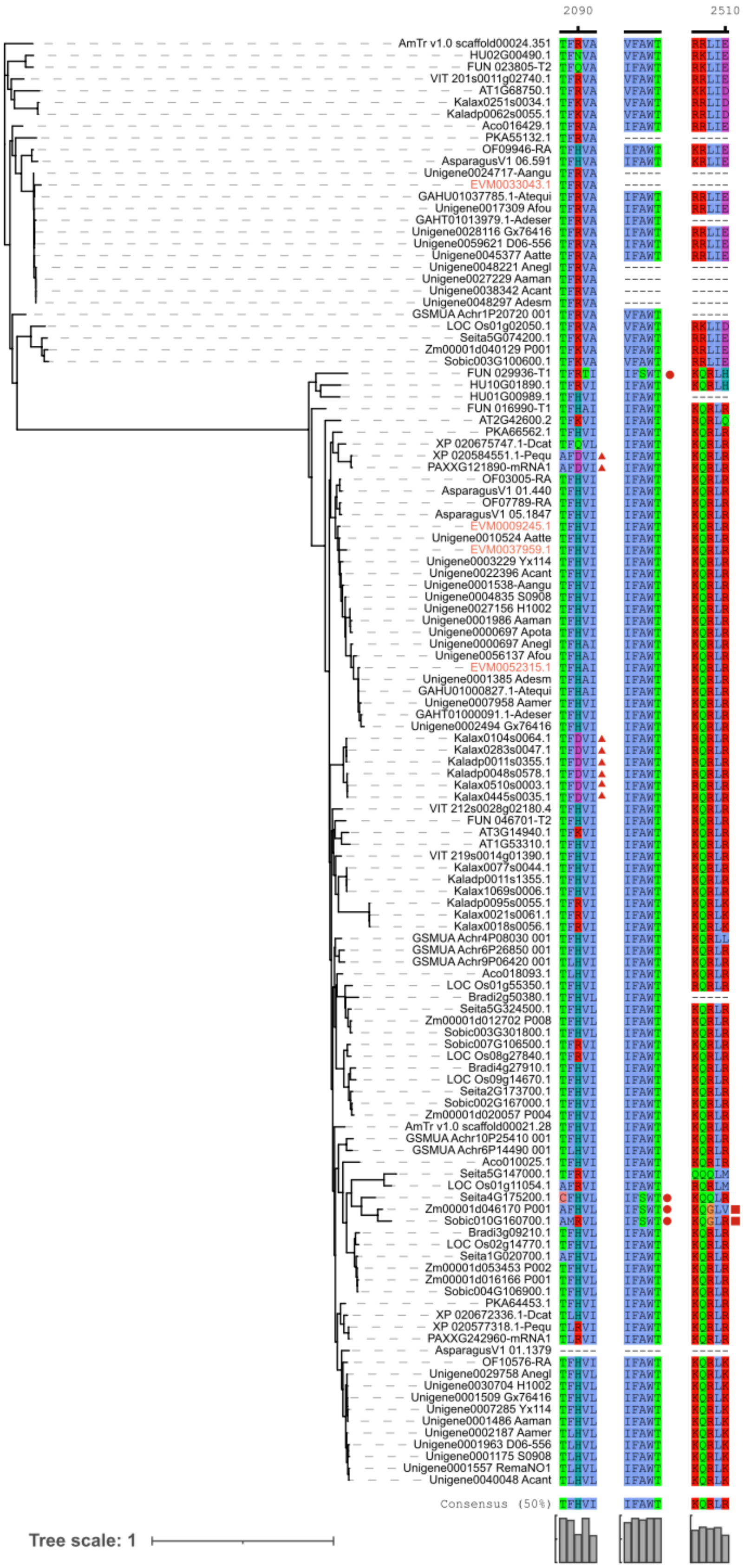

Figure S35. A maximum-likelihood phylogeny of phosphoenolpyruvate carboxylase (PEPC). The red triangle, red circular, and red square indicate the protein sequence alignment positions where the mutations (H/K/R to D, A to S, and R to G) occurred. The taxon names in the phylogenetic tree are listed in Supplementary Table 29.

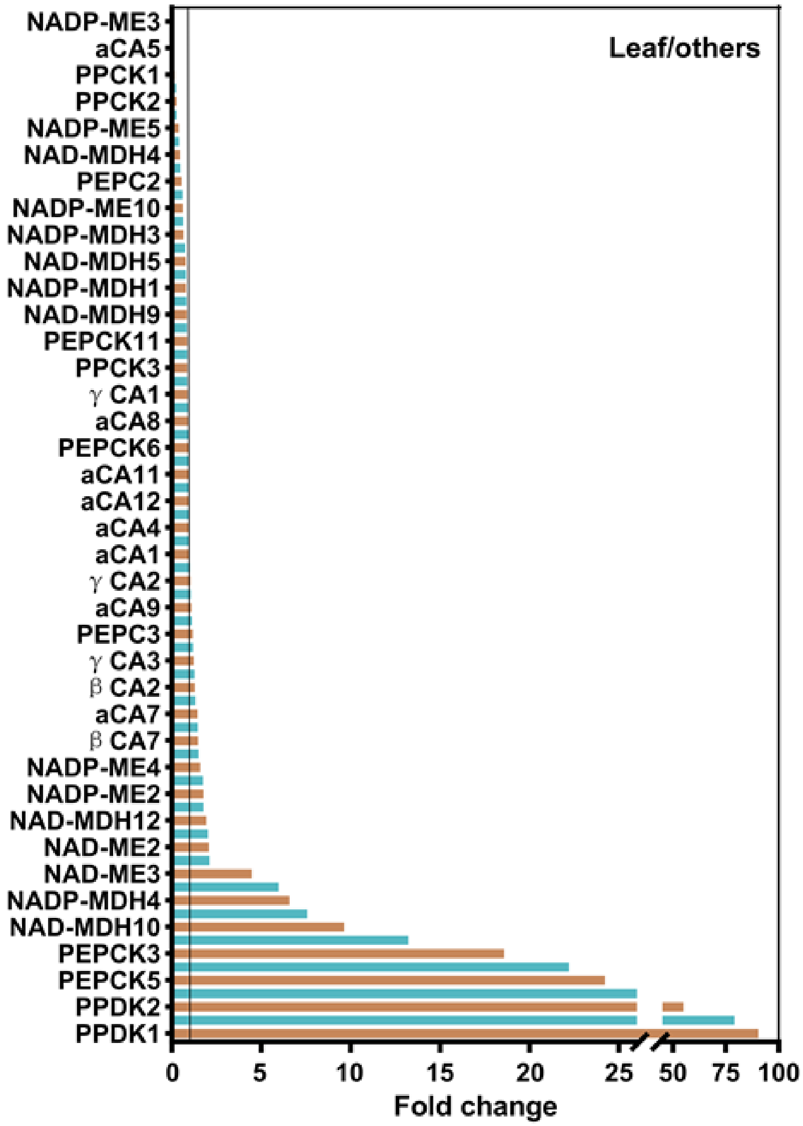

Figure S36. Expression of gene families in *A. hybrid* in leaf and other tissues. Bars indicate the expressional fold change of leaf/others calculated as (leaf + 10)/(others + 10).

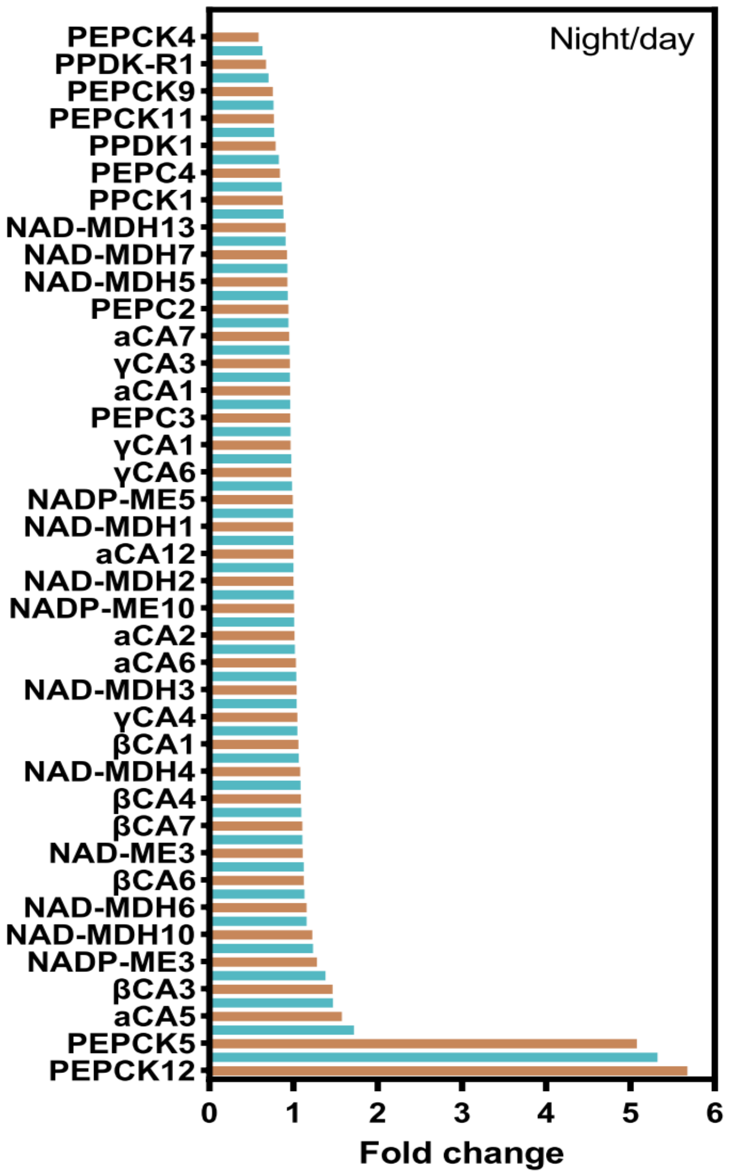

Figure S37. Expression of gene families in *A. hybrid* in night and day. Bars indicate the expressional fold change of night/day calculated as (night + 10)/(day + 10).

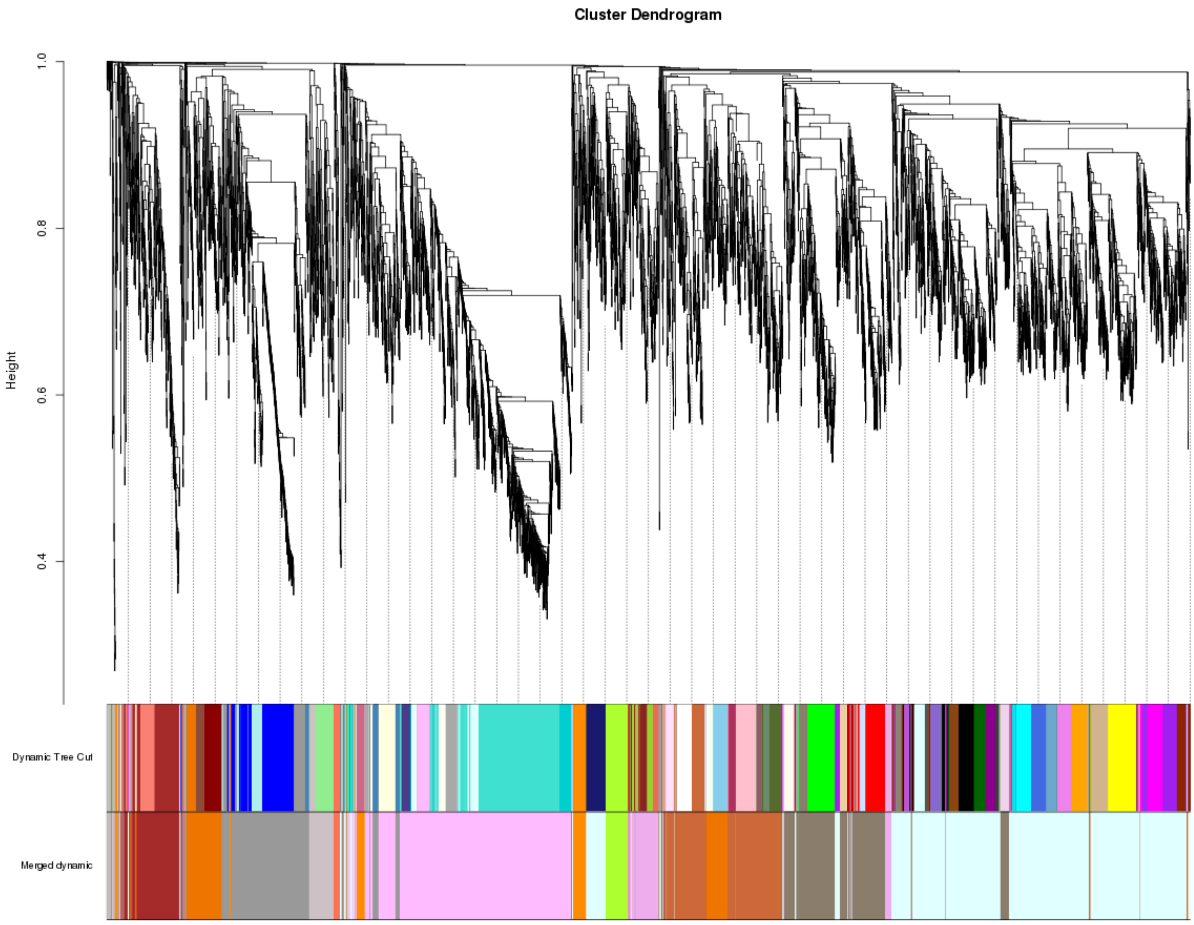

Figure S38. Clustering dendrogram of genes and merged module colors. A total of 8468 transcriptions resulted in 13 distinct modules were labeled by different merged colors.

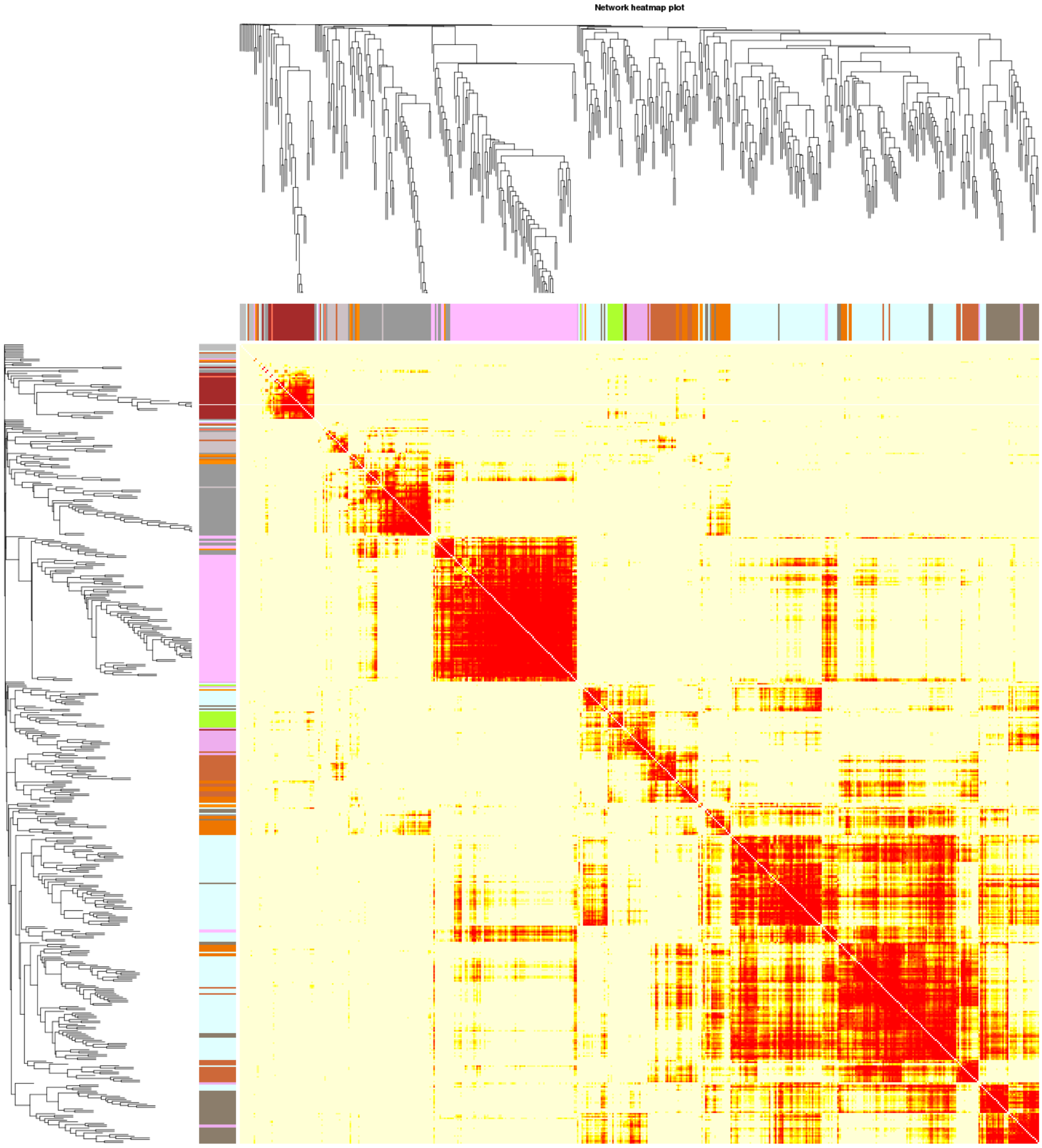

Figure S39. Hierarchical cluster tree and the relationship between gene co-expression modules (heatmap).

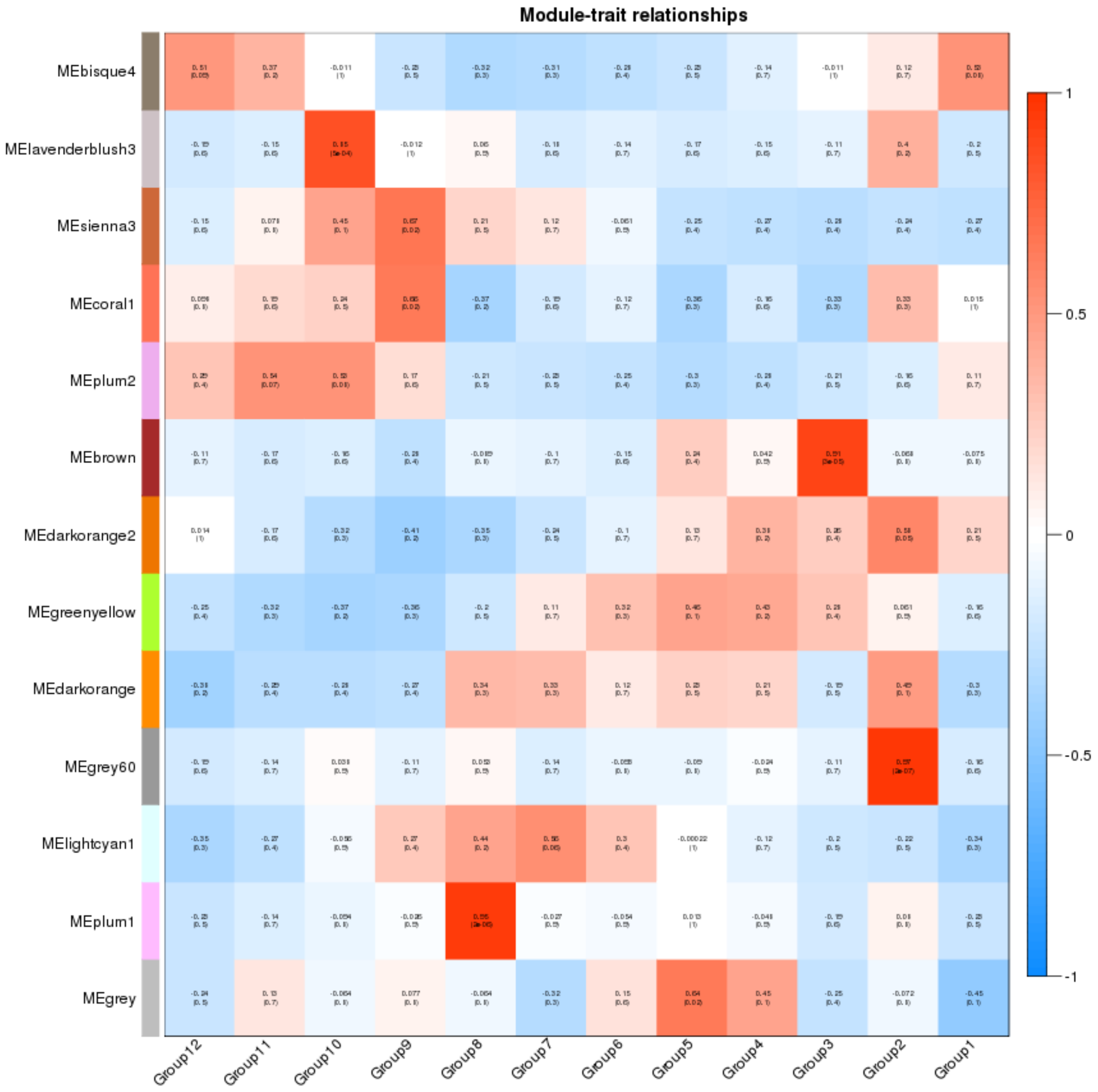

Figure S40. Module-phenolics weight correlations. Group 1, 4 am; Group 2, 2 am; Group 3, 12 pm; Group 4, 10 pm; Group 5, 8 pm; Group 6, 6 pm; Group 7, 4 pm; Group 8, 2 pm; Group 9, 12 am; Group 10, 10 pm; Group 11, 8 am; Group 12, 6 am.

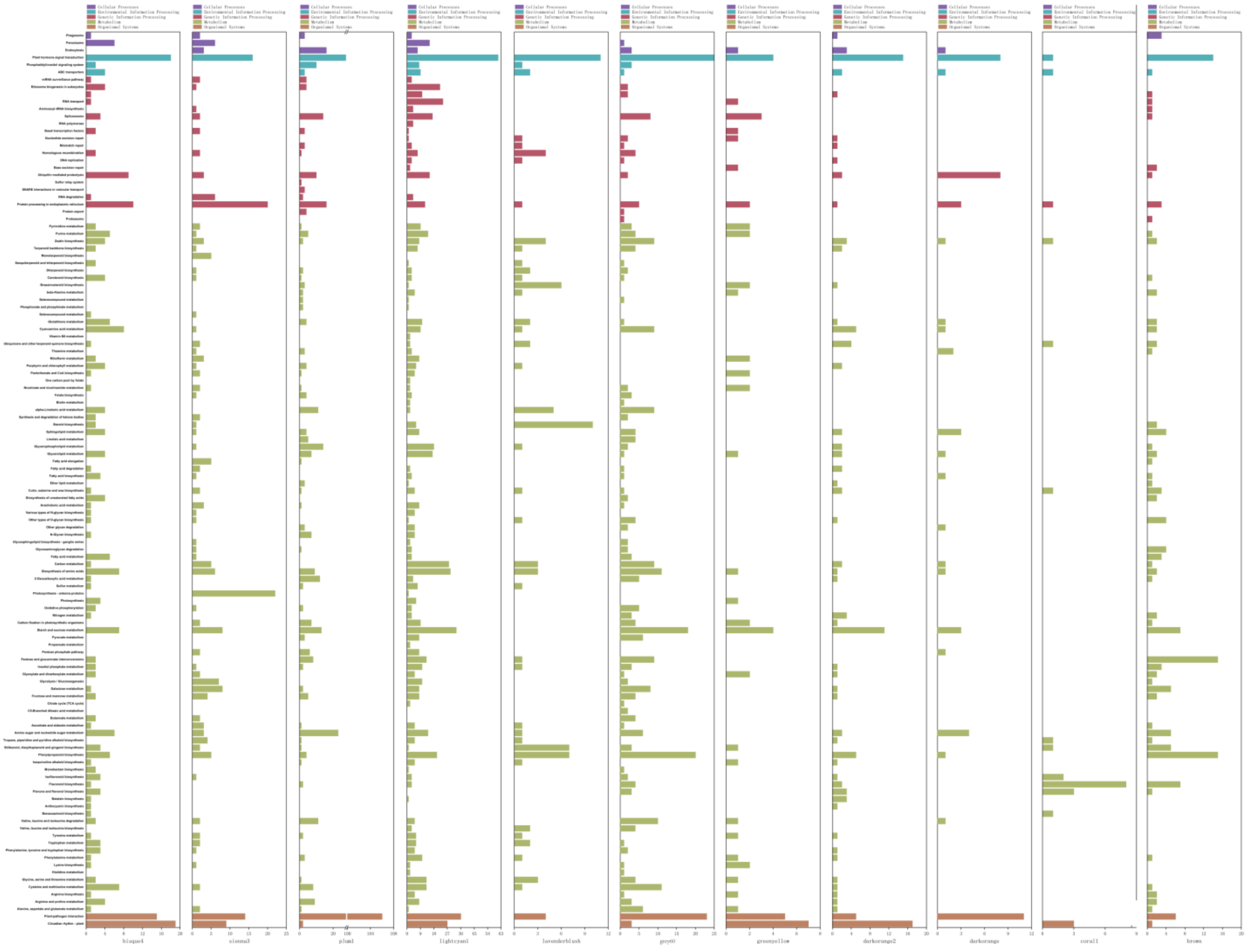

Figure S41. KEGG classification of co-expressed genes in 11 modules (bisque4, sienna3, pluml, lightcyanl1, lavenderblush, grey60, greenyellow, darkorange2, darkorange, corall, brown, and lightblue4).

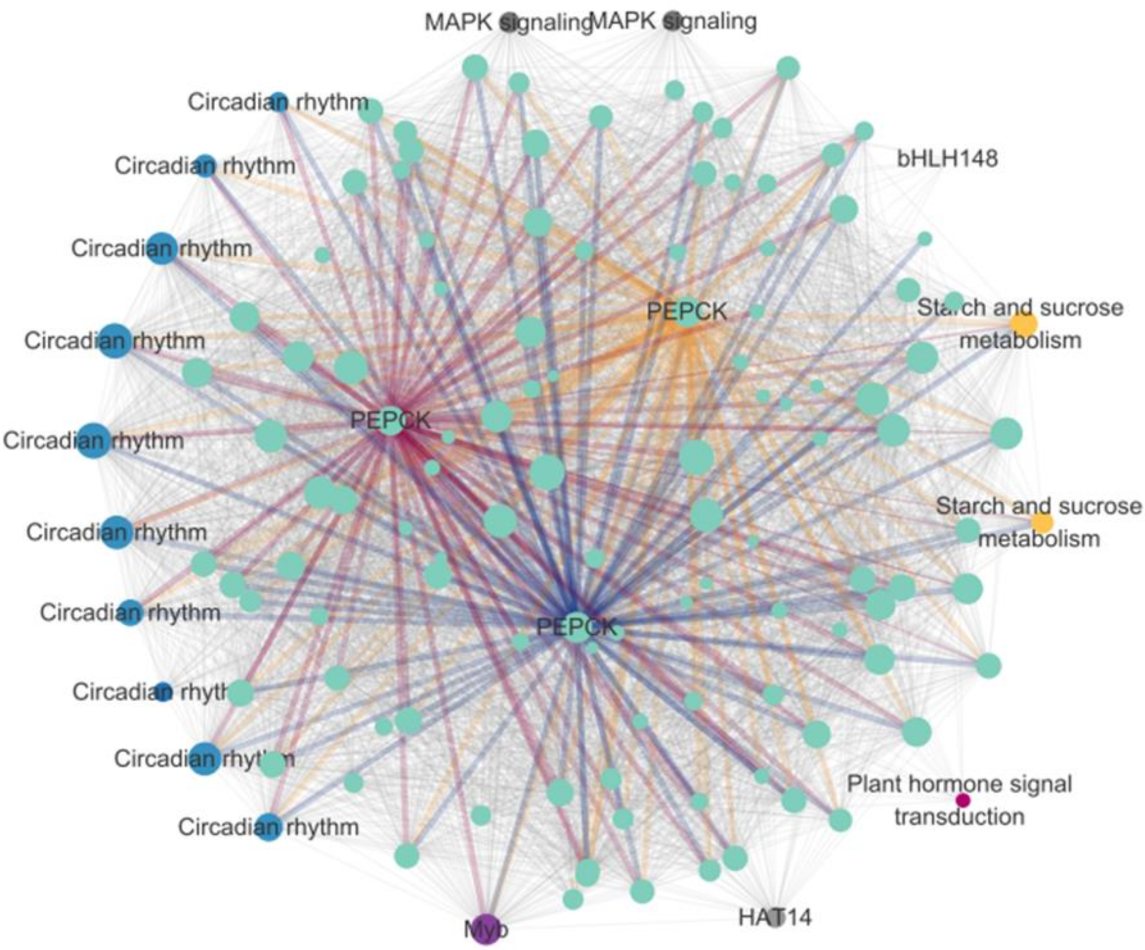

Figure S42. Cytoscape representation of co-expressed genes involved in the CAM pathway in the greenyellow modules. Modules were identified by WGCNA. The size of solid circles indicates the degree of each gene. Key genes are visualized by different colors.

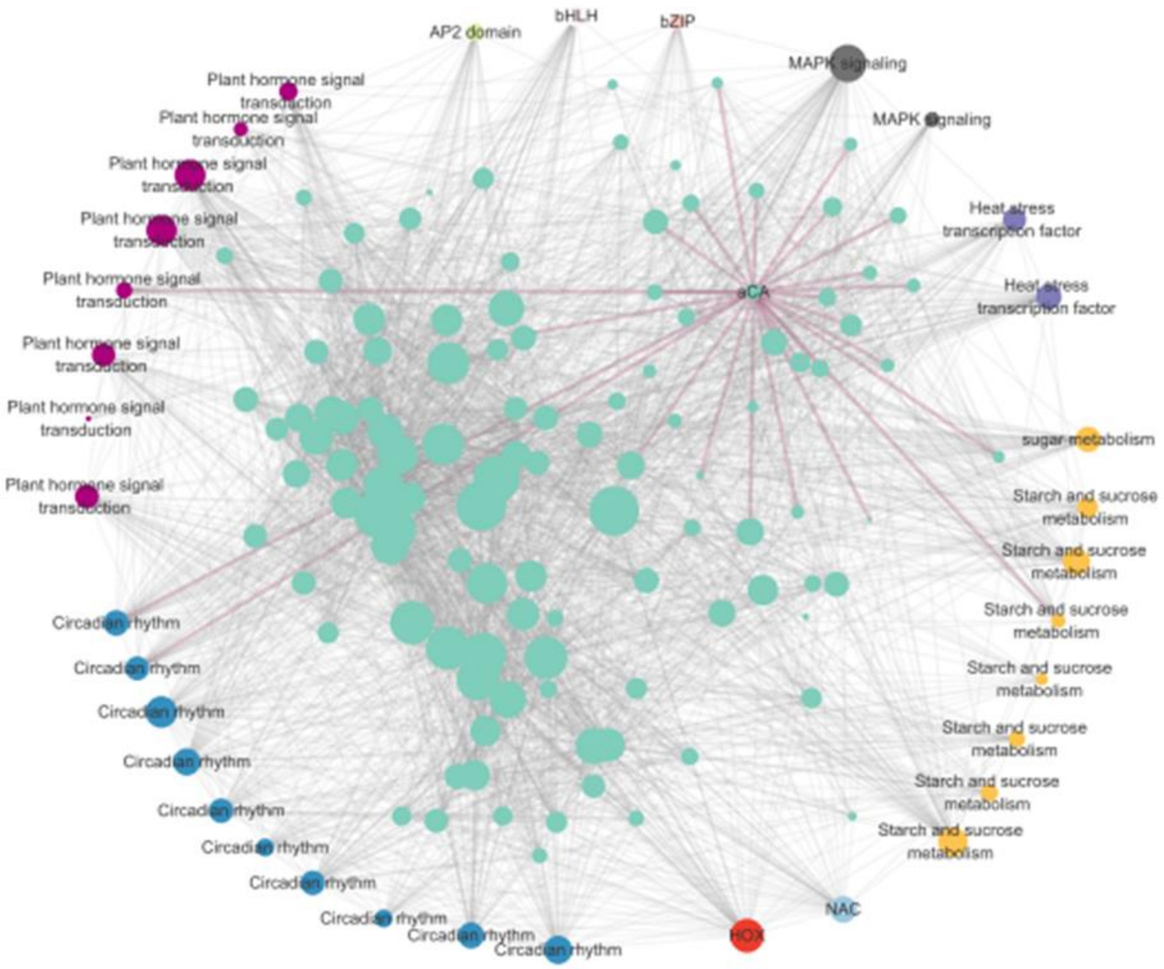

Figure S43. Cytoscape representation of co-expressed genes involved in the CAM pathway in darkorange2. Modules were identified by WGCNA. The size of solid circles indicates the degree of each gene. Key genes are visualized by different colors.

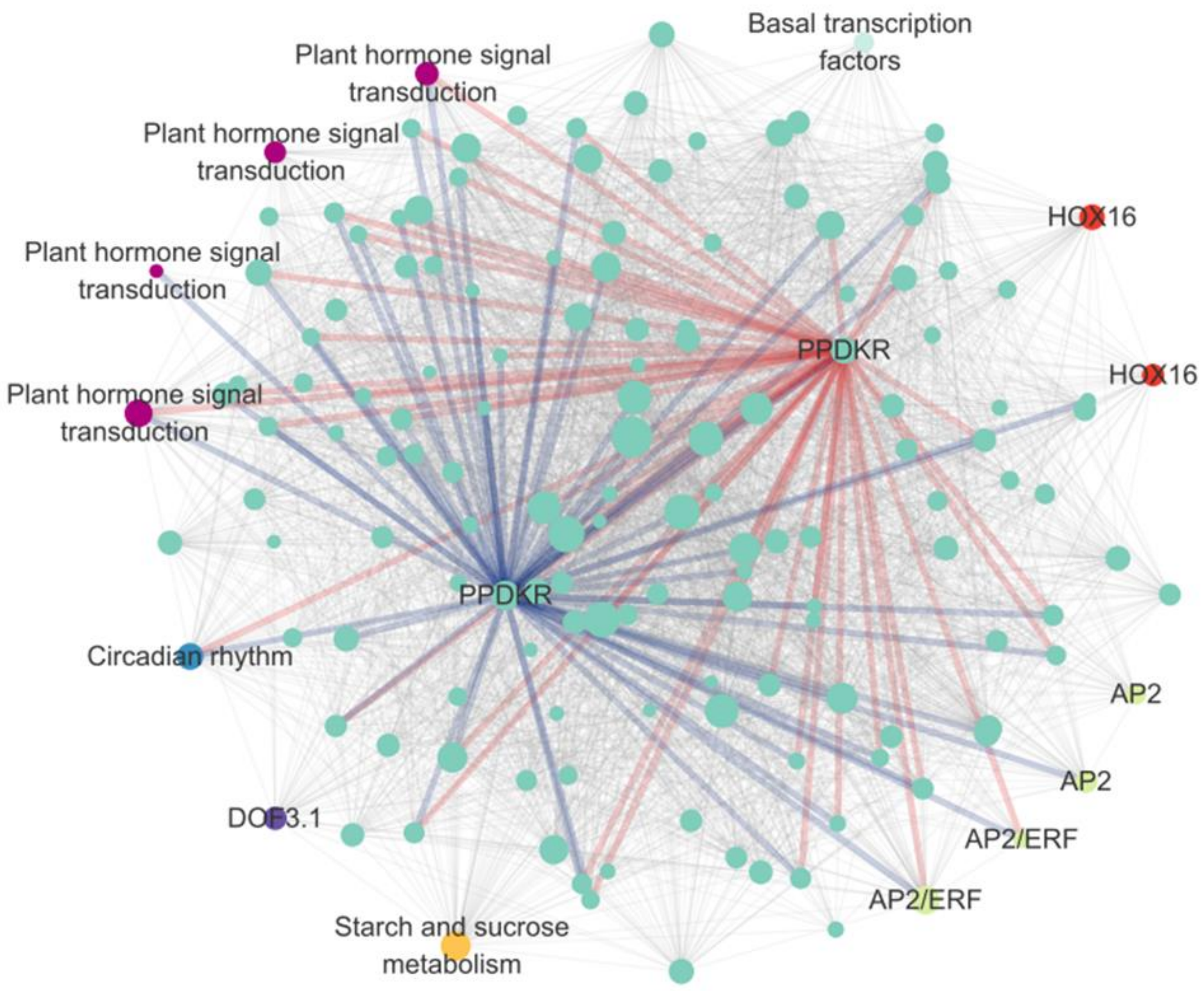

Figure S44. Cytoscape representation of co-expressed genes involved in the CAM pathway in sienna3. Modules were identified by WGCNA. The size of solid circles indicates the degree of each gene. Key genes are visualized by different colors.

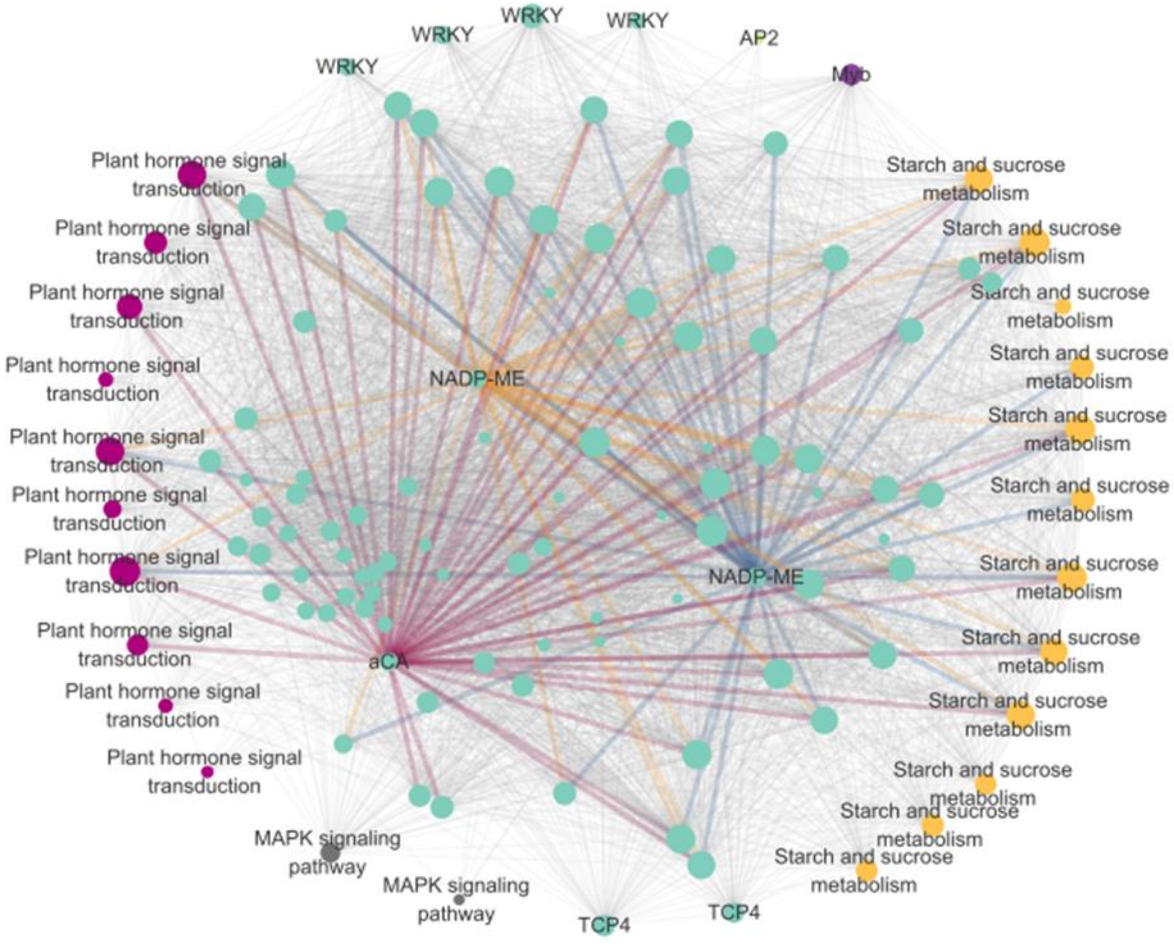

Figure S45. Cytoscape representation of co-expressed genes involved in the CAM pathway in grey60. Modules were identified by WGCNA. The size of solid circles indicates the degree of each gene. Key genes are visualized by different colors.

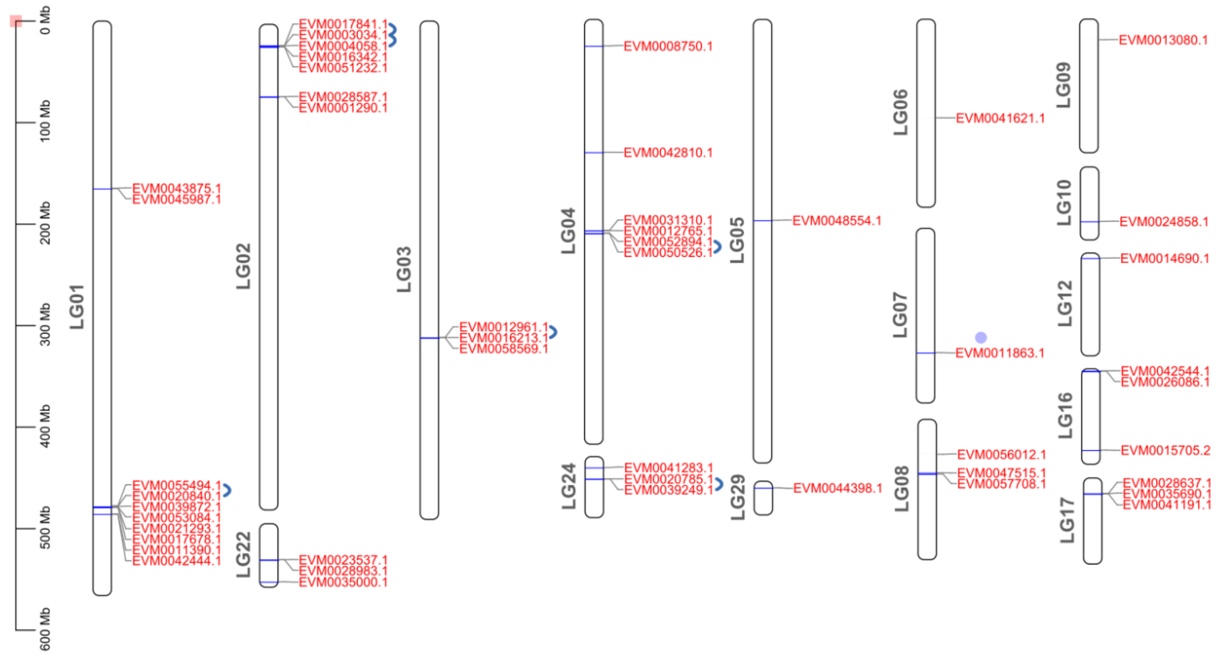

Figure S46. Distribution of *YUCCA* genes on the 16 chromosomes of *A. hybrid* genome. Blue are indicates tandem repeats, respectively.

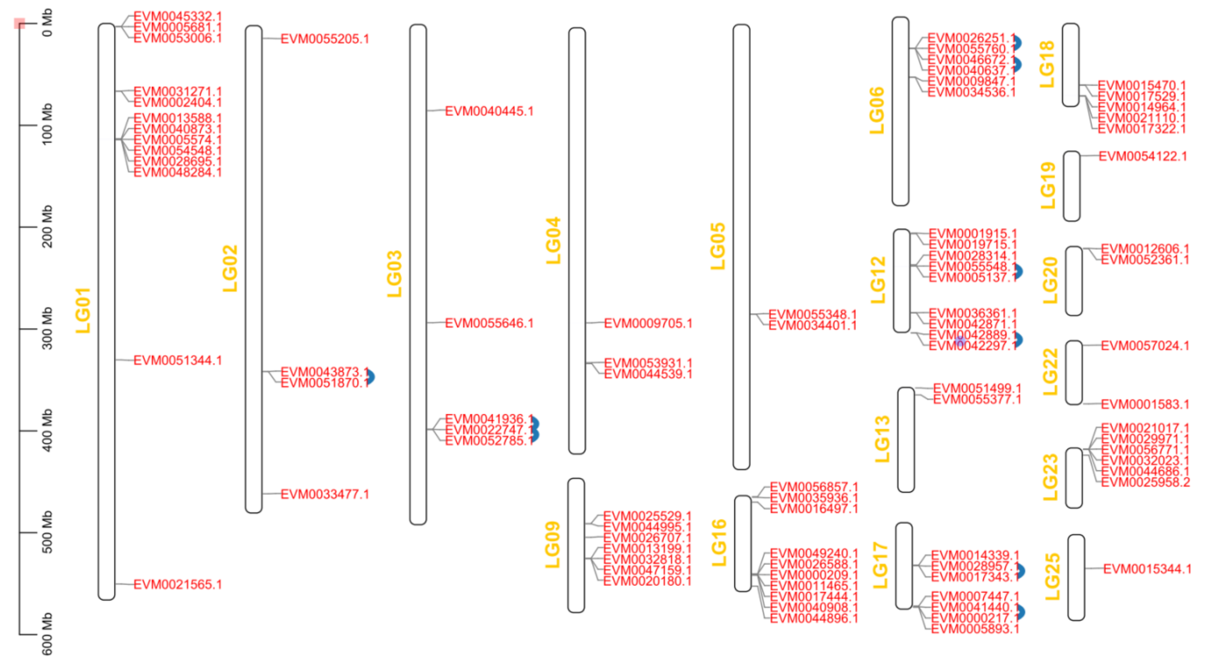

Figure S47. Distribution of *SAUR* genes on the 17 chromosomes of *A. hybrid* genome. Blue are indicates tandem repeats, respectively.

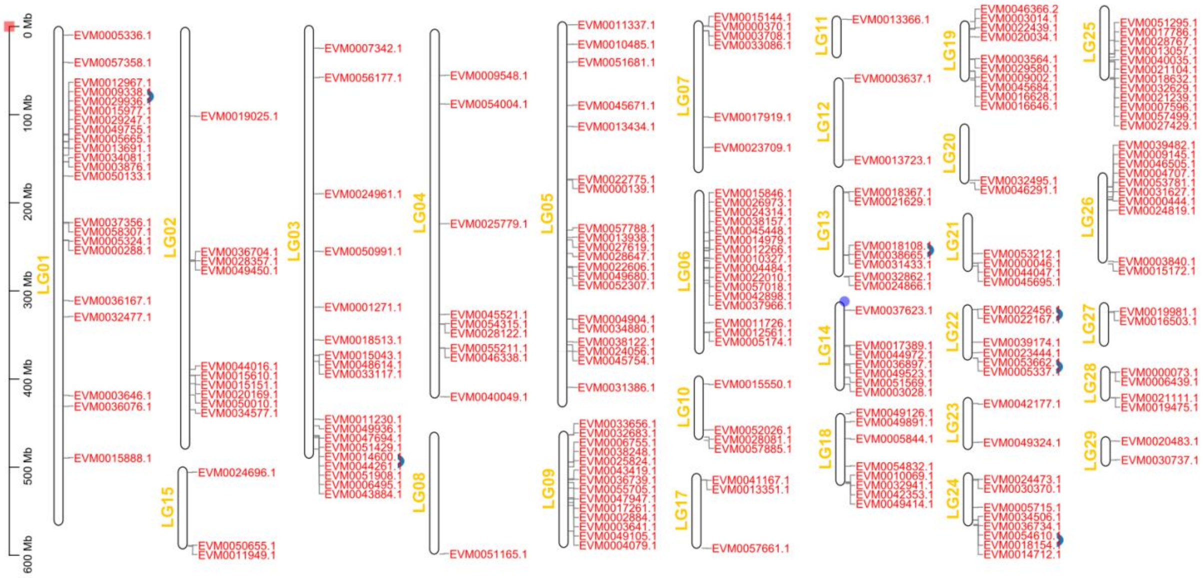

Figure S48. Distribution of *ERF* genes on the 28 chromosomes of *A. hybrid* genome. Blue are indicates tandem repeats, respectively.

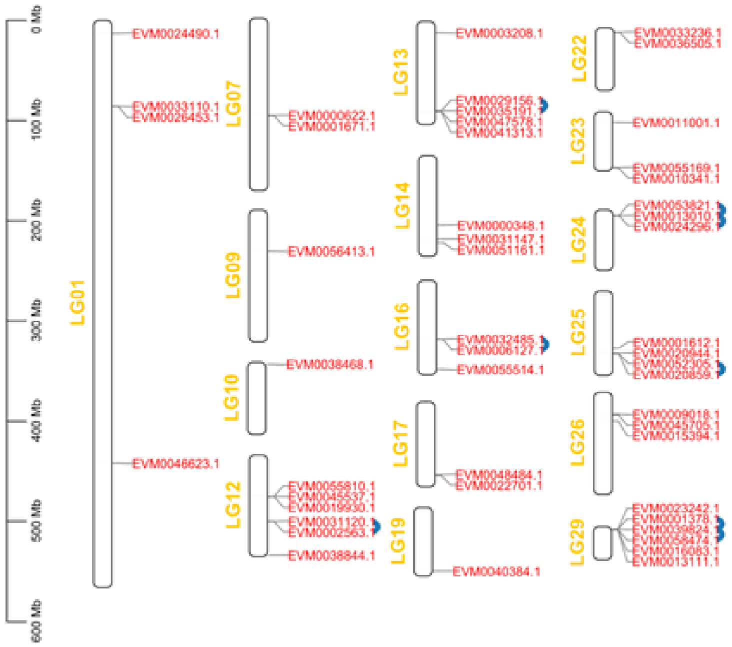

Figure S49. Distribution of *LOX* genes on the 16 chromosomes of *A. hybrid* genome. Blue are indicates tandem repeats, respectively.

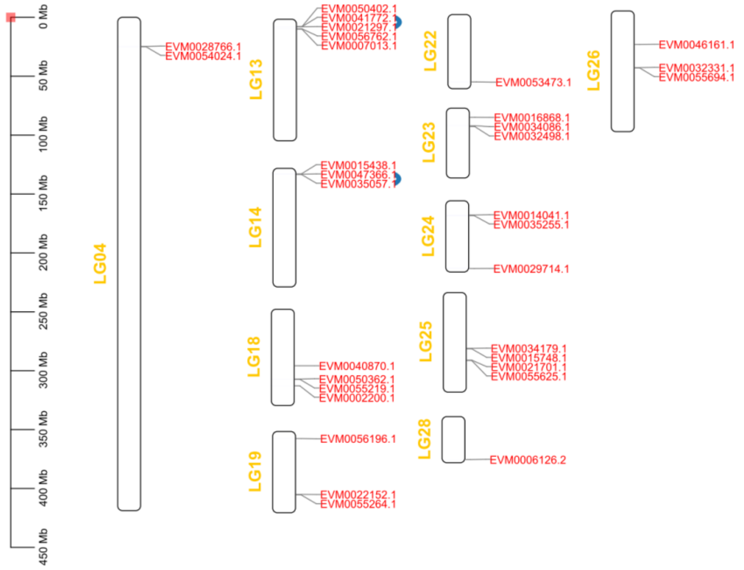

Figure S50. Distribution of *JAZ* genes on the 11 chromosomes of *A. hybrid* genome. Blue are indicates tandem repeats, respectively.

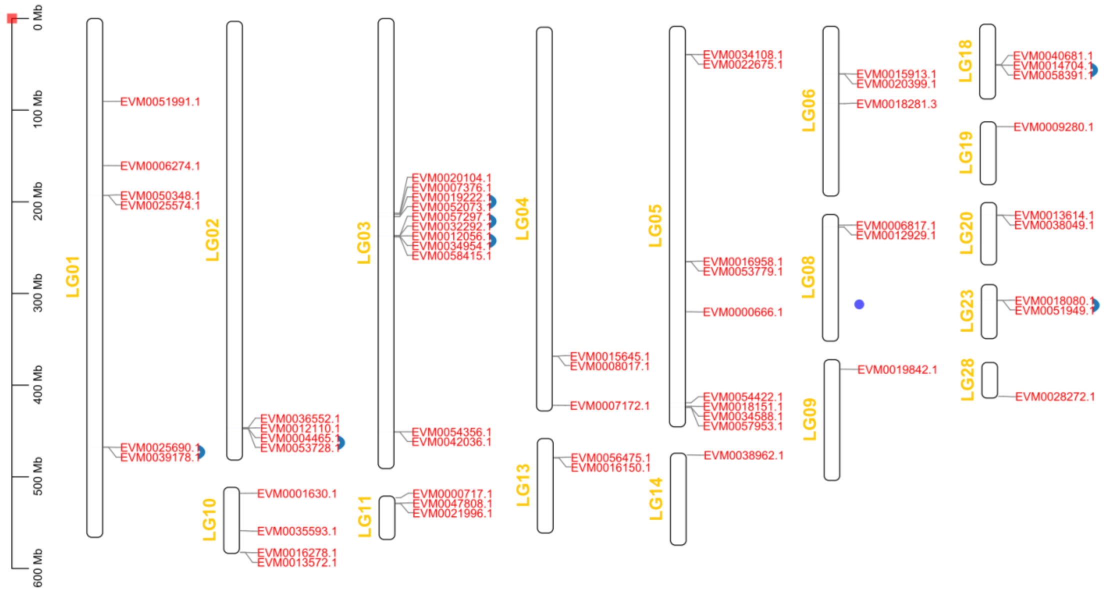

Figure S51. Distribution of the *SWEET* genes on the 17 chromosomes of the *A. hybrid* genome. Blue are indicates tandem repeats, respectively.

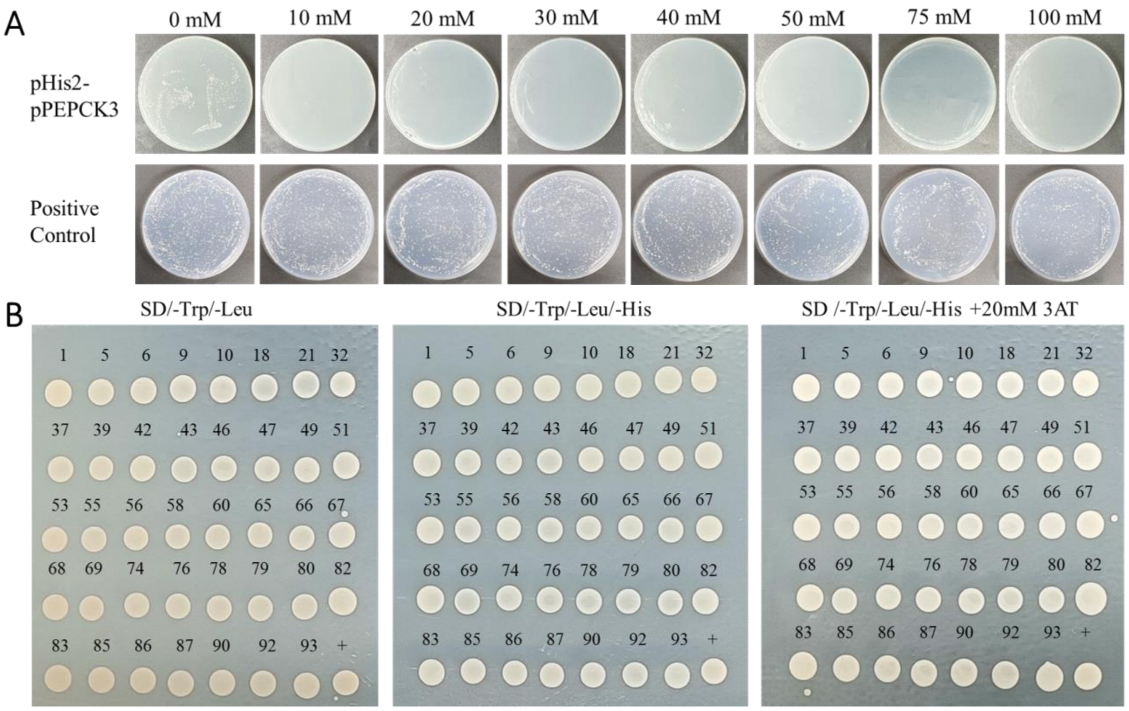

Figure S52. Yeast one-hybrid assay for screening transcription factors. (A) 3-amino-1,2,4-triazole (3-AT) concentration testing. Yeast contained pHis2-*pPEPCK3* grown on selective medium with different concentrations of 3AT (0, 10, 20, 30, 40, 50, 75, 100 mM) to determine the inhibitory concentration of 3AT. (**B**) Confirmation of positive interaction. The interaction between positive plasmid and bait plasmid were tested in yeast on selective medium (SD/-Trp/-Leu, SD/-Trp/-Leu/-His, SD/-Trp/-Leu/-His +20mM 3AT). Positive control and plus signs (+) are indicates positive control strain, which contained p53His2 and pGAD-Rec-p53 vector.

References

1. Cai, J. et al. The genome sequence of the orchid *Phalaenopsis equestris*. *Nat. Genet.* **47**, 65-72 (2015).

2. Chao, Y. T. et al. Chromosome-level assembly, genetic and physical mapping of *Phalaenopsis aphrodite* genome provides new insights into species adaptation and resources for orchid breeding. *Plant Biotechnol. J.* **16**, 2027-2041 (2018).

3. Chen, J. Y. et al. A chromosome-scale genome sequence of pitaya (*Hylocereus undatus*) provides novel insights into the genome evolution and regulation of betalain biosynthesis. *Hortic. Res*. **8**, 164 (2021).

4. Gross, S. M et al. De novo transcriptome assembly of drought tolerant CAM plants, *Agave deserti* and *Agave tequilana*. *BMC Genomics* **14**, 563 (2013).

5. Li, S. F. et al. Chromosome-level genome assembly, annotation and evolutionary analysis of the ornamental plant *Asparagus setaceus*. *Hortic. Res.* **7**, 48 (2020).

6. Sarwar, M. B. et al. De novo assembly of *Agave sisalana* transcriptome in response to drought stress provides insight into the tolerance mechanisms. *Sci. Rep*. **9**, 396 (2019).
